# Chemical Orderings in CuCo Nanoparticles: Topological Modeling Using DFT Calculations

**DOI:** 10.3390/nano14151242

**Published:** 2024-07-24

**Authors:** Konstantin M. Neyman, Pere Alemany

**Affiliations:** 1 ICREA (Institució Catalana de Recerca i Estudis Avançats), Pg. Lluís Companys 23, 08010 Barcelona, Spain; 2 Departament de Ciència de Materials i Química Física and Institut de Quimica Teòrica i Computacional (IQTCUB), Universitat de Barcelona, c/Martí i Franquès 1, 08028 Barcelona, Spain; p.alemany@ub.edu

**Keywords:** magnetic bimetallic nanoparticles, chemical ordering, density functional calculations, single-atom alloy catalysts, CO adsorption, C-O vibrational frequencies

## Abstract

The orderings of atoms in bimetallic 1.6–2.1 nm-large CuCo nanoparticles, important as catalytic and magnetic materials, were studied using a combination of DFT calculations with a topological approach. The structure and magnetism of Cu_50_Co_151_, Cu_101_Co_100_, Cu_151_Co_50_, and Cu_303_Co_102_ nanoparticles; their resistance to disintegrating into separate Cu and Co species; as well as the exposed surface sites, were quantified and analyzed, showing a clear preference for Cu atoms to occupy surface positions while the Co atoms tended to form a compact cluster in the interior of the nanoparticles. The surface segregation of Co atoms that are encapsulated by less-active Cu atoms, induced by the adsorption of CO molecules, was already enabled at a low coverage of adsorbed CO, providing the energy required to displace the entire compact Co species inside the Cu matrices due to a notable adsorption preference of CO for the Co sites over the Cu ones. The calculated adsorption energies and vibrational frequencies of adsorbed CO should be helpful indicators for experimentally monitoring the nature of the surface sites of CuCo nanoparticles, especially in the case of active Co surface sites emerging in the presence of CO.

## 1. Introduction

Bimetallic alloys are important materials for science and technology, with nanostructures exhibiting interesting size-dependent properties [1,2,3,4]. CuCo is an example of a bimetallic system with a broad miscibility gap over wide compositional and temperature ranges [2]. As a result, CuCo alloys unstable at high temperatures can separate into Cu and Co components [3]. Importantly, the magnetism of CuCo nanoparticles (NPs) enables the preparation of magneto-resistive materials [4]. The catalytic activity of CoCu alloys [5,6,7,8,9,10,11,12,13,14,15] is of primary relevance for the present study. To this end, single-atom alloy catalysts should be mentioned that may show, along with a high activity, extraordinary selectivity due to the exposure of just a single type of active sites that are formed by one atom of an active metal surrounded by less active atoms [16,17,18,19,20,21].

The present study aims to provide a detailed determination of the surface structure of CuCo NPs, which defines their reactivity and catalytic properties. For that purpose, we carried out Density Functional Theory (DFT) calculations of CuCo NPs with up to 405 atoms (ca. 2.1 nm large). The most energetically stable chemical orderings (i.e., the locations of the metal atoms of the two different types) in these NPs were calculated using a *Topological* (TOP) approach that was developed in our group [22,23], which allows for a global optimization of the chemical ordering in bimetallic nanoalloys to find the lowest-energy distributions of alloying atoms in a nanocrystal at a reasonable computational cost. The TOP method has been used mainly for truncated-octahedral *fcc*-type NPs, but it is also applicable for the description of bimetallic NPs with other shapes and lattices [22,23]. Previous TOP studies have characterized the chemical orderings in a number of Pd- and Pt-containing bimetallic NPs—such as PdCu [22], PdAg [22,24], PdAu [22,23,24,25], PdZn [22], PtNi [26], PtCu [27], PtAg [27], and PtAu [24,27,28] to mention only a few—with different contents of alloyed metals. Very recently, the TOP approach was extended and successfully applied to determine the most energetically stable chemical orderings in trimetallic PtNiZr NPs [29], which are efficient oxygen reduction reaction catalysts.

Among the problems addressed in the present article are: (i) defining the TOP equations and parameters determining the chemical orderings in 201-atomic (Cu_50_Co_151_, Cu_101_Co_100_, and Cu_151_Co_50_) and 405-atomic (Cu_303_Co_102_) CuCo NPs; (ii) analyzing the dependence of the energetic parameters in these TOP equations on the Cu:Co content and size of NPs; (iii) predicting the structure, magnetism, and exposed surface sites of the studied CuCo NPs; (iv) analysis of the possibility of a surface segregation of the more-active Co atoms induced by the adsorption of CO molecules; and (v) quantifying the adsorption energies and vibrational frequencies of CO adsorbates to be used for monitoring the presence of Co surface atoms on CuCo NPs.

## 2. Methods and Models

Spin-polarized DFT calculations were performed using the plane-wave code VASP [30,31]. The usage of the periodic computational method allowed for comparing, on the same footing, the results of the 0D nanoparticles and the corresponding 1D (nanowires), 2D (surface slabs), and 3D (bulk) systems. The generalized-gradient, exchange–correlation functional by Perdew, Becke, and Ernzerhof (PBE) [32] was employed in combination with the projector-augmented wave (PAW) representation of core electrons [33,34]. To moderate the computational cost, the cut-off energy for the plane-wave functions of bare metal NPs was set to 273.2 eV, which provides accurate-enough total energy differences between distinct chemical orderings of a given NP [22]. In the presence of adsorbed CO molecules, the cutoff energy was increased to 400 eV. The Brillouin zone was sampled only at the Γ-point. One-electron Kohn–Sham energy levels were smeared by 0.1 eV, and the final total energies were extrapolated to a zero smearing. During geometry optimization, all atoms were locally relaxed without restrictions until the forces acting on each atom decreased to less than 0.2 eV/nm. The vibrational frequencies of the adsorbed CO molecules were calculated via the displacements of C and O atoms by 2 pm along Cartesian coordinates.

The CuCo nanoalloys were represented by 201-atomic (Cu_50_Co_151_, Cu_101_Co_100_, and Cu_151_Co_50_) and 405-atomic (Cu_303_Co_102_) truncated-octahedral NPs with *fcc*-type crystal lattices (see Section 3.2). It should be noted that, in this size range, decahedron or icosahedron NPs could exhibit similar energetic stability as truncated-octahedral NPs. The corresponding 201- and 405-atomic monometallic Cu and Co NPs served as references. The ca. 1.6 nm-large 201-atomic NPs were placed in 2.5 × 2.5 × 2.5 nm^3^ cubic cells, whereas the ca. 2.1 nm-large 405-atomic NPs occupied 3.2 × 3.2 × 3.2 nm^3^ cubic cells, which provided a sufficient vacuum space between the periodically repeated NPs to mitigate the interactions between them [35].

**Figure 1:**
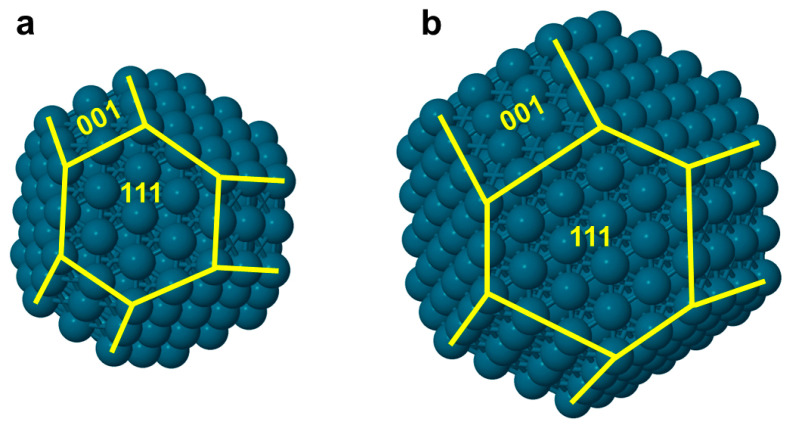
The *fcc*-type truncated-octahedral metal NPs composed of (**a**) 201 and (**b**) 405 atoms. Yellow lines indicate the atoms of some edges separating {001} and {111} nanofacets.

The 201-atomic NP (Figure 1a) comprises 948 metal–metal bonds (nearest-neighbor atoms pairs). Of its 122 surface atoms that form a monatomic thick skin, 24 atoms correspond to corners with a coordination number CN = 6, 36 are at edge sites with CN = 7, 6 occupy {001} nanofacet sites with CN = 8, and 56 occupy {111} nanofacet sites with CN = 9. The remaining 79 atoms, with CN = 12, form a truncated octahedron interior core covered by the atoms on the skin.

The 405-atomic NP (Figure 1b) features overall 2016 metal–metal bonds and a 204-atomic skin. The skin covers the just mentioned 201-atomic NP with a 122-atomic subsurface shell and two deeper core shells of 60 and 18 atoms, respectively, around the central atom. The skin contains 24 CN = 6 atoms at corner positions, 60 edge CN = 7 atoms, 24 CN = 8 atoms in the {001} nanofacets and 96 CN = 9 atoms in the {111} nanofacets.

## 3. Results and Discussion

### 3.1. Topological Equations for CuCo Nanoparticles

The energy of an alloy NP with a given stoichiometry, crystallinity, and shape depends on the chemical (atomic) ordering, i.e., on the precise locations of the atoms of the two alloying metals at different (locally relaxed) crystal lattice positions. Each distinct chemical ordering is also referred to as homotop [1]. The lowest-energy chemical orderings of the CuCo binary alloy NPs—the homotops most favorable by the DFT energy—were obtained using the TOP approach [22,23] and the corresponding computational protocols [24,27]. Accordingly, the topological total energy of a CuCo truncated-octahedral *fcc* nanocrystal with a particular ordering is as follows:

(1) $$E^{TOP}=E^{0}+\varepsilon_{BOND}^{Cu-Co}\times N_{BOND}^{Cu-Co}+\varepsilon_{COR}^{Cu}\times N_{COR}^{Cu}+\varepsilon_{EDG}^{Cu}\times N_{EDG}^{Cu}+\varepsilon_{001}^{Cu}\times N_{001}^{Cu}+\varepsilon_{111}^{Cu}\times N_{111}^{Cu},$$

where $N_{BOND}^{Cu-Co}$ is the number of Cu–Co bonds (the Cu–Co nearest-neighbor atom pairs), and $N_{COR}^{Cu}$, $N_{EDG}^{Cu}$, $N_{001}^{Cu}$, and $N_{111}^{Cu}$ are the numbers of Cu atoms located at corners (vertexes), edges, and the {001} and {111} nanofacets with a CN of 6, 7, 8, and 9, respectively. A shorter notation of Equation (1) is as follows:

(2) $$E^{TOP}=E^{0}+\varepsilon^{Cu-Co}\times N^{Cu-Co}+\varepsilon_{6}^{Cu}\times N_{6}^{Cu}+\varepsilon_{7}^{Cu}\times N_{7}^{Cu}+\varepsilon_{8}^{Cu}\times N_{8}^{Cu}+\varepsilon_{9}^{Cu}\times N_{9}^{Cu}.$$

The parameters *ε*, obtained by fitting the topological energy $E^{TOP}$ to the DFT total energies $E^{DFT}$ of several dozens of homotops [22,23], represent energy contributions of either a Cu-Co bond or a Cu atom located in a particular surface site of a NP. $E^{0}$ is a constant offset between the $E^{TOP}$ and $E^{DFT}$ energy scales, which cancels in the energy differences between two orderings (topologies) defined by the descriptors ($N^{Cu-Co}$, $N_{6}^{Cu}$, $N_{7}^{Cu}$, $N_{8}^{Cu}$, and $N_{9}^{Cu}$). *E^TOP^* is preferably expressed via the numbers of atoms of an element that is more stable on the surface of the nanoalloy than in its inner positions (especially when such atoms are not in minority); for CuCo NPs, these are Cu atoms (see below). Conversely, if *E^TOP^* is expressed via descriptors of an element that is less stable on the surface, then the training set required to reliably parameterize the TOP equations will need to include homotops with unnecessarily high energies, which were less representative of the searched lowest-energy homotops.

Each of the mentioned above ($N^{Cu-Co}$, $N_{6}^{Cu}$, $N_{7}^{Cu}$, $N_{8}^{Cu}$, and $N_{9}^{Cu}$) topologies commonly encompass many homotops with equal *E^TOP^* energies, but somewhat different *E^DFT^* energies. To distinguish some of the groups of homotops of a given typology ($N^{Cu-Co}$, $N_{6}^{Cu}$, $N_{7}^{Cu}$, $N_{8}^{Cu}$, and $N_{9}^{Cu}$), one can classify homotops also by the number of homometallic bonds between the atoms of one of the alloyed metals. The descriptor $N^{Cu-Co}$ along with the total number of bonds, $N=N^{Cu-Co}+N^{Cu-Cu}+N^{Co-Co}$, fixed by the morphology of the NP defines the sum $N^{Cu-Cu}+N^{Co-Co}$, but not the individual terms. To avoid this ambiguity, the ($N^{Cu-Co}$, $N_{6}^{Cu}$, $N_{7}^{Cu}$, $N_{8}^{Cu}$, $N_{9}^{Cu}$, and $N^{Co-Co}$) descriptor extended by the $N^{Co-Co}$ term was used herein to classify the CuCo NP homotops’ topologies.

The TOP parameters in Equation (2) for the CuCo NPs under scrutiny fitted to *E^DFT^* data following the previously established protocols [22,23,24,27] (see also Supplementary Materials) are listed in Table 1. The positive $\varepsilon^{Cu-Co}$ terms for all the NPs pointed to an energetically unfavorable mixing (alloying) of Cu and Co atoms to form Cu-Co bonds. Being quite big in magnitude, negative $\varepsilon_{i}^{Cu}$ terms suggest a strong preference in Cu atoms occupying lower-coordinated surface (skin) positions, whereas Co atoms prefer the CN = 12 inner positions in the CuCo NPs.

### 3.2. Chemical Orderings in the Cu_201-k_Co_k_ and Cu_303_Co_102_ Nanoparticles

**Figure 2:**
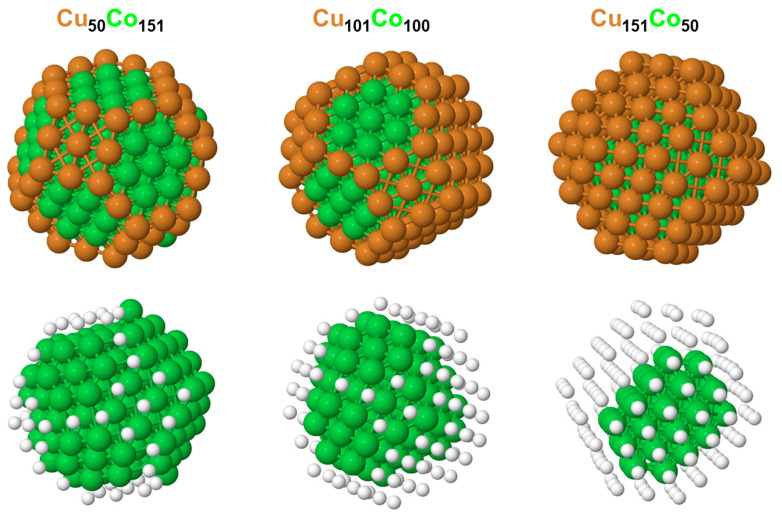
The lowest-energy chemical orderings of truncated-octahedral *fcc* Cu_201-k_Co_k_ NPs with 1:3, 1:1, and 3:1 Cu:Co compositions, as calculated by DFT. The corresponding topologies ($N^{Cu-Co}$, $N_{6}^{Cu}$, $N_{7}^{Cu}$, $N_{8}^{Cu}$, $N_{9}^{Cu}$, and $N^{Co-Co}$) are as follows: Cu_50_Co_151_—(198,17,28,5,0; 680); Cu_101_Co_100_—(267,24,36,6,35; 435); and Cu_151_Co_50_—(208,24,36,6,56; 196). Cu—brown spheres and Co—green spheres. In the bottom row, Cu atoms are represented with small white spheres to better visualize the compact structure of the cobalt component.

The DFT+TOP calculations of the Cu_201-k_Co_k_ (k = 151, 100, 50) NPs with the parameters from Table 1 resulted in the lowest-energy topologies, as depicted in Figure 2. Overall, there was indeed a clear preference of Cu atoms to be in the lower-coordinated outer positions of the NPs, whereas the Co atoms tended to stay inside the NPs and were encapsulated by Cu atoms. This behavior agrees well with the experimental observations and DFT results of CuCo surfaces [14,15,36,37]. The DFT surface segregation energies of a Cu atom impurity at the surface of Co, −480 meV, and of a Co atom impurity at the Cu surface, 330 meV [38], were in the range of the $\varepsilon_{8}^{Cu}\approx -\varepsilon_{8}^{Co}$ and $\varepsilon_{9}^{Cu}\approx -\varepsilon_{9}^{Co}$ values for the NPs with 1:1 and 3:1 Cu:Co contents. Furthermore, the Co atoms form compact Co_151_, Co_100_, and Co_50_ moieties to reduce the number of Cu-Co bonds that are disfavored versus Co-Co bonds ($\varepsilon^{Cu-Co}$ are positive). The number of Co-Co bonds tends to be maximized in these compact cobalt moieties within the constraints of the fixed total number of bonds in the NP and the strong energy gain from Cu atoms located on the surface. Interestingly, such a compact arrangement of Co atoms enclosed by a Cu skin was not noticed in a DFT-based Monte Carlo modeling of CuCo NPs with varying compositions [39].

In more detail, the Cu_50_Co_151_ → Cu_101_Co_100_ → Cu_151_Co_50_ Cu content increase resulted in a gradually higher occupation of the lowest-coordinated surface positions by additionally available Cu atoms, starting with 50 Cu atoms in Cu_50_Co_151_ occupying a part of the surface sites with CN = 6, 7, 8, and absent Cu atoms in the CN = 9 sites in the {111} nanofacets. The Cu_50_Co_151_ NP thus exposes 72 Co atoms that are distributed over all surface sites, but most of these Co atoms are in the {111} nanofacets. An additional 51 Cu atoms in Cu_101_Co_100_ made all of the 101 Cu atoms occupy most of the total 122 surface sites, covering the NP almost completely, except for the 21 CN = 9 sites in the {111} nanofacets, which were still occupied by Co due to the shortage of Cu atoms, none of which were positioned inside the NP. The number of Cu atoms in Cu_151_Co_50_ exceeded the number of 122 available surface sites, leading to a complete coverage of this NP by Cu and putting the remaining 29 Cu atoms inside the NP (but keeping the Co_50_ moiety very compact due to the preference of Co-Co over Co-Cu bond formation (see above)).

Although separation in Cu and Co components has been experimentally observed for bulk Cu-Co alloys [3], the lowest-energy chemical orderings of the 201-atomic CuCo NPs are found to be weakly stable. The instability (due to an unfavorable mixing of Cu and Co atoms) found for bulk Cu-Co alloys [2] is in line with a positive $\varepsilon^{Cu-Co}$ energy (see Table 1). The presence of abundant surfaces on NPs lowers the natural tendency of Cu and Co to separate due to the strongly stabilizing segregation of Cu atoms in surface positions. The miscibility of Cu and Co at the nanoscale (i.e., the stability with respect to the separation into Cu and Co phases) can be evaluated by calculating the excess energy *E*_exc_ [40] of the CuCo NPs versus the homometallic Cu and Co NPs of the same size and structure. For the Cu_201-k_Co_k_ NPs, the excess energy per atom reads as follows: *E*_exc_(Cu_201-k_Co_k_) = {*E*(Cu_201-k_Co_k_) − [(201-k)/201]*E*(Cu_201_) − (k/201)*E*(Co_201_)}/201. The small in magnitude but negative calculated excess energies of −32 meV (Cu_50_Co_151_), −40 meV (Cu_101_Co_100_), and −21 meV (Cu_151_Co_50_) imply that the mixing (alloying) of the copper and cobalt components in these NPs is slightly more favorable than their separation. These *E*_exc_ data on the miscibility of nanoalloy metal components can be compared with the much larger −127 to −163 meV values that are calculated for the 201-atomic alloy NPs of well-miscible Cu and Pt atoms with 1:3, 1:1, and 3:1 Cu:Pt compositions [27].

**Figure 3:**
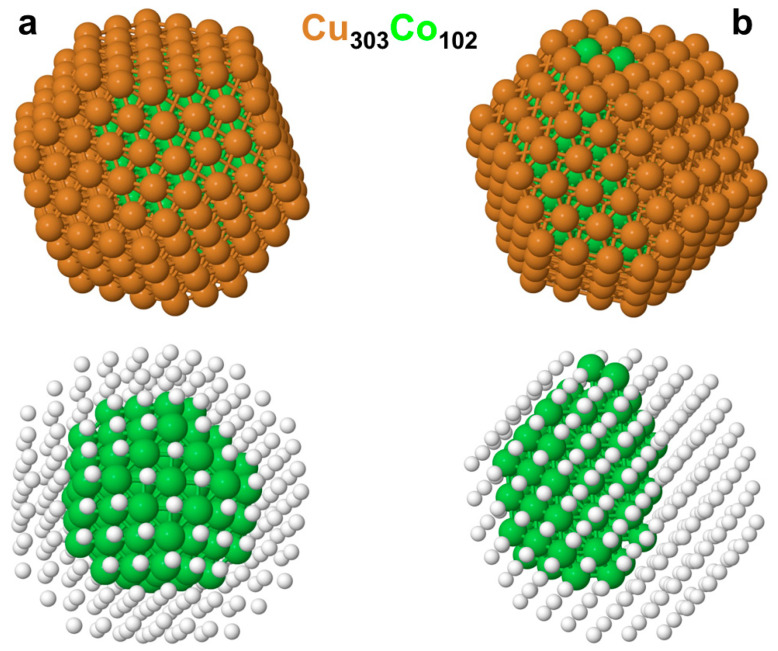
The DFT chemical ordering data of the 405-atomic truncated-octahedral *fcc* Cu_303_Co_102_ NP. (**a**) the lowest-energy homotop with the ${(N}^{Cu-Co},N_{6}^{Cu},N_{7}^{Cu},N_{8}^{Cu}{,N}_{9}^{Cu};N^{Co-Co})$ = (334,24,60,24,96; 445) topology on an entirely Cu skin; (**b**) a homotop exposing two surface Co atoms with the (346,24,60,24,94; 436) topology, which is 1.25 eV higher in *E^DFT^* energy than the homotop depicted in panel (**a**). Cu—brown spheres and Co—green spheres. In the bottom row the Cu are small white spheres to better visualize the Co_102_ moieties.

The homotop of the larger Cu_303_Co_102_ NP with the lowest DFT energy, which was calculated using the TOP parameters from Table 1, is shown in Figure 3a. The (334,24,60,24,96; 445) chemical ordering resulted from the trends discussed above for the 201-atomic NPs. Cu atoms strongly prefer to completely occupy the 204 positions available in the NP skin. The remaining 99 Cu atoms are located inside the NP, enabling them to form a compact Co_102_ moiety embedded in the Cu matrix and touched the 204-atomic Cu skin. The calculated excess energy of −18 meV was, as expected, slightly lower in magnitude than the −21 meV value calculated for the smaller Cu_151_Co_50_ NP with a similar Cu:Co content. Accordingly, the *E*_exc_ again indicated a non-negligible energetic stability of the bare Cu_303_Co_102_ NP versus its separation into copper and cobalt parts. This chemical ordering, which does not expose any Co atoms on the surface, is expected to exhibit a quite low surface reactivity, more like that of the corresponding Cu_405_ NP than of the reactive Co_405_ NP. However, the compact Co_102_ moiety seemed to be rather mobile, with small restructuring, in the Cu_303_ matrix. For instance, positioning the Co_102_ moiety closer to the surface to expose two Co atoms in the skin resulted in the (346,24,60,24,94; 436) ordering shown in Figure 3b, which required only 1.25 eV at the DFT level. A comparison of the latter value with the TOP energy difference of the (b) ordering with the (a) one (due to 12 Cu-Co bonds present in the former) allowed for an estimation of how accurate the TOP expressions are. The TOP energy difference calculated using the energy terms in Table 1 that was determined for the Cu_303_Co_102_ NP equaled 1.17 eV; when using the TOP energy terms determined for the Cu_151_Co_50_ NP, the difference was 1.35 eV. Both these values were quite close to the actual DFT energy difference, 1.25 eV, thus corroborating the finding that energy parameters that are determined for large-enough bimetallic NPs of similar contents of two alloying metals are barely size-dependent and transferrable to the much larger NPs of these metals [22]. On the other hand, the TOP energy differences for the homotops in Figure 3a,b that were calculated using the energy parameters obtained for the Cu_101_Co_100_ and Cu_50_Co_151_ NPs with different Cu:Co compositions were 0.90 eV and 1.87 eV, respectively. These noticeable deviations from the DFT data indicate a stronger dependence of the TOP parameters on the relative content of the alloying metals than on the size of the NPs. This is, again, in full agreement with previous findings [22].

In view of the magnetic character of the CuCo NPs under study, due to the presence of a notable number of magnetic Co atoms in them, it is worth briefly addressing the magnetic properties of the NPs using data of the spin-polarized DFT calculations. As mentioned in the introduction, such data are relevant for preparing magneto-resistive materials based on CuCo NPs. To the best of the authors’ knowledge, the magnetic CuCo NPs considered in this study are among the largest in the literature reporting DFT calculations. For an easier comparison between the different NPs studied in this work, their magnetic moments are presented in the following (in μ_B_ per Co atom, i.e., as the difference of spin-up and spin-down electrons divided by the number of Co atoms in the NP): The parent Cu_201_ and Cu_405_ NPs were non-magnetic, whereas the Co_201_ and Co_405_ NPs featuring the surface-to-bulk-atom ratios decreasing from 1.54 to 1.01 exhibited magnetic moments of 302.04 1.50 μ_B_ and 1.69 μ_B_, respectively, when compared with the 1.7 μ_B_ measured per atom for bulk cobalt [41]. The magnetic moment values for the 201-atomic CuCo NPs were quite close to the latter value—1.66 μ_B_ (Cu_50_Co_151_); 1.63 μ_B_ (Cu_101_Co_100_); and 1.79 μ_B_ (Cu_151_Co_50_). The Cu_303_Co_102_ NPs that are displayed in Figure 3 in both chemical orderings had the magnetic moments of 1.60–1.61 μ_B._ There, the Cu atoms covering the cobalt species reduced the magnetic moment of Co_102_ from the 1.78 μ_B_ calculated for the locally relaxed geometry of the bare Co_102_, which started from its geometries in the Cu_303_Co_102_ NP. According to the calculated Bader atomic charges [42] the electron redistribution between Cu and Co atoms in the studied CuCo NPs was quite small, in line with similar Cu and Co electronegativities. The computed Bader charge ranges q(Cu) = 0.08/−0.11 |e| and q(Co) = 0.18/−0.07 |e| point to an electron density depletion in some of the Co atoms.

### 3.3. Surface Segregation of Co in the Cu_303_Co_102_ Nanoparticle Induced by CO Adsorption

The surface reactivity and catalytic properties of CuCo NPs are critically dependent on the exposure of more active Co atoms at the surface sites. As shown in Section 3.2, if the amount of Cu in a NP is sufficient to form a complete monoatomic Cu skin, enclosing all Co atoms, then this will be the case based on the DFT data corresponding to 0 K. The disorder at room and somewhat higher temperatures can insignificantly alter the preferred topologies of the NPs [22,26,27] and only slightly decrease the propensity of Cu atoms to stay on the surface. Thus, Cu-rich CuCo NPs should barely expose catalytically active surface Co sites in the absence of a reactive environment, or at moderately elevated temperatures. But such NPs completely covered by Cu atoms can be activated by the adsorption-induced surface segregation of Co atoms [14,15,16,17,25,37]. The so-called adsorption preference (AP), defined as the binding energy difference of an adsorbate on pure metals forming the studied bimetallic alloy [14], is crucial for adsorption-induced surface segregation.

**Figure 4:**
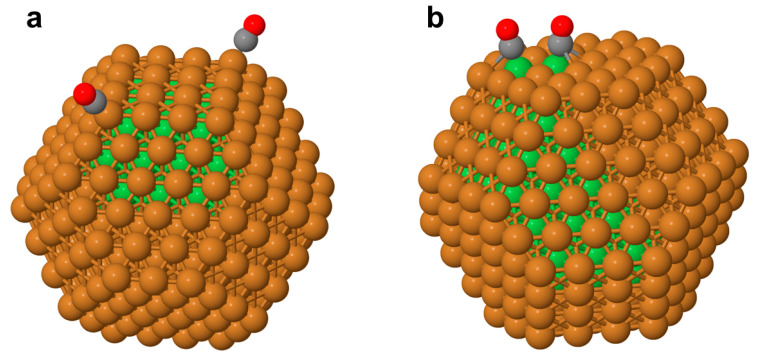
Adsorption of two CO molecules in the most-stable positions of the Cu_303_Co_102_ NP in the chemical orderings displayed in Figure 3: (**a**)—2CO/Cu_303_Co_102_(334,24,60,24,96; 445), where the skin entirely consists of Cu atoms; (**b**)—2CO/Cu_303_Co_102_(346,24,60,24,94; 436), with two surface Co atoms exposed on the surface. Color coding of atoms: Cu—brown, Co—green, C—gray, and O—red.

The ordering change in the Cu_303_Co_102_ NP completely covered by Cu atoms (i.e., the quite inactive one) to the activated ordering with two Co atoms exposed on the surfaces cost as little as 1.25 eV (see caption of Figure 3). DFT calculations of two CO molecules adsorbed on top of the two Co atoms of the Co_405_ NP in the same positions as the surface Co atoms in the ordering shown in Figure 3b resulted in an average CO adsorption energy of 1.57 eV per molecule. The strongest adsorption of two CO molecules on the isomorphic Cu_405_ NP took place on top of two Cu corner atoms. The average DFT adsorption energy in that 2CO/Cu_405_ system was 1.01 eV per CO molecule, resulting in an AP = 0.56 eV per adsorbed CO for the Cu and Co metals in the nanoalloys. Thus, twice this AP value for the strongest possible adsorption of two CO molecules on the Cu_303_Co_102_ NP in the orderings displayed in Figure 3a,b nearly compensated for the higher total energy of the latter. Therefore, the adsorption of two CO molecules on the less-stable structure with two surface-segregated Co atoms was able to convert the structure in a more energetically preferable structure, i.e., it induced Co to segregate on the skins of the CuCo alloy NPs, similar to the findings for the CuCo(111) [14] and other surfaces [15]. Indeed, the Co-unsegregated adsorption system 2CO/Cu_303_Co_102_(334,24,60,24,96; 445) in Figure 4a became 0.05 eV above the 2CO/Cu_303_Co_102_(346,24,60,24,94; 436) one with two surface-segregated Co atoms, as shown in Figure 4b.

In the 2CO/Cu_303_Co_102_(334,24,60,24,96; 445), the average CO adsorption energy on the corner Cu sites, 1.05 eV, was only 0.04 eV higher than on the same sites in 2CO/Cu_405_. The CO adsorption on the {111}-terrace Co sites in 2CO/Cu_303_Co_102_(346,24,60,24,94; 436) resulted in the average CO adsorption energy of 1.70 eV, which was 0.13 eV more than on the same sites in 2CO/Co_405_. The two CO molecules adsorbed on the distant Cu corner sites of Cu_303_Co_102_ hardly affected each other. Yet, the CO molecules adsorbed on the neighboring two Co sites repelled each other by 0.22 eV, which was manifested in a stronger adsorption of single CO molecules, with the energies of 1.84 eV for the Co site near the edge and 1.78 eV for the Co site located more inside the {111} terrace. The adsorption of a single CO molecule separately on each of these sites of Co_405_ resulted in nearly the same adsorption energies, 1.63 eV. Interestingly, during the geometry optimization to calculate a structure with a CO molecule bridging two nearby surface Co atoms, the adsorbate spontaneously moved to an on-top Co position.

C-O stretching vibrational frequencies of the studied adsorption complexes are helpful for characterizing surface sites in CuCo NPs using infrared (IR) spectroscopy. The harmonic vibrational frequency of a free CO molecule calculated using the present DFT setup is 2123 cm^−1^, i.e., 20 cm^−1^ lower than the experimental 2143 cm^−1^ anharmonic frequency. For an easier comparison with experimental IR data, the C-O vibrational frequencies calculated in this work quoted below are adjusted via increasing them by 20 cm^−1^ to partly account for the neglected anharmonicity and the systematic underestimation of the C-O frequency by the employed DFT approach [43].

The adjusted C-O frequencies ν(C-O) in 2CO/Cu_303_Co_102_(334,24,60,24,96; 445) for the adsorption on the corner Cu sites were 2044-2045 cm^−1^, only slightly lower than those for the two CO molecules adsorbed on the same corner sites of the Cu_405_, ν(C-O) = 2047–2048 cm^−1^. The two on-top CO molecules adsorbed on the two Co sites in Cu_303_Co_102_(346,24,60,24,94; 436) revealed the frequencies ν(C-O) = 1944–1947 cm^−1^ significantly down-shifted due to the CO-CO repulsion compared to the single CO molecules that were separately adsorbed on these Co sites and characterized by ν(C-O) = 1955–1956 cm^−1^. The single CO molecules separately adsorbed on these two Co sites of the bare Co_405_ NP featured ν(C-O) = 1948–1952 cm^−1^. Notably, the C-O vibrational frequencies for the on-top CO adsorbates on the Cu and Co sites of the Cu_303_Co_102_(346,24,60,24,94; 436) NP were ca. 50–60 cm^−1^ lower than the experimental IR frequencies reported for the CO adsorption on CuCo alloys formed by Co deposition on the Cu(110) surface [15]. This finding suggests that the adsorption and reactivity properties of surface sites exposed by CuCo alloy NPs may significantly differ from those of corresponding surface alloys.

## 4. Conclusions

A combined *Topological* (TOP) and Density Functional theory (DFT) approach was employed to computationally study the structure and properties of CuCo alloy nanoparticles with different Cu:Co contents and sizes varying from ca. 1.6 to 2.1 nm. First, the TOP parameters were obtained for the 201-atomic NPs (Cu_50_Co_151_, Cu_101_Co_100_, and Cu_151_Co_50_) and the 405-atomic (Cu_303_Co_102_) particles by performing DFT calculations for several dozens of nanoparticles of each kind with different chemical orderings. Energetic parameters of the obtained TOP equations revealed a quite strong propensity of Cu atoms to occupy positions in the skins of the CuCo nanoparticles and that the formation of heterometallic Cu-Co bonds was energetically unfavorable with respect to the formation of homometallic bonds, indicative of the instability of the bulk of such a binary alloy. The TOP equations were shown to be well transferrable from the 1.6 nm-large CuCo particles to bigger particles with similar Cu:Co contents. The most energetically stable chemical orderings of the CuCo nanoparticles under scrutiny were predicted by Monte Carlo simulations using the TOP equations and were confirmed by DFT calculations. The nanoparticles exhibited most of (or whole in the case of higher Cu contents) the outer surface layer formed by the Cu atoms, whereas all the Co atoms formed compact species to maximize the number of Co-Co bonds. These strongly magnetic Co moieties were encapsulated by Cu atoms, and no surface exposure of Co atoms was favored at higher Cu contents, thus hindering the surface reactivity. However, changing a position of the Co species within the Cu matrix to expose some of the Co atoms on the surface requires only a little amount of energy, which could be gained by the interactions of CO molecules showing a notable adsorption preference to the Co surface sites compared to the Cu surface sites. The present DFT calculations quantified such a resurfacing of Co atoms induced by the adsorption of CO molecules. The calculated adsorption energies and vibrational frequencies of the adsorbed CO molecules on various sites of the CuCo nanoparticles should be helpful for monitoring the active sites and surface reactivity of CuCo catalytic nanomaterials using thermo-desorption experiments and infrared spectroscopy.

## Figures and Tables

**Table 1:** The energy parameters *ε* (in meV) defining the topological expressions (Equation (2)) for the Cu_201-k_Co_k_ and Cu_303_Co_102_ truncated-octahedral *fcc* nanoparticles.

Nanoparticle	$\varepsilon^{Cu-Co}$	$\varepsilon_{6}^{Cu}$	$\varepsilon_{7}^{Cu}$	$\varepsilon_{8}^{Cu}$	$\varepsilon_{9}^{Cu}$
Cu_50_Co_151_	29	−953	−948	−829	−762
Cu_101_Co_100_	58	−283	−615	−267	−102
Cu_151_Co_50_	60	−542	−695	−480	−316
Cu_303_Co_102_	55	−734	−385	−503	−255

## Data Availability

The data presented in this study are available on request from the corresponding authors.

## Supplementary Materials

The following supporting information can be downloaded at: https://www.mdpi.com/article/10.3390/nano14151242/s1, S1: Topologies and DFT energies of the Cu_50_Co_151_ (39 homotops), Cu_101_Co_100_ (40 homotops), Cu_151_Co_50_ (33 homotops), and Cu_303_Co_102_ (26 homotops) nanoparticles used to fit the energetic parameters of Equation (2), listed in Table 1, and to calculate the lowest-energy homotops of these nanoparticles; S2, S3: The total energies and magnetic moments of the key structures discussed in this article were calculated by DFT atomic coordinates.
