# Supplementary material for: Chemical Orderings in CuCo Nanoparticles: Topological Modeling Using DFT Calculations
Source: Nanomaterials (Basel). 2024 Jul 24;14(15):1242. doi: 10.3390/nano14151242 (PMC11314349; doi:10.3390/nano14151242)
Supplement: Supplementary file 1 [file nanomaterials-14-01242-s001.zip › nanomaterials-3044431-supplementary.pdf]

Chemical Orderings in CuCo Nanoparticles: Topological Modelling Using DFT Calculations

20.5.2024

Konstantin M. Neyman,[1,2]* Pere Alemany[2]

[1] ICREA (Institució Catalana de Recerca i Estudis Avançats), Pg. Lluís Companys 23, 08010 Barcelona, Spain

[2] Departament de Ciència de Materials i Química Física and Institut de Quimica Teòrica i Computacional (IQTCUB), Universitat de Barcelona, c/ Martí i Franquès 1, 08028 Barcelona, Spain

Supplementary Materials

S1. Energies and Topologies of the homotops used to fit the TOP parameters given in Table 1 and to calculate the lowest-energy homotops

In the following, the ***Topology*** is (N^{Cu-Co},N_6^{Cu},N_7^{Cu},N_8^{Cu},N_9^{Cu}; N^{Co-Co}), where N^{Cu-Co} and N^{Co-Co} are the numbers of Cu–Co and Co–Co bonds (Cu–Co and Co–Co nearest-neighbor atom pairs) and N_6^{Cu}, N_7^{Cu}, N_8^{Cu}, and N_9^{Cu} are the numbers of Cu atoms at corners (vertexes), edges, {001} and {111} nanofacets with the coordination numbers 6, 7, 8, and 9, respectively. Comparison of the listed E^{TOP} values with DFT E^{PBE} ones illustrate the quality of the fitting.

$Cu_{50}Co_{151}$

E^{TOP}(eV) = -1099.910 + 0.029N^{Cu-Co} - 0.953N_6^{Cu} - 0.948N_7^{Cu} - 0.829N_8^{Cu} - 0.762N_9^{Cu}

* Correspondence: K.M.N. - konstantin.neyman@icrea.cat

№	Label	E^{PBE} (eV)	Topology	E^{TOP} (eV)
1	3	-1136.4235	(230,13,21,3,11; 647)	
2	4	-1136.8063	(227,14,21,3,10; 650)	
3	5	-1137.6201	(218,14,21,3,11; 656)	
4	7	-1137.7353	(229,15,22,3,9; 653)	
5	24	-1139.7402	(212,17,23,5,5; 668)	
6	25	-1139.7851	(210,17,23,5,5; 669)	
7	26	-1139.8720	(209,16,24,5,5; 669)	
8	27	-1139.9714	(214,17,24,5,4; 668)	
9	28	-1139.9282	(215,16,25,5,4; 667)	
10	29	-1139.8094	(216,15,26,5,4; 666)	
11	30	-1140.1059	(213,16,26,5,3; 669)	
12	31	-1140.2033	(215,16,27,5,2; 669)	
13	32	-1140.1585	(213,16,27,5,2; 670)	
14	33	-1140.4246	(210,17,26,5,2; 672)	
15	34	-1140.4316	(211,17,26,6,1; 672)	
16	35	-1140.6056	(211,17,27,6,0; 673)	
17	36	-1140.6505	(209,17,27,6,0; 674)	
18	37	-1140.7665	(207,18,27,5,0; 676)	
19	38	-1140.8106	(204,19,26,5,0; 678)	-1140.894
20	39	-1141.0208	(203,19,27,4,0; 679)	-1141.042
21	40	-1140.9297	(204,18,28,4,0; 678)	-1141.008
22	41	-1140.9459	(199,18,27,5,0; 680)	-1141.034
23	42	-1141.1259	(197,18,27,5,0; 681)	-1141.092
24	43	-1141.1677	(198,17,28,5,0; 680)	-1141.058
25	110	-1140.3711	(219,14,30,5,1; 667)	
26	111	-1140.5310	(220,15,30,5,0; 668)	
27	112	-1140.3189	(225,14,31,5,0; 665)	
28	113	-1140.2226	(228,13,32,5,0; 663)	
29	114	-1139.9556	(233,12,33,5,0; 660)	
30	115	-1140.1798	(226,13,32,5,0; 664)	
31	116	-1140.2476	(226,14,32,4,0; 665)	
32	117	-1140.4430	(224,14,32,4,0; 666)	
33	118	-1140.5275	(224,15,32,3,0; 667)	
34	119	-1140.7201	(224,16,32,2,0; 668)	
35	120	-1140.8615	(224,17,32,1,0; 669)	
36	121	-1140.9624	(224,18,32,0,0; 670)	-1140.904
37	122	-1141.0037	(217,18,31,1,0; 673)	-1140.988
38	205	-1140.9935	(203,16,29,5,0; 677)	-1140.908
39	206	-1141.0961	(203,17,29,4,0; 678)	-1141.032

$Cu_{101}Co_{100}$

$\boldsymbol{E^{TOP}}$(eV) = -965.307 + 0.058$\boldsymbol{N^{Cu-Co}}$ - 0.283$\boldsymbol{N_6^{Cu}}$ - 0.615$\boldsymbol{N_7^{Cu}}$ - 0.267$\boldsymbol{N_8^{Cu}}$ – 0.102$\boldsymbol{N_9^{Cu}}$

№	Label	E^{PBE} (eV)	Topology	E^{TOP} (eV)
1	4	-963.49151	(412,19,23,5,25; 298)	
2	8	-963.80358	(417,21,23,4,26; 301)	
3	9	-963.07280	(428,21,23,4,25; 294)	
4	13	-964.05587	(431,21,25,4,26; 299)	
5	15	-964.73525	(426,21,26,3,28; 305)	
6	19	-964.00240	(448,21,28,1,28; 295)	
7	21	-964.76594	(445,21,29,1,28; 299)	
8	25	-964.72982	(465,21,29,2,28; 291)	
9	27	-964.82063	(470,21,30,2,26; 288)	
10	31	-965.85704	(464,20,32,3,26; 295)	
11	33	-966.11617	(467,20,32,3,27; 295)	
12	37	-967.45650	(458,22,32,5,24; 305)	
13	39	-967.24897	(461,22,33,4,24; 304)	
14	43	-968.68443	(451,22,34,4,25; 313)	
15	45	-968.79435	(448,22,34,4,26; 316)	
16	49	-968.73298	(460,22,35,3,27; 312)	
17	51	-968.67406	(465,23,35,3,26; 311)	
18	53	-969.02592	(460,24,35,3,25; 315)	
19	55	-970.13043	(446,24,35,3,27; 325)	
20	57	-970.62907	(445,24,35,3,28; 327)	
21	59	-970.96949	(442,24,35,3,29; 330)	
22	61	-971.03397	(445,24,35,4,28; 329)	
23	63	-971.85676	(437,24,36,5,27; 336)	
24	65	-972.40521	(430,24,36,5,28; 341)	
25	67	-972.02884	(431,24,36,6,27; 341)	
26	69	-972.67237	(422,24,36,6,28; 347)	-973.230
27	71	-972.45846	(425,24,36,5,29; 345)	
28	73	-972.26343	(427,24,36,5,29; 344)	
29	75	-971.89653	(430,24,36,2,32; 342)	
30	77	-971.26099	(432,24,36,2,32; 340)	
31	165	-981.55095	(277,24,36,5,33; 425)	-981.874
32	166	-981.52077	(275,24,36,5,33; 426)	-981.990
33	167	-981.86866	(274,24,36,5,34; 428)	-982.150
34	168	-982.37238	(274,24,36,6,34; 430)	-982.417
35	169	-982.25253	(273,24,36,5,35; 430)	-982.310
36	170	-982.24972	(270,24,36,4,36; 431)	-982.493
37	171	-982.15570	(267,24,36,4,35; 431)	-982.391
38	172	-982.52439	(269,24,36,5,35; 432)	-982.542
39	173	-982.75519	(270,24,36,5,36; 433)	-982.760
40	174	-983.11798	(267,24,36,6,35; 435)	-982.925

$Cu_{151}Co_{50}$

$$\boldsymbol{E^{TOP}}(\text{eV}) = -776.939 + 0.060\boldsymbol{N^{Cu-Co}} - 0.542\boldsymbol{N_6^{Cu}} - 0.695\boldsymbol{N_7^{Cu}} - 0.480\boldsymbol{N_8^{Cu}} - 0.316\boldsymbol{N_9^{Cu}}$$

№	Label	E^{PBE} (eV)	Topology	E^{TOP} (eV)
1	1	-821.61735	(222,23,36,6,56; 186)	
2	2	-821.79242	(223,24,36,6,55; 187)	
3	3	-822.16452	(222,24,36,6,56; 189)	
4	4	-822.19145	(220,24,36,6,56; 190)	
5	5	-822.48113	(218,24,36,6,56; 191)	
6	6	-822.56987	(216,24,36,6,56; 192)	
7	7	-822.59926	(214,24,36,6,56; 193)	
8	8	-822.80796	(212,24,36,6,56; 194)	
9	9	-822.76773	(210,24,36,6,56; 195)	
10	10	-822.96474	(208,24,36,6,56; 196)	
11	101	-821.59662	(220,23,36,6,56; 187)	
12	102	-987.35910	(223,24,36,6,56; 187)	
13	103	-821.86552	(218,23,36,6,56; 188)	
14	104	-821.95643	(216,24,36,5,56; 190)	
15	105	-822.24143	(212,24,36,5,56; 192)	
16	106	-822.43183	(213,24,36,6,55; 192)	
17	107	-822.52184	(210,24,36,5,56; 193)	
18	108	-822.48687	(208,24,36,5,56; 194)	
19	200	-821.05252	(221,23,35,6,56; 184)	
20	201	-819.87998	(228,23,34,6,56; 178)	
21	202	-820.14724	(227,24,34,6,55; 180)	
22	203	-819.93014	(224,24,34,6,54; 180)	
23	204	-820.02745	(225,24,35,6,52; 179)	-820.084
24	317	-821.79010	(234,24,36,6,56; 183)	-821.503
25	318	-821.84012	(232,24,36,6,56; 184)	
26	319	-822.03713	(226,24,36,6,56; 187)	
27	320	-822.13856	(224,24,36,6,56; 188)	
28	321	-822.14273	(222,24,36,6,56; 189)	-822.223
29	322	-822.49322	(218,24,36,6,56; 191)	
30	323	-822.58470	(216,24,36,6,56; 192)	
31	324	-822.72931	(214,24,36,6,56; 193)	
32	325	-822.73988	(212,24,36,6,56; 194)	-822.823
33	326	-823.00462	(208,24,36,6,56; 196)	-823.063

Contributions to the stability of № 33: -823.063 + 776.93 = -46.124 eV (-0.2295 eV/atom)

N^{Cu-Co}	12.480/-46.124 = -27.06%	Cu-Co bonds (“bulk” contribution)
N_6^{Cu}	-13.008/-46.124 = 28.20%	CN6 sites (corners)
N_7^{Cu}	-25.020/-46.124 = 54.25%	CN7 sites (edges)
N_8^{Cu}	-2.880/-46.124 = 6.24%	(001) terrace sites
N_9^{Cu}	-17.696/-46.124 = 38.37%	(111) terrace sites

$Cu_{303}Co_{102}$

$\boldsymbol{E^{TOP}}$(eV) = -1643.740 + 0.055$\boldsymbol{N^{Cu-Co}}$ - 0.734$\boldsymbol{N_6^{Cu}}$ - 0.385$\boldsymbol{N_7^{Cu}}$ - 0.503$\boldsymbol{N_8^{Cu}}$ – 0.255$\boldsymbol{N_9^{Cu}}$

**№**	**Label**	***E^PBE^* (eV)**	**Topology**	***E^TOP^* (eV)**
1	35	-1680.8593	(446,18,52,20,72; 307)	
2	42	-1683.1188	(464,20,56,20,72; 314)	
3	55	-1686.6530	(449,22,56,21,75; 334)	
4	78	-1689.9634	(468,23,60,23,80; 349)	
5	96	-1693.5600	(429,23,60,23,85; 376)	
6	120	-1697.1886	(397,24,60,23,91; 404)	
7	123	-1697.8189	(394,24,60,23,92; 407)	
8	124	-1697.9897	(399,24,60,23,93; 406)	
9	128	-1698.7920	(386,24,60,23,94; 414)	
10	129	-1699.1285	(387,24,60,23,95; 415)	
11	132	-1699.8029	(383,24,60,24,95; 419)	
12	134	-1700.4525	(373,24,60,24,95; 424)	
13	137	-1701.5142	(360,24,60,24,96; 432)	
14	141	-1702.0734	(348,24,60,24,96; 438)	
15	145	-1702.1794	(340,24,60,24,96; 442)	
16	298	-1700.8749	(352,24,60,24,94; 433)	-1701.138
17	299	-1701.1609	(348,24,60,24,94; 435)	-1701.358
18	300	-1701.3028	(346,24,60,24,94; 436)	-1701.468
19	301	-1701.5924	(347,24,60,24,95; 437)	-1701.668
20	302	-1701.9832	(348,24,60,24,96; 438)	-1701.868
21	303	-1701.9349	(346,24,60,24,96; 439)	
22	304	-1702.0386	(344,24,60,24,96; 440)	-1702.088
23	305	-1702.2353	(342,24,60,24,96; 441)	
24	306	-1702.2555	(340,24,60,24,96; 442)	-1702.308
25	307	-1702.5337	(336,24,60,24,96; 444)	-1702.528
26	2.18	-1702.5481	(334,24,60,24,96; 445)	-1702.638

Contributions to the stability of № 26: -1702.638 + 1643.740 = -58.898 eV (-0.1454 eV/atom)

N^{Cu-Co}	18.370/-58.898 = -31.19%	Cu-Co bonds (“bulk” contribution)
N_6^{Cu}	-17.616/-58.898 = 29.91%	CN6 sites (corners)
N_7^{Cu}	-23.100/-58.898 = 39.22%	CN7 sites (edges)
N_8^{Cu}	-12.072/-58.898 = 20.50%	(001) terrace sites
N_9^{Cu}	-24.480/-58.898 = 41.56%	(111) terrace sites

S2. Calculated atomic coordinates, total energies, and magnetic moments of key structures of 201-atomic bare metal nanoparticles

The DFT (PBE) data calculated by VASP code are listed in this section as CONTCAR files obtained with the cutoff energy for the plane-wave functions of 273.2 eV.

$Cu_{50}Co_{151}$

Topology (198,17,28,5,0; 680), E^{PBE} = -1141.1677 eV, magnetic moment = 250.28 μ_B $Cu_{50}Co_{151}$g43

```
Cu   Co   Cu50Co151
   1.00000000000000
    25.0000000000000000    0.0000000000000000    0.0000000000000000
     0.0000000000000000   25.0000000000000000    0.0000000000000000
     0.0000000000000000    0.0000000000000000   25.0000000000000000
   Cu   Co
    50   151
Direct
  0.4292997933097188  0.5720855822545076  0.2190809416007108
  0.5710734477925292  0.5719820975106287  0.2191420063406344
  0.3597058779463777  0.5010923694001606  0.2206636204869079
  0.6405012520579350  0.5007808273461893  0.2207454727183902
  0.4292094410686772  0.4295685342259173  0.2186707039062386
  0.4999177908525381  0.3595959333868559  0.2204973330311831
  0.2207000431356244  0.5010819811705832  0.3593686658993130
  0.7793817259400680  0.5006294476551832  0.3595908305647603
  0.2174139201673196  0.5007777972645082  0.4998631178316031
  0.2203035959376702  0.3597513643831167  0.4997668193692223
  0.2205986474501214  0.5008915014763909  0.6403977183624620
  0.5708359279018831  0.5716865243108312  0.7810266326670976
  0.4290298361971296  0.5718141597280547  0.7809268921532324
  0.3594799475175967  0.5007694520292425  0.7792156210271599
  0.2883768586063222  0.5005555347795769  0.2881715478503237
  0.7115751264296042  0.5002832841690509  0.2884223117615219
  0.2191458477444433  0.5719808478173258  0.4289809816305971
  0.2186225147021268  0.4295458354593318  0.4288836758115463
  0.2879957879807158  0.2888934756763777  0.4997434807377161
  0.2190747649173283  0.5718901528567399  0.5708471311618554
  0.2185344039823419  0.4294700002206219  0.5707678952572945
  0.2881941303574658  0.5002679714917538  0.7116591979798796
  0.5001776269006237  0.5008357208461893  0.2175121045733762
  0.5708921075908751  0.4293412647660511  0.2187845091440361
  0.4997755261757617  0.2211762232621096  0.3592557246723002
  0.7793093885946191  0.6420140755601657  0.5002155510873570
  0.7824585314421324  0.5003728670186680  0.5001443507732130
  0.7796507135142893  0.3592128980417443  0.5000424395602620
  0.3590602714085590  0.2213613816540341  0.4997504811389403
  0.4997207170561620  0.2177864157745138  0.4998095660624863
  0.6405948716812064  0.2210489138710805  0.4998810058892274
  0.7792685474909250  0.5004298290450531  0.6406156045246557
  0.4996618888819463  0.2210377474158474  0.6404572877514179
  0.4998782535499204  0.5005072834263189  0.7825046672515213
  0.6402667418887774  0.5004437023935564  0.7793337744613591
  0.4289012106430597  0.4292028134369746  0.7811478438797667
  0.5706465661134686  0.4290509300286336  0.7812045641474527
  0.4996348788938969  0.3593112315943035  0.7793809870251217
  0.4998999916668501  0.2886877543147596  0.2881629972604262
  0.7805002821162029  0.5718116720438169  0.4292823967924589
  0.7812952051844432  0.4290236702109189  0.4290425344061292
  0.5709558446556896  0.2194301993505818  0.4288132593254124
  0.4286378123661354  0.2195555299411144  0.4287491461316878
  0.7117193284604628  0.2884808879337227  0.4999651195360806
  0.7804377674882739  0.5717465133322615  0.5710492803119687
  0.7812018125315834  0.4289060129784183  0.5711229901295448
  0.4286061275751373  0.2194799042836646  0.5708686234082161
  0.5708672253729279  0.2193583126108468  0.5709116331858841
  0.7113992549314226  0.4999717158039851  0.7117330539430040
```

0.4996954284125435	0.2884830109995530	0.7116146233951322
0.5003155440585679	0.6432549225853584	0.2283792609034706
0.5001772887788776	0.7763470714377625	0.3631264371144418
0.3631157718098255	0.7763207486412014	0.5000986726755564
0.2283363052622560	0.6430474072490366	0.4999559434766071
0.5000680778359766	0.7761499234866142	0.6372292305740493
0.4307440823211761	0.7087535517234886	0.6384284193918482
0.3612083382207726	0.6396059547448000	0.6387760612976418
0.2923699157134279	0.5715403786219481	0.6393760563870586
0.5000771179739297	0.6429286518219144	0.7718152784145329
0.4999102172975355	0.7091570082695124	0.2950870895971436
0.4319989990307243	0.7772919913999710	0.4318933033748034
0.2915205044420329	0.6406081565690230	0.4294860676314117
0.2950411581973569	0.7090219244818275	0.5000268082899859
0.5679696112841265	0.7772766622081685	0.5682422334054409
0.4319076639948682	0.7771675981846573	0.5684287399711132
0.3616577698613241	0.7088136291618602	0.5692416649647960
0.2914563021105321	0.6405074471330238	0.5704787376583786
0.4997350958001933	0.7088963315655444	0.7051870398172695
0.4295562540560982	0.6403267406321743	0.7086019285502800
0.3605177709621628	0.5715194220710116	0.7075137867532270
0.5693953014456097	0.7088541656111949	0.3616740371445750
0.6392337167508700	0.6396760563507626	0.3614678837748950
0.7077868143054200	0.5711344815513287	0.3605418010347172
0.7081580907174968	0.4287984090868164	0.3602861237382651
0.6388123846051977	0.3616449949451096	0.3612423677429140
0.5713286166092157	0.2924366497493670	0.3601350207974988
0.6372949751549642	0.7758151521642956	0.5002090013910958
0.5692844863622487	0.7086538084859684	0.6386605847110506
0.6391015468227712	0.6394781669805826	0.6388088226164387
0.7076606164118868	0.5709513090841826	0.6397225568245584
0.7080211389786412	0.4286150766923632	0.6398116062058089
0.6386702867667717	0.3614694021986904	0.6387128688849466
0.5712135984647285	0.2922726624360998	0.6397033046055653
0.5705757512690034	0.6406249059164875	0.2914774996647267
0.6395592140329455	0.5717974945327468	0.2924801325017006
0.6399251716848048	0.4288982980909448	0.2917005017605142
0.5714024779909208	0.3606490095837945	0.2919260840068097
0.5680024262572381	0.7773823478636698	0.4321878772714360
0.6378384346017861	0.7087858607629806	0.4310725371201809
0.7073594890810274	0.6413081178779009	0.4294434161675380
0.7081577290459731	0.3604969415682662	0.4286283245870992
0.6397650117940796	0.2923830955495906	0.4284431117648305
0.7056754015140294	0.7085164684545066	0.5002013665343442
0.6377727388660159	0.7086915686026445	0.5692934143151455
0.7072889904374851	0.6412194967414436	0.5709002229861513
0.7080797912005297	0.3603998885615011	0.5713888227167624
0.6397003099738399	0.2922848055449038	0.5714383615237659
0.5703868158372574	0.6403605257213452	0.7087639407244635
0.6393798517448460	0.5715533206970316	0.7077182553670336
0.6397172434248269	0.4286480730950903	0.7083282738164243
0.5712041174787835	0.3604187008698350	0.7079882955140359
0.4308764134912901	0.7089483288358253	0.3617844873489499
0.3613413961829559	0.6397941717454957	0.3612542915556328
0.2925051317044284	0.5717019518971016	0.3605242594339167
0.2916363471268931	0.4291671838286477	0.3598918283751455
0.4297483411184783	0.6405784956330542	0.2915013051125712
0.3607192809527969	0.5717607834754752	0.2924358137012478
0.3601810433589142	0.4291989253099028	0.2915249523925155
0.3617253712928359	0.7089212659033992	0.4308818861156063
0.5000877597910292	0.6402750558407190	0.3593579505568136
0.4302795522288135	0.5700227168249198	0.3592289972042730
0.5702102384337908	0.5699720242995756	0.3592193308938749
0.3596588529366156	0.5000887635291017	0.3594728277089156
0.5001899829314834	0.4992050586298814	0.3582770827047014
0.6404756935229092	0.4999555004652681	0.3597160450750538
0.4300287860761458	0.4304037265165455	0.3588376543972022
0.5700403628125829	0.4302198920185297	0.3588194051588774
0.3612234088797704	0.3619397290815990	0.3610875547047082
0.4999574455488244	0.3599529405863802	0.3593799801659388

0.4284207467696375	0.2925840355162301	0.3600586332834899
0.5000732438597245	0.7772416815466061	0.5002187737192687
0.4295709671494790	0.7098700680131438	0.5000997626903108
0.5703402747476763	0.7094409101614809	0.5001620230450119
0.3594235981177817	0.6402499334425440	0.5000128533184151
0.4999820812112154	0.6369739372127839	0.5000808545502254
0.6403195702866319	0.6403939861625175	0.5001432115711363
0.2893998792499648	0.5721937991904601	0.4999394329912468
0.4303060494027527	0.5683156121911945	0.5000056345596920
0.5697898108188694	0.5684639005409894	0.5000662608600535
0.7104739006236349	0.5723049154583609	0.5001302536582537
0.3581266970743870	0.4991219867818483	0.4999347142493938
0.5001040962003374	0.4990893116877383	0.4999847464023144
0.6425096924203860	0.4996095818025006	0.5000583952337417
0.2895476799469873	0.4290449687430428	0.4998748829715313
0.4294842754933449	0.4299387351526913	0.4999196063789835
0.5703672186733992	0.4297468353817379	0.4999869161843051
0.7104067539207457	0.4284243077440307	0.5000431815492054
0.3594871026085523	0.3600276162301665	0.4998566085242225
0.4999352117471345	0.3585703451392385	0.4999056165657279
0.6402404203408386	0.3596097130968315	0.4999814391807094
0.4287743537377126	0.2903444202773474	0.4998383080212438
0.5709380620689835	0.2900364494875637	0.4998969838889765
0.4999618698516388	0.6400854570787836	0.6408352300089445
0.4301441539841407	0.5698409950076367	0.6407933402103245
0.5700820909686770	0.5697912428948932	0.6409173835840462
0.3595158297157844	0.4999226579116644	0.6403874444372689
0.5000519536785512	0.4990205763268850	0.6417180647911005
0.6403339402455727	0.4997803357418501	0.6404143261868197
0.2915039357706453	0.4289776817708697	0.6398231978813974
0.4299025011850492	0.4302317193003708	0.6410035063153332
0.5699048991263338	0.4300554192653188	0.6411463725511767
0.3610910185151574	0.3617447599772088	0.6386132587033183
0.4998488523625478	0.3597731870751615	0.6404304917106955
0.4283172337309331	0.2924274714612676	0.6396260109046791
0.5001227960534206	0.5722202832377125	0.2894317746668590
0.4289894046160188	0.5003904595602343	0.2898739457265685
0.5713481525777252	0.5001985077310580	0.2899099238572357
0.5000210744654867	0.4291612527375961	0.2896180380027854
0.4285673971720455	0.3608560363931797	0.2917991187857542
0.5000979670164092	0.7097447883680366	0.4295385441337619
0.4298364952971975	0.6388682398593492	0.4297982568206878
0.5703166613132638	0.6389206297590573	0.4298157765494932
0.3591709642251865	0.5699674772259191	0.4301230877051180
0.5001079160594428	0.5683027558759814	0.4303331377265543
0.6410129537444319	0.5700655905642213	0.4300865370714182
0.2898734967404277	0.5001714345854947	0.4286773214088235
0.4295997114889960	0.4996404214422130	0.4294976185606147
0.5706791050814303	0.4996212764583268	0.4296596557531108
0.7104206133864615	0.4997903814942839	0.4290301333741028
0.3588390910701041	0.4303471478694482	0.4298435246470981
0.5000863714345503	0.4299113106317602	0.4295237841585537
0.6410432012143489	0.4300747897043758	0.4301746498375392
0.2917553047107952	0.3609385000502248	0.4283757686483408
0.4298982449189633	0.3595348639841284	0.4298922575122954
0.5699158017365529	0.3594101688693424	0.4300058383395684
0.3599650764206135	0.2926638305148299	0.4283640786264940
0.4998056577997869	0.2903056810610350	0.4287219301120015
0.5000434566496097	0.7096384336387708	0.5707516735327639
0.4297740670957941	0.6387778999003694	0.5703051049701744
0.5702484467488820	0.6388250455224290	0.5704110470715414
0.3591052352011768	0.5698738309764916	0.5698253782030316
0.5000383568742020	0.5682127670926121	0.5697480703113120
0.6409531130585064	0.5699716430851661	0.5701062245192630
0.2897992052228252	0.5000879164806830	0.5711161281847469
0.4295224967737326	0.4995415263712339	0.5704311795450666
0.5706221514856887	0.4995311769181268	0.5703959059550427
0.7103468335302028	0.4997014976176288	0.5711570242629688
0.3587684093869857	0.4302642800932356	0.5699476484446371
0.5000262982698523	0.4298123716003631	0.5703800232337527

0.6409848894187212 0.4299943936205868 0.5698591236464452
0.2916727417473798 0.3608336576132538 0.5712869133164723
0.4298460048645960 0.3594490533535705 0.5698631894183763
0.5698485541569170 0.3593052816112906 0.5698758009491844
0.3599032790207281 0.2925738552563527 0.5712430849956646
0.4997445534332741 0.2902055315757875 0.5710068514211600
0.4999380333485741 0.5719533672980236 0.7106769501792324
0.4287832859843156 0.5001362679804132 0.7100487790032110
0.5711358893450962 0.4999640040445560 0.7101599862352551
0.3599847756598024 0.4289489327445563 0.7082421527643940
0.4998326043505038 0.4289049176012349 0.7102986018855937
0.4284051526067615 0.3606195169188928 0.7079611866549375

$Cu_{101}Co_{100}$

Topology (267,24,36,6,35; 435), E^{PBE} = -983.11798 eV, magnetic moment = 163.26 μ_B $Cu_{101}Co_{100}$g174

```
Cu   Co
   1.00000000000000
    25.0000000000000000    0.0000000000000000    0.0000000000000000
     0.0000000000000000   25.0000000000000000    0.0000000000000000
     0.0000000000000000    0.0000000000000000   25.0000000000000000
   Cu   Co
   101   100
Direct
  0.4976810958754901  0.6400700098031086  0.2194888519953201
  0.4279323097805714  0.5707767779042904  0.2187260510110375
  0.5687396097988897  0.5699985265399274  0.2168388586421424
  0.3590066693077510  0.4999511850944081  0.2211950151807761
  0.6385602765561981  0.4997247691412519  0.2180256898208262
  0.4278608126478975  0.4291273402092262  0.2187684047224929
  0.4974391876293781  0.3596799416073810  0.2196790117449457
  0.4978972385441844  0.7795412558969510  0.3593815130177835
  0.2201929198592688  0.4996635890596280  0.3595553725332831
  0.7797193195180128  0.4996268896321708  0.3598241524519484
  0.3591745389608341  0.7791426301063804  0.4982951344505805
  0.2187771269841622  0.6396699760047146  0.4983973332662235
  0.2163673505597420  0.4997403807523843  0.4993860368642794
  0.2200497991166376  0.3598695850273685  0.4991328089811139
  0.4988234892972149  0.7805246452876421  0.6394936918882064
  0.4266231446887971  0.7130137326790912  0.6424415673381790
  0.3578036943557812  0.6411107403784088  0.6411541527264385
  0.2857580747018284  0.5719148001592410  0.6420933602191558
  0.2185397628136775  0.4985657367554239  0.6392941887157144
  0.4986209880672353  0.6390469439512755  0.7807788354232563
  0.5691089092802918  0.5693590664120336  0.7834236787327378
  0.4286675421174101  0.5691374383925687  0.7817492191195894
  0.3587819351716470  0.4981218420575151  0.7790195731414322
  0.4980089636198568  0.7109208960052547  0.2884346996874299
  0.2878732140856454  0.4998740801193871  0.2885548494265754
  0.7103243737871316  0.4996911039824894  0.2880188964857440
  0.4279912490092572  0.7810373640206474  0.4282194557569156
  0.2177320853521705  0.5707302705254937  0.4282620884740798
  0.2187399617384062  0.4292636705425922  0.4288593462758024
  0.2881035511340480  0.7105898628146814  0.4984604673338441
  0.2876410703274529  0.2888308675629003  0.4993204985594665
  0.5693654766570766  0.7831128681977616  0.5697767328400301
  0.4289675942014768  0.7816813669543280  0.5694832954142364
  0.3565007153921116  0.7125674277704575  0.5718627664612259
  0.2858817785990383  0.6422291225065995  0.5718498006902932
  0.2170182279581685  0.5695447624041967  0.5692977950370870
  0.2175217987313097  0.4285003403514868  0.5702978554847328
  0.4989822776163998  0.7109260224951707  0.7112431253746422
  0.4265196817536768  0.6422082314102163  0.7131946170206167
  0.3562639567618561  0.5717574292905161  0.7125362139033231
  0.2877471811142999  0.4984581728212175  0.7103775329584539
  0.4985303123108845  0.4998327397289166  0.2161935703567323
  0.5686501024167148  0.4295929072453544  0.2170152979124005
  0.5717489986817943  0.7130356447149137  0.3566314335812028
```

0.6415222155394785	0.6418731723826678	0.3575634296692323
0.7130088225220621	0.5726804262101521	0.3564274358940613
0.7127018316121841	0.4269339386904805	0.3565208737166408
0.6410424528532529	0.3578823401909327	0.3578174685754821
0.5713590074260885	0.2865315847931192	0.3567740927221424
0.4973530276041106	0.2198474095332055	0.3594184034346136
0.4989619437285254	0.7840590421594955	0.4990787738039542
0.6391616223970128	0.7809714767741853	0.4994529800694276
0.7801481041223517	0.6396892749944576	0.4999436006579440
0.7842419233074243	0.4996787980061894	0.5000573963944606
0.7796402091736401	0.3597510301889641	0.4997921805193915
0.3587265053594455	0.2214395320644894	0.4994173544838665
0.4982957378405442	0.2162862267885237	0.4994695551124692
0.6383208163893515	0.2180607475393514	0.4995621979624353
0.5723747981076418	0.7138957617126506	0.6433553721635612
0.6417570353484863	0.6422375743570067	0.6426196841331975
0.7132694270240025	0.5727696272524838	0.6436781178727949
0.7799961400675122	0.4994877842836075	0.6401644181171166
0.7130063092584200	0.4266371693353489	0.6431670283772383
0.6412655342966315	0.3572991332552647	0.6419430169155049
0.5713244141957924	0.2859185131401875	0.6425245356006433
0.4972801227709769	0.2194376316204787	0.6397289990724273
0.4986262944240874	0.4987389603554680	0.7841567289799233
0.6387924057804112	0.4989748462814665	0.7812390899244770
0.4275995913459862	0.4280410705828136	0.7809643465766180
0.5688817347004460	0.4287827720921354	0.7820230423930911
0.4974583661365878	0.3591315092166389	0.7794700899379859
0.5716535608858628	0.6426628042150292	0.2860778560135266
0.6422449096430063	0.5726427501266906	0.2854951409219005
0.6419623028067800	0.4270796570891373	0.2856902187879378
0.5714106380978191	0.3569709715151009	0.2863579471105930
0.4975847060126666	0.2890442365982656	0.2888349753999699
0.5693162957542787	0.7819483871265933	0.4291795840455868
0.6425169128123619	0.7135668172764013	0.4268242425188710
0.7132541047174472	0.6428737028922246	0.4270098669607804
0.7822356749256170	0.5700141706449788	0.4295922157577116
0.7817667222346962	0.4294117832828900	0.4295163241825787
0.7126648190211738	0.3564914197941069	0.4269919132924964
0.6418693389353531	0.2857475986423347	0.4269831903344126
0.5684920644484402	0.2171468465795541	0.4293388869556338
0.4276961891886006	0.2190010905653106	0.4287575002402371
0.7108379712904418	0.7113910243985886	0.4998477093216478
0.7101845378090509	0.2879596742659956	0.4996976432506781
0.6426898844625165	0.7141556711471041	0.5729036876842091
0.7133586034643811	0.6432636217976359	0.5731962374720948
0.7822526375122004	0.5700815459434340	0.5705235781120260
0.7820630740232708	0.4292563588738083	0.5704232873982974
0.7128088408826699	0.3562188046753411	0.5727943025357046
0.6419917200539816	0.2853673897420086	0.5725789416519689
0.4275919654950537	0.2187867703039790	0.5703348170495897
0.5684110556124512	0.2167824154122148	0.5697623538420313
0.5722799313217186	0.6429554144738963	0.7142606860064076
0.6425045184206862	0.5724969202139355	0.7145238143522530
0.7105913393974732	0.4993669861539222	0.7117393719739102
0.6421967478819948	0.4264416019460613	0.7137697902625689
0.5713773072603170	0.3563380503752937	0.7130158901830738
0.4976040026242247	0.2882694469340882	0.7107365847616332
0.4284656396231428	0.7071792608973160	0.3606605883636056
0.3619996145708202	0.6372693536498740	0.3623691886260418
0.2924219703414926	0.5703065553084963	0.3608539759404204
0.2920664742578563	0.4295602693468408	0.3609406095707086
0.4283104091395670	0.6387545461250994	0.2921319852306898
0.3604107426005392	0.5703866393113270	0.2926571181357174
0.3604158316591928	0.4296408584276241	0.2927014591304566
0.3603269499163693	0.7070905409130489	0.4285135222280566
0.2918500055137814	0.6388194498273296	0.4288211737048332
0.4993209488933330	0.6389652241002087	0.3609152183871021
0.4312110757684612	0.5699592561253090	0.3597640709916534
0.5699200547094816	0.5687493716597135	0.3597222196774943
0.3598739732433515	0.4999067629576804	0.3602499901188549

0.5019203196752083	0.5002614739584711	0.3579325897772069
0.6400902259943076	0.5000810483607202	0.3599300750992823
0.4311914736781130	0.4306170159945664	0.3600329971204036
0.5697889380830904	0.4313533679785320	0.3596866253213410
0.3624500792655490	0.3632513799198958	0.3629014208763908
0.4989948475486760	0.3613658849555368	0.3611530417248734
0.4281466827245840	0.2926635338533295	0.3611546394603404
0.4297353258545638	0.7093978869474027	0.4993190404819819
0.5704068837033618	0.7109776183702794	0.5003671078785987
0.3607370147870159	0.6389216814352322	0.4993358153446509
0.5014100685134364	0.6420110001350064	0.5009482796337915
0.6403092511338152	0.6400863762072327	0.5002095790180183
0.2890965515428295	0.5708095881626647	0.4991932813192085
0.4303057796969403	0.5707299853654927	0.5014760225893231
0.5723673117654955	0.5717732685848379	0.5003961829795027
0.7104473594467061	0.5702176796716202	0.5002478231892125
0.3585604196492457	0.5008612263754473	0.5007032948356253
0.5013494999960484	0.5004673661757946	0.5004814408611213
0.6423616762550250	0.5001216957088802	0.5002949492370724
0.2900840224904265	0.4296851952132281	0.5006385193212725
0.4306031187257285	0.4303963560776094	0.5001202977945007
0.5720183246477298	0.4287023253131870	0.5000774338039707
0.7101035627671644	0.4296550208584498	0.5001687538273153
0.3597117463114369	0.3604692605744125	0.4995670935661622
0.5017656491909702	0.3579982819645254	0.5001142014965652
0.6399612473970093	0.3598535911710049	0.5000989781009594
0.4296053385841144	0.2912247977369928	0.4997748108462203
0.5700841170204206	0.2884679829829826	0.4998690031200704
0.5000861597520332	0.6399753913823889	0.6402093620996919
0.4315361397551313	0.5694560103977755	0.6401317815296456
0.5695776934289705	0.5692531760671647	0.6408426999486068
0.3605287699066024	0.4993369956236255	0.6387999761920323
0.5012492757369426	0.5007801875492033	0.6420697787690584
0.6401332963655013	0.4999042532702312	0.6403410151017204
0.2915747510281186	0.4289070191613747	0.6385187082897241
0.4301963696939151	0.4304050170953917	0.6403572933292324
0.5696884106743277	0.4310679845489191	0.6403703560181173
0.3616738941739790	0.3623867731357814	0.6369838667108716
0.4989811900844669	0.3607839811059135	0.6388449300766829
0.4279455452918653	0.2920996956323186	0.6384666252844965
0.4996633693222619	0.5707490254946670	0.2898277678652498
0.4298218937967253	0.5001016095590183	0.2910669782766108
0.5702841134496123	0.5000194298815771	0.2884275595233612
0.4997100102157063	0.4291919798264385	0.2898860958350357
0.4282060549022059	0.3614704796169721	0.2923947262758532
0.4993533948296424	0.7095803843554913	0.4295961666464164
0.4304597227800177	0.6404758651402687	0.4304698001327060
0.5699198342905711	0.6402908496977406	0.4313010690010640
0.3599004800985701	0.5699990098355106	0.4311805739659988
0.5020174423661492	0.5707215700878294	0.4296303567929172
0.6405167440584658	0.5694795658008632	0.4308018287671474
0.2901832126485046	0.5009639868509670	0.4294180166893876
0.4306862693323666	0.5002736744793280	0.4302913201232486
0.5720972512777298	0.5000577059709096	0.4287528506350609
0.7101684398494180	0.5000022636191959	0.4298202355792387
0.3598948067751817	0.4311029405591737	0.4308272364918963
0.5020364072080891	0.4298163912178354	0.4297181496908553
0.6406562880226463	0.4305275820043343	0.4305841056635915
0.2919986212304169	0.3612953848954364	0.4291284407036683
0.4311189208496219	0.3602469646831541	0.4303537919178030
0.5697207049569680	0.3597517009309040	0.4312605851578497
0.3602875389084563	0.2929892560193462	0.4292280788805842
0.4995867332617944	0.2900628949390124	0.4289286395298559
0.5004110937643063	0.7107341222275384	0.5702488066397542
0.4316346493056049	0.6400721342585277	0.5695685221572487
0.5696552925625499	0.6406275663236293	0.5695035931120316
0.3600008771392760	0.5691323552750592	0.5690712706408086
0.5007778691367049	0.5716356762293695	0.5717590980684252
0.6408710076940998	0.5694933873165346	0.5697602184807201
0.2890021874058775	0.4993489763349073	0.5705873280024786

0.4302173533611771 0.5014690912668529 0.5706883176571985
0.5722899067557826 0.5002280097970461 0.5719107499747734
0.7103690994741503 0.4999481926612099 0.5705311686739090
0.3597254468098202 0.4312749799152066 0.5697812304240581
0.5018539099872684 0.4295688486968152 0.5706869238826532
0.6403773554870019 0.4306037388063758 0.5696110237480501
0.2921715373408659 0.3610376727018082 0.5698958572310766
0.4309635091816132 0.3598015817474610 0.5697377055003791
0.5696979564492335 0.3596269554758730 0.5687132314040694
0.3601049366059898 0.2927867893143573 0.5699701467772352
0.4993710409125759 0.2898151902569110 0.5705121579240037
0.5002467758143183 0.5699671709514885 0.7108869943090044
0.4294652137460179 0.4991503344389713 0.7093641907692967
0.5701653499556968 0.5000607285914234 0.7111395496512640
0.3599730686091376 0.4284542620953927 0.7068979827893753
0.4990151295693798 0.4293736804696681 0.7095514754368949
0.4280720187115898 0.3605497345836572 0.7070072580360789

$Cu_{151}Co_{50}$

Topology (208,24,36,6,56; 196), E^{PBE} = -823.00462 eV, magnetic moment = 89.50 μ_B $Cu_{101}Co_{100}$g326

Cu Co Cu151Co50
1.00000000000000
25.0000000000000000 0.0000000000000000 0.0000000000000000
0.0000000000000000 25.0000000000000000 0.0000000000000000
0.0000000000000000 0.0000000000000000 25.0000000000000000
Cu Co
151 50
Direct
0.5000641388418996 0.6393208176078947 0.2195530857621040
0.4294507004601520 0.5702519385373962 0.2166939581386307
0.5705174274067096 0.5701222598486447 0.2167057888733751
0.3602611231389766 0.4998157207539793 0.2191272988690955
0.6395016245394182 0.4996997008381869 0.2189681179219623
0.4290251243284962 0.4289081160222210 0.2163739414596712
0.4997285330659427 0.3599376333499332 0.2187503157241361
0.5003160747203363 0.7831062439860826 0.3589273737879595
0.2186445832220869 0.5000225435632606 0.3599787443790212
0.7828404789704404 0.4998081352987976 0.3584908147691127
0.3585177482183193 0.7829522153771874 0.4999979446597731
0.2175257731805618 0.6409316722972852 0.4997688739231573
0.2134899848073506 0.4992773117132910 0.4990390920657089
0.2184293562314923 0.3601799198356868 0.4996748327595874
0.4999404364451023 0.7828459574267579 0.6415415464117461
0.4260027050363929 0.7155870713527611 0.6445922661976053
0.3552141612074560 0.6444700481812266 0.6444415043477161
0.2842519126726504 0.5732106907651312 0.6441991508259539
0.2169935578396293 0.4997763409982950 0.6408839374477352
0.4998061779258136 0.6404270548277162 0.7820419599086184
0.5714836256229568 0.5709400669810323 0.7844717387493066
0.4282627428212624 0.5708870688119239 0.7843094846822599
0.3584793103498112 0.4996151964615828 0.7820163199729767
0.5002428759729743 0.7122394815613177 0.2882115050332726
0.2889324130338453 0.4996207717106375 0.2889261642031645
0.7111306177900438 0.4996419422325054 0.2887702821559912
0.4285569290667843 0.7848934515223098 0.4284155070682628
0.2855756513004920 0.6436500571817356 0.4265522047438439
0.2164858079842848 0.5706771819975641 0.4286026987491462
0.2159245494813682 0.4289413193313295 0.4286161668884064
0.2876416807196840 0.7123029551391871 0.4995877412964718
0.2887039140218721 0.2892708928866749 0.4991805276301202
0.5718553211417922 0.7848886155570413 0.5718024644697446
0.4282255836492656 0.7848946516958218 0.5716186138708617
0.3553100745556345 0.7154957707862979 0.5736271937092740
0.2845916117023767 0.6442159613258222 0.5732846845470078
0.2151168719748578 0.5711210180734895 0.5711014824632955
0.2159540274433283 0.4288100894335776 0.5703422867757855
0.4998174027839517 0.7126038471654905 0.7126056406713766

0.4259052642626635	0.6443566288961964	0.7154215491102605
0.3551110913212211	0.5733499561405626	0.7153019670473644
0.2869016345916585	0.4994850541256430	0.7124366813589924
0.4999202515753219	0.4991567316422220	0.2138619184566182
0.5706625888123132	0.4288323791957022	0.2160924826383653
0.5741538161591804	0.7156746299789527	0.3556113451050079
0.6433495671894689	0.6431540074942905	0.3569341544503334
0.7143880892858233	0.5725679873142824	0.3565506571108460
0.7143451396239621	0.4270493906196721	0.3565287142002990
0.6431377706984418	0.3571355670371117	0.3568357325764444
0.5732486262188772	0.2858467075209305	0.3560237189343858
0.4998086615088580	0.2190080847496835	0.3599493543903124
0.6417261304487494	0.7828890315955532	0.5002306463194149
0.7823221594326736	0.6404329553253510	0.5000261652160762
0.7883682829739997	0.4993393153663951	0.4999530246182058
0.7830105290665335	0.3586786885606099	0.4999247079614911
0.3599688295207788	0.2194419450332687	0.4995466168134067
0.4997845590617290	0.2148526192727284	0.4993355669777805
0.6395565979754694	0.2195061576254771	0.4995953039831301
0.5739084008088756	0.7155338076553196	0.6447415876825481
0.6449468827177554	0.6446086884378011	0.6449495320034507
0.7157208446248607	0.5732630965574244	0.6449931372040670
0.7822713210721246	0.4994756116254044	0.6412267589922138
0.7145588062987621	0.4263083154584188	0.6436605995771464
0.6431424916987881	0.3566892741605680	0.6433376428926620
0.5728837494757513	0.2860712708513535	0.6432646662521183
0.4996595666462567	0.2195228374742268	0.6389740192982495
0.4998858933599359	0.4993148866138456	0.7886229217901035
0.6410334402004931	0.4994470585190592	0.7825220631696783
0.4284096796318980	0.4283434037623376	0.7850616510824454
0.5711504783449129	0.4281828490589181	0.7852942959620930
0.4997006060456958	0.3586484109158414	0.7832585635244156
0.5728889476388873	0.6431351201057853	0.2862826410952147
0.6434345463123040	0.5729975015971046	0.2855312916415558
0.6437792768481576	0.4262086945594452	0.2853068776374910
0.5732484252758363	0.3563317307075846	0.2854423749716961
0.4996779932476445	0.2889580995198460	0.2888756129724380
0.5718893784761975	0.7848687174587398	0.4284591164372870
0.6447911333494466	0.7156576794156987	0.4262663788718206
0.7143973752332943	0.6430779706418307	0.4268095371825454
0.7848156060051726	0.5709361615171121	0.4283894445672015
0.7851930249701747	0.4284353798102397	0.4284974763072238
0.7142815386347887	0.3569596845388742	0.4270930890767530
0.6435131706549829	0.2860768112370347	0.4261915906242131
0.5705552709662611	0.2168265535650793	0.4289560150542130
0.4291654408102231	0.2169121078998223	0.4290341066280200
0.7125948517975045	0.7122326007583738	0.4999410409433755
0.7110974251372281	0.2892199825771101	0.4994551674188430
0.6448313000268219	0.7154753525588536	0.5739679656092402
0.7155655500896401	0.6442573468571386	0.5737756763597976
0.7843366727501166	0.5708643478954056	0.5715294442130964
0.7850107893255961	0.4281438373158511	0.5714131318569473
0.7142604323006934	0.3566993649006556	0.5727315907107779
0.6433840875394826	0.2859152279006754	0.5729852891492058
0.4291133449622479	0.2170641370696906	0.5700602675836761
0.5703903034597909	0.2171058553439333	0.5701268231538432
0.5738818479898599	0.6443996014262589	0.7156143053525135
0.6447039467167407	0.5733110698023071	0.7157414251055050
0.7126852564225862	0.4992436843257375	0.7129995071891612
0.6432478818071142	0.4263504514720197	0.7146666997129643
0.5724287772656085	0.3566700566337623	0.7142934958218390
0.4995181009389362	0.2892141259785674	0.7109053806249916
0.4264338891658814	0.7156420993208863	0.3557311612304008
0.3571313512562686	0.6430914448529947	0.3572838614631655
0.2856846644024424	0.5731456574514525	0.3562271667244184
0.2854061919706217	0.4265191054711948	0.3561049086498335
0.4274000471756467	0.6430093782571674	0.2864572142599635
0.3566831040224944	0.5729508232638083	0.2856854876891049
0.3563147587494328	0.4263348076810450	0.2856493105103371
0.3555692120535918	0.7157165881231574	0.4261196724929812

0.3571465095523756	0.3572710265225077	0.3570465741205043
0.4264235243139880	0.2858590982693681	0.3562399778895426
0.5002146001501825	0.7879786806372006	0.4999034028321430
0.4282704655023541	0.7153220944048371	0.4999836832358346
0.5720335537140552	0.7153122262556433	0.5002198409957954
0.3576989889753553	0.6419134085261321	0.4992473210763620
0.6426806585752391	0.6418401683830813	0.4995372857451374
0.2855581863173586	0.5710004347193719	0.4998324524810370
0.7146364376651483	0.5702750511968232	0.4994048512098259
0.7145546450490637	0.4280921408089303	0.4996506921424193
0.5000689361717324	0.6423505791280666	0.6427022616359739
0.4282477758390204	0.5711188079036703	0.6431903340864672
0.5719284497916598	0.5711063625633221	0.6433963589207070
0.3573430426063329	0.4992229352316753	0.6425620178749527
0.6428166439634120	0.4991987239997237	0.6428721903751047
0.2849437953172517	0.4266720939400628	0.6435970640279948
0.3567596338617673	0.3574137804849351	0.6427947504436057
0.4265078240488765	0.2863108855004030	0.6428961647640798
0.4264347817282327	0.3564030146896537	0.2856136971585460
0.5002896271109941	0.7153206109306262	0.4282919629446244
0.7147588293281489	0.4990097205650129	0.4282503313410616
0.2851975119346464	0.3562788859561239	0.4259186287901155
0.3563124387987950	0.2860491495244472	0.4261690202384192
0.5000672502666607	0.7154016914369062	0.5718160530124673
0.4284144013668644	0.6425305089732665	0.5714865989419343
0.5718742756870299	0.6424306153575299	0.5716119173617620
0.3567103211240761	0.5711031710872315	0.5716522862279613
0.6439003261814060	0.5711573625948744	0.5719580754786860
0.2853468546845347	0.4994156665145460	0.5712054666380580
0.7145301751030145	0.4987289933199009	0.5710673068469166
0.2855213277131762	0.3570301446222334	0.5727064286435352
0.3562881254024049	0.2863094469158049	0.5725009083818754
0.4999780792295071	0.5702443294338161	0.7146092046204129
0.4288109925048670	0.4988664668229423	0.7144622039467564
0.5710577178569255	0.4987396522665264	0.7146680039216491
0.3563844379191672	0.4266041070499205	0.7142593951850597
0.4998174966744350	0.4280571604485931	0.7148719034981348
0.4270041798985860	0.3567588879291109	0.7142231359358934
0.5000367071763433	0.6416003193101735	0.3578358335172766
0.5001705854902901	0.6431237257414161	0.5000084128678793
0.4287525604983166	0.6421369778590814	0.4285003898359885
0.5715442751653459	0.6422992038572861	0.4284729593643897
0.2889880140265282	0.4286675156539549	0.4981889408136237
0.4999954996942778	0.5681943397916577	0.2880022711477132
0.5698876745639414	0.4987374228595106	0.2886666330116056
0.2891977900430678	0.4987935151372196	0.4289085090348719
0.4289652988362157	0.5674739861366201	0.3581182074542754
0.5705686709129473	0.5677884284952576	0.3580318382507879
0.3600189210627622	0.4987693242009149	0.3593617841021751
0.5001383264927602	0.4995297678308539	0.3582648857663572
0.6388376671219047	0.4991874954298526	0.3582592652074890
0.4309456051467181	0.4306292117357054	0.3590409923697871
0.5689614321187190	0.4302812019554430	0.3587710568207115
0.4997708481403453	0.3597434737411072	0.3592743463126625
0.4294191714530640	0.5675929538774652	0.4996655418040421
0.5708560624414059	0.5674506217759828	0.4996001891495652
0.3587269921788940	0.4981045663089627	0.4988942880360909
0.4998531389460960	0.4994076299074222	0.4994396291917082
0.6402379018411214	0.4987125068547234	0.4988724073679875
0.4284471474956918	0.4280207254020580	0.4994885543477718
0.5711397327885672	0.4277824856129898	0.4997002472957647
0.3593166541006312	0.3602296800861680	0.4985912197580822
0.5001432570000313	0.3576552068331231	0.4992357922509674
0.6386202698836474	0.3586932420087082	0.4996173459698355
0.4295647721037917	0.2891624044079006	0.4995497011809830
0.5700016138468593	0.2891041356504782	0.4993512720575327
0.5000928185886544	0.4977490569051572	0.6395111665034192
0.4308677867261617	0.4282823131225832	0.6392222012549087
0.5691666370317786	0.4281568215243982	0.6392552877761664
0.4995698858060507	0.3592463536757695	0.6384311369640285

0.4302903518234132 0.4990865130248395 0.2887019653799480
0.4999445207563132 0.4291903860605671 0.2884089807782589
0.3582933645436568 0.5673249160117417 0.4279086739874770
0.5000135324513612 0.5676239509721550 0.4290176354654107
0.6404737032699145 0.5680668181121957 0.4275384279420465
0.4285330238492863 0.4997173330782243 0.4283489456490960
0.5711901345764089 0.4994550642064236 0.4280443366458471
0.3594668461404695 0.4303716954838186 0.4298710228753767
0.5000763730884233 0.4278592740817068 0.4284338892256551
0.6394243743309318 0.4295673655780597 0.4296867402936093
0.4305860064822504 0.3590051592444192 0.4302703610499997
0.5691215914170239 0.3590663862137076 0.4298176184020111
0.5000234441003888 0.2890810930953126 0.4289660783552236
0.5001661556992625 0.5674097029840312 0.5699275143391147
0.4300226247181417 0.4981605455577512 0.5695466361263160
0.5704267393851147 0.4983921729666432 0.5693569867900996
0.3595998680081843 0.4290515001093034 0.5685368154909951
0.4999799919107644 0.4281182079068290 0.5701470964841786
0.6391918565043964 0.4280235624142184 0.5698548023978508
0.4298730889539575 0.3590325952731743 0.5681803065675097
0.5691049037160965 0.3586563100552868 0.5688962106104790
0.4996872635433195 0.2890681081303420 0.5693407458788446

Co$_{201}$

E^{PBE} = -1291.0616 eV, magnetic moment = 302.04 μ_B Co201SPNC273.2-1291.0616m302.04

Co
1.00000000000000
25.0000000000000000 0.0000000000000000 0.0000000000000000
0.0000000000000000 25.0000000000000000 0.0000000000000000
0.0000000000000000 0.0000000000000000 25.0000000000000000
Co
201
Direct
0.5001655535607484 0.6380676028022317 0.2247686568644067
0.4307512377294490 0.5693922516324001 0.2235637603408225
0.5694820743225688 0.5692765016334657 0.2236007064324720
0.3617918085326909 0.5002474485145858 0.2247001723553900
0.6383392192971495 0.5000518252814422 0.2247565997175804
0.4306308971022115 0.4309671972324804 0.2235100716903400
0.4999793209642386 0.3622013308062127 0.2246542804069245
0.5002947913323681 0.7750019151639463 0.3616899838176788
0.4309483977637552 0.7084104632360366 0.3611298690325579
0.3605280190153546 0.6397077481864587 0.3603730060955008
0.2915640643965084 0.5695182025035885 0.3610349340824296
0.2247796766696999 0.5003416998449693 0.3615202367017943
0.7753097199349627 0.4999022340555573 0.3615597815225308
0.2913970944432770 0.4310108648209331 0.3609581227402985
0.3620842301813906 0.7751685213767712 0.5000895666664045
0.2249020607254216 0.6384080718673143 0.4999768990411014
0.2228255501090012 0.5003112715643285 0.4998072621893062
0.2245462895632570 0.3621353486979866 0.4998578054784820
0.5002143057226840 0.7748835767497516 0.6384249586673256
0.4308332859335873 0.7083868983995281 0.6390442778731718
0.3603102833817891 0.6396665015066662 0.6399029887939516
0.2913361619916342 0.5693938078220883 0.6389553500398739
0.2246900567269132 0.5002013493095201 0.6382318057470070
0.4999877962500862 0.6376911521507929 0.7754986303801418
0.5691315231590779 0.5688434943150227 0.7765093445999391
0.4306840782755297 0.5689785251450112 0.7764436171379347
0.3617455592536456 0.4999047562117352 0.7753672906943738
0.5002228612455473 0.7068304781485670 0.2929242214286899
0.4308515144439733 0.6388673727436210 0.2914401498827232
0.3611795442762147 0.5695029184821352 0.2913881577300214
0.2930115467460723 0.5002799976014954 0.2927890670852094
0.7071125150506806 0.4999727256410658 0.2928461094231111
0.3610503565575420 0.4309375707769352 0.2913154417687750
0.4309959241673921 0.7762820721540359 0.4307122196658283

0.3614270860372449	0.7085030837904081	0.4307359388562234
0.2916349948630699	0.6389412845420672	0.4307057216127925
0.2236162823109268	0.5695903790826381	0.4305683338589552
0.2234291246097717	0.4310327093649574	0.4304892882584029
0.2932932739031500	0.7070326436263439	0.5000220269578473
0.2927852985907498	0.2933882233915261	0.4998263671285912
0.5694728580312854	0.7759591637016572	0.5694439271262194
0.4310167457295904	0.7761353967576402	0.5694027734381694
0.3614275185590258	0.7084614370086521	0.5693971510537236
0.2915616936411152	0.6388300291606598	0.5693207546751684
0.2236459073248401	0.5694709847919084	0.5692517503146315
0.2234516977620663	0.4310018942924216	0.5692023886559548
0.5000947466548096	0.7066049001075378	0.7072149029291429
0.4305508766626651	0.6388585797052921	0.7088547612714409
0.3608261312909269	0.5694220200216994	0.7088159199716860
0.2928809421670204	0.5000447573540207	0.7070614775440047
0.5000566566400997	0.5001517647610796	0.2227467569797762
0.5694041707782366	0.4308845604989728	0.2235494694535618
0.5694971109199467	0.7082566464904507	0.3611786554865972
0.5001557190362914	0.6401454414106977	0.3596239563982273
0.6398105569800399	0.6394526005213237	0.3604550616434138
0.4293147843407188	0.5707606404240494	0.3599497900549502
0.5708794823723682	0.5706426056782979	0.3599899758957538
0.7086716522784869	0.5691840924630260	0.3611126731541518
0.3596407291078381	0.5001870731355679	0.3595126540195711
0.5000228684062700	0.5000775755573414	0.3617316622504530
0.6404627796958509	0.4999739559096671	0.3595627019852736
0.7085831926973236	0.4306839771618990	0.3610346624953128
0.4291794024198287	0.4295027854761139	0.3598772865716615
0.5707862759916980	0.4294074982975081	0.3599199061490007
0.6396151218976603	0.3604896730557807	0.3603053977567113
0.3602451387119108	0.3606893917090785	0.3602285639285409
0.4999340520586230	0.3600283935819926	0.3595148013346384
0.5691656687271618	0.2918096925973080	0.3610109231874620
0.4306126821377528	0.2918868562975995	0.3609674354551721
0.4998463334672283	0.2251528909881657	0.3615813658752140
0.5002894836255162	0.7766943678358250	0.5000208397059432
0.6384367063015375	0.7748816510516444	0.5001704119229822
0.4296805491337519	0.7102010226792977	0.5000002669385377
0.5707158746062397	0.7100151604615426	0.5000386456350198
0.3597938623669470	0.6402857417993152	0.4999855745195719
0.5001163307468207	0.6384670647993858	0.4999892425782996
0.6405009085462848	0.6399783316519869	0.5000770260180385
0.7753903083145213	0.6378810099284101	0.5001611825205329
0.2896625224413617	0.5706106736633770	0.4999019303170387
0.4313353701464192	0.5686410868635404	0.5003057973707467
0.5687763254066097	0.5685107570418855	0.5003485677899017
0.7105250033024940	0.5702241564100559	0.5000371514512465
0.3612702455002397	0.5001222158852958	0.4998853529409715
0.4999871483008377	0.4999768996955460	0.5007582676468294
0.6387444952965035	0.4998868003137115	0.4999831820871912
0.7771998900388886	0.4998058138491359	0.5000033621696308
0.2894524906093234	0.4297794572065908	0.4998427382671837
0.4311983927564813	0.4314497758270557	0.5002445731772911
0.5686810716349580	0.4313489795562838	0.5002828931549105
0.7104452601026769	0.4294769983569375	0.4999694031570896
0.3594466753303833	0.3599910549194995	0.4998444391530266
0.4998868623227045	0.3615551774227330	0.4998453750999379
0.6403292572984401	0.3598106821801015	0.4999294092662828
0.7752335309579498	0.3617500568194052	0.5000234956101149
0.4292418427317181	0.2899892673942154	0.4997747213440583
0.5704370222099030	0.2899121849801118	0.4998081489129463
0.3614889229857485	0.2250820722802351	0.4997770075328138
0.4998006639591691	0.2233414761971916	0.4996832917322332
0.6381127606094391	0.2249447609081331	0.4998539218473646
0.5694267956496726	0.7082228670124262	0.6390868482809459
0.5000531925569504	0.6399851555643481	0.6406826894650096
0.6398105079460956	0.6393903765839637	0.6400090016184959
0.4285854570491683	0.5712231552237952	0.6403853354773601
0.5713968264222293	0.5710958349155932	0.6404316541766630

0.7086709502187375	0.5690350313237700	0.6391464610806118
0.3595388879708467	0.5000081324850938	0.6405813686309805
0.4999282405782241	0.4998908916893566	0.6357838702743038
0.6403210771219182	0.4997870569296835	0.6406952505817893
0.7752061091720276	0.4997439104247883	0.6385194093317823
0.2911588604239887	0.4308075733667967	0.6388876450030062
0.4284493286422604	0.4286579334839987	0.6403158641682771
0.5712908616713261	0.4285616348761794	0.6403596603125024
0.7085869348828533	0.4304857630993704	0.6390766633813780
0.3600219111326646	0.3603657545103114	0.6397344020739471
0.4998212357069857	0.3597722283838929	0.6404849889180173
0.6396188430822480	0.3601725085431163	0.6398414743050104
0.4304677375592536	0.2915174371756869	0.6387498697818914
0.5691068444301525	0.2914410551913160	0.6388050846626343
0.4997560578840711	0.2248942433641760	0.6379303756096110
0.4998446228717624	0.4997752392505912	0.7757393251677732
0.6379700546872463	0.4997001862636147	0.7754931260648250
0.4305646797248339	0.4306745929470550	0.7763263160333380
0.5690315604006280	0.4305975710480917	0.7764046881942138
0.4997756477038224	0.3618294991296783	0.7752857624158810
0.5694704431798926	0.6387444501204996	0.2914722273998470
0.5000979085705215	0.5704499924125407	0.2896916534451631
0.6390438720341944	0.5692909231754348	0.2914479716312182
0.4294908547458600	0.5001550760851289	0.2896472957019487
0.5706046094116617	0.5000582007567835	0.2896930777648389
0.6389511620129127	0.4307353392817462	0.2913788560111770
0.4999909715963481	0.4297654092403103	0.2896253794307274
0.5692674174364823	0.3613633694870686	0.2913439403182594
0.4306378173404504	0.3614469159138677	0.2913053153444193
0.4999232632875914	0.2933770406678765	0.2927740789083986
0.5695723555387080	0.7761245780739097	0.4307633034208615
0.5002078127741280	0.7102693216311864	0.4295058700563264
0.6390124195703714	0.7082088956225049	0.4308286538350068
0.4295063806436631	0.6398872187454018	0.4292727815591018
0.5707881427199767	0.6397294645522875	0.4293178282834055
0.7087095162148602	0.6385458653491879	0.4308279395813220
0.3600819991964434	0.5707039781086052	0.4292089923582689
0.5000696454749609	0.5685037832382734	0.4311899243023826
0.6401166622980049	0.5704541885858074	0.4292813389602983
0.7765960967443788	0.5690899796526909	0.4306821156998092
0.2894617065538752	0.5002287047022316	0.4293667819224123
0.4314120054008196	0.5000778144118667	0.4311503367966855
0.5686146812378416	0.4999758272696361	0.4311944940818249
0.7106296818208374	0.4998967346038163	0.4294445934823357
0.7765138945907298	0.4306105820273418	0.4306045592416530
0.3598927203018740	0.4296205032068848	0.4291413717106841
0.4999648359322602	0.4315505726567438	0.4311226116813475
0.6400465738930979	0.4294266757672701	0.4292136561912985
0.7085467239047810	0.3612418065985943	0.4306777855962969
0.2912800890382399	0.3615308447086675	0.4305655595868313
0.4292265551434696	0.3603338513239051	0.4291442778587711
0.5706018676359941	0.3602556688217003	0.4291819566932857
0.6387586329472789	0.2916920354598564	0.4306113327095129
0.3609756720521857	0.2918623514408564	0.4305414051503647
0.4998651361704786	0.2898923707287174	0.4293378634849358
0.5691992351821297	0.2239115732678595	0.4305215534443076
0.4304680352849312	0.2239707863739476	0.4305008368269087
0.7071400983115955	0.7065942966656865	0.5001484411681874
0.7068805974799813	0.2931110577819418	0.4999396127442308
0.5001577572672052	0.7100300234978787	0.5706938387116914
0.6389167035107194	0.7081554932767393	0.5694771126587040
0.4292322785068783	0.6399840227708163	0.5708030556525285
0.5709562781641933	0.6398338152757910	0.5708377114923325
0.7086726915939827	0.6384289039224790	0.5694792455938403
0.3598331831593631	0.5708346939005167	0.5707552459579013
0.5000194823635091	0.5686836993572686	0.5691786964168055
0.6402512129711000	0.5705764837379572	0.5708647348958332
0.7764544424968222	0.5689580793498948	0.5694972124007376
0.2895400027972052	0.5001448833443967	0.5705267883211850
0.4310782710726498	0.4999932335362727	0.5691155664503064

```
 0.5688405224258565  0.4998816225424642  0.5691675043886734
 0.7104221464694382  0.4998076283132374  0.5707136232626828
 0.3596641206565597  0.4292951994145681  0.5706947828945237
 0.4999055630283744  0.4311864448623107  0.5691104269804365
 0.6401635583123294  0.4291037982422807  0.5707938146568695
 0.7763601042950353  0.4305752784726954  0.5694417205438900
 0.2911977002176971  0.3614760001102000  0.5691948325690832
 0.4289463508966296  0.3600313917737624  0.5706402204597494
 0.5707681029437540  0.3599424368268285  0.5706839587992255
 0.7085153329645455  0.3611888364619104  0.5693391370557144
 0.3609678981366223  0.2917158961052282  0.5691483000132427
 0.4998106627394742  0.2898949136686608  0.5704037315649563
 0.6386710682123914  0.2915520535854074  0.5692447448928221
 0.4304751071408636  0.2239217492350922  0.5689864279329500
 0.5690958400425280  0.2238723485646265  0.5690475544429066
 0.5694878994691385  0.6387188715436568  0.7089211561030714
 0.4999495677772525  0.5707121265410738  0.7101848787254613
 0.6390707900337254  0.5691839902647610  0.7089354543724481
 0.4288354777572636  0.4998859254095469  0.7101130584433392
 0.5709410939580313  0.4997779158019346  0.7101608468251430
 0.7068963837357971  0.4997228055681329  0.7072630831150105
 0.3606822098807606  0.4304897055265555  0.7087297836774737
 0.4998368896056610  0.4289284742845443  0.7100882124293806
 0.6389797836039924  0.4302898402794805  0.7088485249098588
 0.4303122447353367  0.3608975372054798  0.7086688259650574
 0.5692726777423109  0.3608242634745925  0.7087260255629009
 0.4997726269374426  0.2930039355732227  0.7068687017762589
--------------
```

Cu$_{201}$

E^{PBE} = -662.4334 eV, magnetic moment = 0.00 μ_B Cu201NC273.2PBEfin-662.43338m0.0

```
Cu
   1.00000000000000
    25.0000000000000000    0.0000000000000000    0.0000000000000000
     0.0000000000000000   25.0000000000000000    0.0000000000000000
     0.0000000000000000    0.0000000000000000   25.0000000000000000
   Cu
   201
Direct
 0.5005989779099130  0.6418068978589753  0.2164284648995806
 0.4280656777221478  0.5721931931844689  0.2148558063240145
 0.5725652029534164  0.5717497966004393  0.2150575956349984
 0.3577887509259852  0.5003753551342038  0.2165390656556946
 0.6427093547032667  0.4997317416698130  0.2171499960483532
 0.4279051168128686  0.4282796660090857  0.2151006960953830
 0.4999667343270990  0.3582683035123658  0.2169084820266383
 0.5008538071887824  0.7822598576483072  0.3578240535261371
 0.4260308813130161  0.7165486646402233  0.3539291747827475
 0.3544413774188127  0.6459669091416654  0.3540934856612724
 0.2832192645703956  0.5750795109714242  0.3537893530764524
 0.2169562650637240  0.5006129023736797  0.3576002996496737
 0.7836380607339397  0.4995415790987250  0.3578692141801809
 0.2832868756404763  0.4262644833954591  0.3538799712626606
 0.3583842553079306  0.7829378580827203  0.5002136829641675
 0.2169948348081877  0.6425760209613481  0.5001327138435624
 0.2119789335778055  0.5005079112372793  0.4999057558148587
 0.2169429152308386  0.3584094046099288  0.4996714776693283
 0.5005648418820382  0.7821381709056384  0.6426687245272321
 0.4258466254454019  0.7162974961421628  0.6463510156952876
 0.3543286895212455  0.6456320142357630  0.6460795340110138
 0.2831894053167033  0.5745830733333422  0.6462730753192887
 0.2169915632026354  0.5001111030584513  0.6423369900615672
 0.5002485178136983  0.6413850973876744  0.7837981711228039
 0.5721881251884176  0.5712921304287876  0.7850712799390033
 0.4278454037817142  0.5716450805254198  0.7852379026023736
 0.3576941641679870  0.4995747224347093  0.7834292181637529
 0.5007786602043122  0.7137740961020254  0.2855067722279424
 0.4258936211345835  0.6460151260400118  0.2828291022666437
```

0.3540659952222232	0.5749605149876774	0.2828153849073787
0.2856209347537641	0.5006522687079079	0.2853183493178484
0.7146808945885131	0.4996409234805290	0.2859393927792619
0.3539991685084936	0.4261489258518463	0.2830663775035822
0.4285292441831672	0.7844986368806487	0.4279691459524653
0.3543386523809084	0.7169246626569478	0.4255021836125212
0.2833020986168386	0.6463964788166709	0.4254835153962450
0.2151539027654684	0.5726251724234622	0.4278055536357989
0.2152422684446778	0.4284793882788526	0.4277192379095744
0.2858479221006558	0.7146287715785721	0.5001823548732608
0.2856365747134421	0.2862125438490213	0.4995608672554498
0.5724677284674309	0.7840010862308696	0.5723087467107293
0.4284073145971820	0.7844632190027329	0.5724411211761311
0.3542375807585008	0.7167609785619100	0.5748625457328180
0.2832367701584544	0.6461834196007719	0.5747622302153506
0.2151458525404243	0.5723507788608431	0.5722512666618133
0.2152434963972808	0.4282755753257799	0.5719520443887907
0.5004243308082533	0.7133970244230288	0.7148157377353468
0.4256080208172200	0.6455621226829011	0.7173679877450116
0.3539275248250547	0.5743325088903422	0.7172883797994148
0.2855841601292285	0.4998809845449081	0.7146219617849083
0.5001092307796644	0.5001805053830137	0.2118162711310426
0.5721161161387127	0.4281742000403846	0.2153096386931264
0.5751948893954845	0.7160216235933300	0.3542322152920446
0.5002940822787094	0.6444057070831523	0.3553948570278773
0.6461867216414090	0.6450685300259452	0.3545170420406114
0.4271836295691137	0.5732164617894672	0.3551242278778297
0.5728859374863492	0.5726713200628235	0.3554699441812413
0.7172951710681869	0.5739547405230294	0.3542456238223355
0.3552713761021722	0.5007696271912554	0.3551558433253437
0.4997851816764837	0.5004526839288624	0.3554666385174358
0.6446416383269382	0.4999924012943356	0.3556369767564984
0.7168423180359420	0.4255010628940739	0.3542737005528098
0.4269779371184565	0.4279354696801169	0.3552921749504605
0.5724577617265382	0.4277553084315063	0.3556322982806793
0.6454596244317509	0.3546729325357432	0.3545331000513582
0.3542466220400276	0.3548539478558455	0.3541430362576266
0.4996984033148334	0.3561719638899048	0.3556698519391392
0.5741687579313881	0.2837766133519459	0.3542718530763091
0.4253560415822573	0.2836985281275996	0.3540217351174383
0.4998457528666068	0.2173021993683170	0.3576922208642900
0.5004924483271763	0.7874695745225058	0.5002758071272132
0.6427577377809341	0.7817316365409611	0.5003911269728905
0.4281822989544704	0.7157971764103929	0.5002512129370531
0.5723328920035199	0.7151309902247407	0.5003441401307714
0.3553641891459807	0.6449002252969617	0.5001286462737251
0.4999531452379841	0.6441668768979046	0.5001947655895324
0.6448107219292923	0.6437223447782499	0.5003156767550300
0.7840053863216061	0.6412386585953607	0.5004671778323325
0.2839016794362541	0.5726072409081303	0.5000378330286849
0.4273185511113065	0.5727496933863642	0.5000519522105021
0.5721998297177864	0.5721824584400328	0.5001777601329972
0.7164757351435767	0.5716249426038533	0.5002667103403754
0.3553252066321915	0.5006600104810122	0.4999118525242331
0.4993521732743874	0.5006344164371772	0.4999553485884134
0.6444939485719716	0.5000432060771051	0.5000987674388423
0.7886292641522320	0.4995683805268877	0.5001777158387614
0.2839591558235095	0.4284800158360526	0.4997749677170065
0.4273008444216386	0.4282670498195619	0.4997893911006194
0.5717592966977517	0.4282706358185407	0.4999037693295033
0.7160605072429562	0.4279007026577719	0.5000407496899749
0.3553290494702422	0.3559948849249493	0.4996541279253821
0.4995651109582999	0.3559932495639230	0.4997651384497918
0.6440348823689097	0.3560335699725835	0.4998492838495023
0.7832485800582158	0.3578322978639911	0.4999978496735726
0.4275809333242026	0.2843538115965684	0.4995930484135436
0.5717577682016832	0.2843790512959402	0.4996945448268859
0.3574591886273872	0.2172346228993157	0.4995429919804130
0.4996814026584133	0.2120463514821437	0.4996469138362969
0.6420296296782310	0.2172649013043450	0.4996833126189917

0.5748973472729062	0.7158290660920500	0.6462332150549409
0.5000234012006018	0.6440515016676203	0.6448785227306149
0.6459310412137556	0.6447642978706210	0.6459491644364070
0.4269792692746328	0.5727357835759058	0.6449936045011071
0.5726366278382501	0.5722614866409212	0.6448041769270166
0.7170182738710834	0.5735765958100908	0.6461799881256055
0.3551783162371735	0.5002276715738436	0.6447780408384306
0.4995827084090115	0.5000254658367828	0.6445445607579293
0.6444608027036250	0.4995725380427832	0.6445442146941600
0.7833196779059122	0.4992228048318188	0.6425290820248560
0.2832633940391657	0.4257097167590218	0.6458196891977609
0.4268358898204963	0.4274989664200275	0.6444322662151662
0.5722825656971828	0.4274012980883485	0.6442574932634598
0.7166220834251322	0.4250970072037505	0.6458168259296405
0.3541819170908571	0.3544544716111758	0.6453748870986746
0.4995506104335541	0.3558203507590881	0.6439332543825201
0.6453013567057884	0.3543451480713669	0.6452594073897439
0.4252360560595279	0.2833141901786825	0.6453301267379066
0.5740285229400243	0.2834235856867389	0.6452440937633499
0.4997098051703321	0.2168436300916740	0.6414604380177156
0.4998087910995738	0.4995470421791754	0.7882583462709738
0.6421171345610335	0.4990723276168554	0.7831358569778424
0.4278040297759803	0.4275503643494828	0.7848430004359872
0.5717534821153427	0.4275306818290659	0.7846624501622600
0.4997985841399832	0.3573792541304612	0.7826277226846857
0.5750583071339944	0.6455077660407581	0.2831303937039441
0.5002415582919257	0.5722053558359647	0.2838100985032915
0.6463272189787391	0.5740370161229996	0.2834278178515429
0.4277786666588783	0.5004809157034491	0.2838000634338287
0.5722289734835492	0.5001114787069348	0.2840680809309430
0.6458860888431507	0.4256319071016386	0.2835889573305101
0.4998380858019810	0.4283170253786445	0.2840287163319920
0.5742568688974075	0.3545834777572335	0.2835336685814031
0.4254434482912894	0.3546275111197922	0.2832732639876912
0.4998694458626768	0.2864276805490032	0.2859601939855327
0.5725902579577909	0.7840554061285018	0.4282767460613315
0.5004145582618716	0.7155171851224553	0.4280583391384338
0.6463732924514402	0.7157510781028843	0.4259281616795418
0.4272444655265387	0.6447941033705098	0.4272401654787261
0.5729935638963685	0.6440757628558389	0.4274191677895473
0.7174480665936366	0.6451784090602337	0.4258291919959560
0.3552679485042318	0.5733860253352892	0.4271001006766104
0.4997749851504618	0.5725795897256731	0.4276476841629738
0.6449251593240704	0.5724333849165769	0.4274490902336986
0.7853797472723002	0.5714481564667475	0.4281011192643455
0.2839370162247589	0.5007411383053501	0.4277119686126388
0.4273453336510036	0.5007537988658677	0.4275101483342007
0.5720441461015580	0.5003995176489751	0.4278435731246003
0.7163103731129007	0.4997879060674784	0.4279739905286673
0.7850338326004880	0.4278298951571984	0.4280378598119106
0.3552484285899726	0.4279940209240030	0.4270406678511614
0.4994654681715768	0.4283427163475416	0.4277356350555545
0.6444132946830210	0.4276332448820327	0.4273908698745784
0.7166045345314541	0.3543804358139733	0.4256176667072908
0.2833071057939396	0.3546503451095185	0.4252363724509129
0.4269201658601472	0.3559693161616621	0.4270440821222340
0.5723363758831425	0.3560088633581786	0.4272844112856773
0.6455644308578763	0.2837413652356842	0.4254858004155940
0.3539601200291793	0.2836519591377422	0.4251615809148683
0.4996463367984957	0.2844269806877781	0.4277544840333621
0.5717920839385789	0.2154703452734894	0.4278661524913577
0.4277559476182778	0.2153732999168976	0.4277197052598819
0.7148585026650180	0.7129969937081044	0.5004444560359909
0.7138624836185455	0.2862688647636372	0.4998358304409877
0.5002630912386261	0.7154173557663916	0.5724108949163992
0.6462674950847098	0.7156348413660828	0.5748493552673101
0.4271177039729244	0.6445309252633844	0.5730533555646825
0.5728536519343189	0.6438860102983974	0.5730698265052743
0.7173200255617773	0.6449930345680909	0.5748810494432826
0.3551757314644948	0.5730933860791237	0.5729522364008645

```
 0.4996365478269538  0.5723002435498764  0.5725216743623404
 0.6447854478817067  0.5722291437596975  0.5729588044450566
 0.7853367968881976  0.5712438338252683  0.5724223458057887
 0.2839001952875654  0.5004520273915848  0.5721450634799520
 0.4272421906166483  0.5004621210353177  0.5723781128199753
 0.5719469669441001  0.5001334551844216  0.5722362876314477
 0.7162078257448384  0.4995737244631994  0.5722692855260169
 0.3551926748334953  0.4277414596956324  0.5725664213360729
 0.4993757659792313  0.4281609954486726  0.5720135653367876
 0.6442859728539825  0.4274323485325491  0.5725810099692892
 0.7850093690990724  0.4276902093868257  0.5721306852333100
 0.2832825299640616  0.3544480106222852  0.5740882559499076
 0.4268756800338744  0.3557645233605368  0.5723924649825821
 0.5722484357257407  0.3558172124901282  0.5723711130833343
 0.7165017377596945  0.3542700889883094  0.5742117666595407
 0.3539440765370638  0.2835329480637762  0.5739808664045101
 0.4995943994173152  0.2842546232794909  0.5716459248321719
 0.6454801926831074  0.2836396162436480  0.5740065059413889
 0.4276641236816049  0.2152807762089278  0.5715058049634802
 0.5718123460266957  0.2153562541123843  0.5715479723380046
 0.5746764249365914  0.6450849207966846  0.7171548623539835
 0.4998968922151207  0.5717450576207244  0.7163319346788845
 0.6460062702692745  0.5735148390345391  0.7169096580747591
 0.4276165805423411  0.4998148834897986  0.7162189571208609
 0.5719151860153538  0.4995579134106277  0.7160123969437147
 0.7142953931699745  0.4990575051907822  0.7144144611311646
 0.3539067220075555  0.4254119060257918  0.7167644095919667
 0.4996020555242955  0.4277615589761724  0.7158199834664964
 0.6455996736659376  0.4250468439807208  0.7164682219994296
 0.4252791302993588  0.3540306636612243  0.7163295110110627
 0.5740062889942134  0.3540700887680072  0.7161308026474265
 0.4996659399774465  0.2857822038031262  0.7133821536548625
------------------------------------------------
```

S3. Calculated atomic coordinates, total energies, and magnetic moments of key structures of $Cu_{303}Co_{102}$ nanoparticles, bare and with adsorbed CO

The DFT (PBE) data calculated by VASP code are listed in this section as CONTCAR files obtained with the cutoff energy for the plane-wave functions of 400.0 eV.
PBE data for free CO molecule: E = -14.7915 eV, vibrational frequency 2122.75 cm^{-1}

$Cu_{303}Co_{102}$

Topology (334,24,60,24,96; 445), E^{PBE} = -1703.7701 eV, magnetic moment = 163.89 μ_B
Cu303Co102g2.18ENCUT400fin-1703.7701m163.89

```
Cu   Co   Cu303Co102
   1.00000000000000
    32.0000000000000000    0.0000000000000000    0.0000000000000000
     0.0000000000000000   32.0000000000000000    0.0000000000000000
     0.0000000000000000    0.0000000000000000   32.0000000000000000
   Cu   Co
   303   102
Direct
 0.3816132441029907  0.3818583737765903  0.2122915539580058
 0.4373833752546834  0.3256451040053744  0.2129253357286585
 0.4931953042481782  0.3818711426677689  0.2123157613899977
 0.3816773799033970  0.4933698197856253  0.2120708274666027
 0.4374080232834707  0.4375992634387596  0.2112458635264130
 0.4931247772818465  0.4933211225234291  0.2120886142930453
 0.5492525312921318  0.4376552342845743  0.2129400874308975
 0.4373598881779861  0.5494023336749582  0.2126971674846525
 0.4373202876388997  0.2130066552984726  0.3262562868796972
 0.4372897837942114  0.2695398342079163  0.2697656260120200
 0.3262309401673775  0.3263621333239503  0.3267766036206752
 0.3255968327200758  0.3816267330323977  0.2692656706378806
```

0.4368816207499241	0.3260299103865106	0.3263291682063769
0.5492875402136889	0.3256958155193483	0.3258580437184324
0.2138818717081287	0.4375585034350014	0.3271096280921222
0.2697543752630073	0.4375627923196886	0.2698305515919827
0.3262327280773667	0.4935264021680599	0.2700537352654798
0.5487852719681746	0.4381587812685823	0.3263737614892062
0.6052749635858858	0.4377197733313391	0.2697665442756036
0.6619437134810841	0.4376575795812315	0.3262092346138758
0.3261642099660713	0.5486928343319836	0.3265572476562932
0.4374146700837844	0.6045642058030244	0.2705328165876655
0.5486620379810297	0.5486233691940007	0.3265200151848063
0.4374380787040018	0.6610020013695778	0.3270455413696237
0.2706378490745294	0.2707221602258699	0.4380188510386737
0.3262276505194979	0.2702515491101982	0.3825203411316964
0.3818668008680688	0.2131775314095578	0.3830058360396346
0.4369142315928742	0.2127696154868608	0.4388007483533423
0.4929042074327606	0.2125328756906291	0.3825123771413131
0.5489268755571018	0.2131639295786661	0.4383373523841783
0.6051027374699801	0.2699024941193597	0.4381383797848348
0.2144400517855083	0.3270102661506855	0.4386162025054038
0.2136610599280311	0.3826753584887370	0.3831544706358900
0.6617593924248579	0.3261987325664461	0.4383349378548436
0.6624278775236901	0.3821909972899512	0.3825243359831663
0.2132835184851029	0.4376164713515205	0.4385936245258242
0.2134909052819051	0.4925579035483292	0.3831460613269322
0.6622535281626178	0.4381763866837720	0.4388045090347190
0.6618620036508259	0.4931339836417250	0.3829843037536778
0.2143217723624569	0.5481749263330146	0.4385975678257588
0.2707012522411015	0.6043186235483539	0.4379252464319719
0.3262617734949722	0.6047037392383871	0.3824507912142903
0.6042218158319096	0.6043475060718553	0.4379075400096182
0.6608535751679052	0.5483619795289854	0.4386077364663281
0.3825271452777589	0.6612088263939678	0.3831148237894904
0.4374514854545141	0.6613369663844981	0.4384291419856597
0.4924045533542640	0.6612805791977504	0.3830371262719551
0.5480679004598855	0.6606477898060740	0.4385427070482915
0.3821325064044796	0.2140117191111172	0.4937712944649446
0.4370498274692940	0.2145889580218590	0.5490704391081541
0.4923661464950984	0.2135297500130899	0.4941838947316784
0.2143139836337434	0.3824419685962184	0.4936041637519232
0.6614477310715654	0.3826967905120842	0.4941783969812974
0.2149793732047548	0.4375376985298380	0.5487653782054771
0.2143428146513378	0.4927463695730186	0.4936063641511899
0.6604725727347586	0.4379730555502269	0.5490442199040190
0.6610448166853602	0.4929886175187341	0.4937712498562268
0.3824046777437688	0.6606925678479524	0.4935656762194687
0.4374961600425930	0.6602024811937043	0.5488373756416629
0.4925522541486454	0.6607323669999426	0.4935486856958433
0.3794775207411482	0.2684068610567385	0.2130754709957962
0.4373481769755960	0.2149936581027747	0.2155729080543469
0.4373792807353853	0.2708932015036016	0.1607895952302951
0.4951774575081134	0.2684917671960210	0.2131590022091781
0.2684216627036080	0.3797134892075884	0.2131670363351348
0.3240957843408972	0.3241985935149325	0.2129642476378955
0.3255511498311148	0.3813360892262276	0.1587833702329721
0.3811885122398597	0.3257951202555027	0.1587547056067035
0.4373940383428442	0.3815432860572742	0.1553018208291725
0.4935592215458613	0.3258193514922508	0.1587027548743970
0.5506238952866046	0.3243274080784325	0.2130270119323998
0.5491174526417382	0.3814130070347251	0.1586386318300786
0.6063393487277119	0.3797923899536864	0.2132454281576024
0.2151209512558560	0.4374811452754624	0.2156727664423506
0.2706399242026695	0.4375220831584390	0.1607568974990890
0.2687356819327436	0.4951795629813743	0.2133248511554676
0.3255219813575356	0.4376746886341085	0.2129188900728722
0.3256566848986625	0.4936373188655996	0.1584560663657483
0.3813701391144382	0.4374997211090640	0.1552408237247241
0.4373503843363848	0.4934159562326513	0.1550604731245144
0.4933694691330108	0.4374472050636838	0.1552040613244402
0.5490955850979189	0.4936357974950947	0.1585067754555418

0.6040943834058253	0.4375046271526686	0.1608507142928654
0.6062063065481885	0.4952779646845965	0.2132351807935501
0.6598673121313894	0.4376108929454320	0.2155416746454962
0.3246210807913517	0.5503280502138224	0.2133285246541107
0.3812925678660299	0.5491171206117587	0.1583493984188491
0.3798303290692329	0.6059866637994155	0.2134189610581422
0.4373413539468203	0.6041977513132065	0.1605398577971805
0.4934779824283489	0.5492038469422064	0.1585197824619683
0.4949229656770659	0.6060279549009979	0.2134288610553229
0.5503066159748750	0.5504153651226583	0.2132062395364718
0.4374279555942624	0.6593185344914270	0.2159363334530361
0.3816371786316959	0.1588664155925111	0.3270183076850839
0.4373473793783820	0.1604467398021562	0.2715972653448737
0.4931666804844738	0.1586286025548764	0.3267789370710295
0.2696365853744906	0.2696469102611633	0.3255835314132063
0.3249079047072352	0.2133299794675835	0.3253465085505254
0.3248225024572232	0.2692216827629162	0.2696033688657935
0.3797248774474933	0.2130026358296108	0.2691957877867259
0.3813774797350889	0.2699524167977458	0.3265013921439441
0.4950422759576243	0.2128697995171745	0.2690993271057268
0.4933351990159139	0.2693357363574387	0.3259063906041390
0.5501986420176997	0.2129583225809914	0.3251173939954806
0.5501887179350461	0.2689277956584459	0.2692840353034056
0.6058850217395375	0.2691105534589879	0.3252234495525915
0.1591621832685590	0.3818786385412341	0.3273495437621905
0.2136822042719864	0.3253562397574180	0.3256326205251788
0.2134019109209408	0.3799962360818538	0.2695787875687701
0.2693494283807406	0.3251022647747779	0.2698175463316295
0.2702313730714175	0.3822150259701959	0.3269106570685967
0.3812471883604628	0.3256378508033586	0.2691784179163837
0.3816785971580887	0.3820706259270756	0.3266182212050768
0.4373305611403167	0.3822628644352767	0.2695361520371191
0.4934161794655312	0.3256208034371043	0.2693368776753205
0.4926583811503752	0.3822704572690789	0.3266092225138646
0.5492410968675090	0.3816439261256568	0.2694174671841534
0.6059903966890103	0.3247749857731901	0.2692833794630738
0.6055728196850101	0.3816816291414448	0.3259214648079671
0.6620206797845681	0.3248046544559619	0.3250926634276385
0.6620127777029152	0.3798761939248236	0.2690809271942203
0.7163423021885121	0.3818562366683515	0.3267947781962270
0.1610640399257338	0.4374085365178607	0.2721854874826318
0.1591153016623638	0.4930790953631381	0.3272777152249910
0.2132335683268835	0.4950062722112361	0.2693952130311449
0.2698957702183546	0.4930827419242265	0.3265605459905542
0.3260511654494275	0.4377256810177015	0.3266544963186462
0.3819686654656873	0.4381693205124478	0.2698147778460492
0.4374269314823845	0.4934451130837565	0.2699022594844243
0.4928681072106620	0.4381476632118908	0.2697758785434474
0.5486419866186126	0.4936778630229409	0.2699044734900546
0.6051486164651529	0.4935659823929539	0.3263396884716418
0.6619389165686478	0.4951925304729416	0.2691072636144980
0.7144041848451606	0.4375963681866605	0.2715254704330138
0.7161415235702503	0.4932891777038129	0.3269643484815341
0.2135108650128069	0.5497668091118338	0.3255207668784381
0.2693114252417800	0.5499207263686392	0.2696645200012347
0.2696963194301953	0.6052505428894427	0.3256361400748054
0.3250534873016344	0.6054891539879569	0.2696819145566316
0.3819661536960972	0.5487963801589877	0.2702512394028667
0.3817091283498106	0.6048691458051332	0.3265419912796328
0.4928433980657210	0.5488443636446639	0.2701879044629590
0.4930721328904590	0.6047796356042124	0.3265804771216669
0.5497652997958866	0.6053948497423179	0.2697014315601796
0.6056211577689732	0.5500093295320129	0.2695186669584329
0.6052875807574430	0.6052291266283504	0.3255004806665980
0.6616506563379191	0.5500214919025944	0.3252384280740527
0.3251630082975139	0.6614249251867323	0.3255472786842661
0.3798707837675992	0.6615113514028583	0.2694814184597512
0.3819605722796753	0.7157351303667775	0.3273550172821082
0.4375001099912775	0.7136524310068632	0.2721720018236959
0.4950042050018649	0.6614558993756754	0.2694908794365395

0.4930525858672070	0.7157602892323101	0.3272803380642911
0.5497301546996184	0.6613093065410650	0.3254635843673754
0.2717137423491005	0.1615434724620130	0.4382640854629654
0.3264188559360086	0.1590986412816807	0.3825870798946767
0.3818835361390038	0.1556159158685515	0.4384015801626799
0.4373228258207669	0.1558755415773171	0.3828277241491722
0.4928055461184034	0.1559362464277214	0.4383905052471708
0.5486594061327074	0.1586984734537267	0.3823732956889683
0.6038111915895850	0.1608250250839081	0.4381193254966074
0.1618152916788501	0.2718536322221359	0.4381938451413712
0.2159922485044523	0.2159618573359506	0.4382284896070522
0.2138803836130584	0.2695194029414572	0.3807436930588070
0.2694102657344102	0.2137544910322552	0.3807276953018852
0.3266947656535055	0.2141089573452481	0.4386209179079952
0.4371472058159517	0.2695808161699867	0.3825564943971264
0.4927832853903554	0.2698899852329324	0.4385186530678413
0.5491568854103971	0.2694850687948115	0.3820842360204590
0.6060823216171799	0.2131469309745530	0.3805098875126743
0.6595872683749430	0.2154403449086590	0.4380210670920885
0.6618016491860074	0.2689985385898322	0.3805400197085557
0.7141007555522569	0.2713094348493537	0.4380946550184718
0.1595074521172613	0.3268328370683403	0.3827884938631767
0.1559667435194299	0.3823354793774605	0.4384090589119287
0.2702113057453149	0.3264002155897285	0.3824640532756716
0.6055009525191678	0.3258969507829725	0.3820788641055269
0.7162443434373763	0.3264257168503149	0.3823767008401226
0.7190464382613376	0.3822689127954895	0.4383960153500699
0.1558198034636024	0.4375194454844818	0.3829285292457796
0.1558625197894644	0.4927688613747531	0.4384058359712784
0.7191807058760297	0.4376955382840242	0.3828138184364125
0.7194266974754923	0.4931885956897548	0.4383597206468919
0.1593639900011587	0.5482460982214469	0.3827674971712068
0.1615974577644972	0.6032805956865288	0.4382230969168419
0.2138356921082551	0.6054999455267973	0.3807446113126436
0.2702001387310622	0.5485000021136083	0.3824360236280457
0.5485976678203638	0.6046740787062203	0.3823223533194388
0.6046840031346242	0.5485715795089251	0.3824495927486578
0.6612200212763840	0.6055155464239544	0.3806078498152392
0.7158851840979529	0.5485899922911007	0.3824660486455174
0.7134623118362491	0.6032725815936518	0.4381606878517623
0.2159255906793834	0.6591271721783468	0.4380800938988543
0.2695122364030972	0.6612416411937295	0.3807780417818115
0.2718396810031720	0.7134184135246068	0.4382384582572198
0.3269069326382448	0.6607017716146131	0.4385710309469945
0.3268853755501241	0.7155816334811750	0.3827923827002362
0.3823832748025215	0.7189772090656300	0.4383708629109593
0.4374992808619800	0.7190157978013625	0.3829675695893232
0.4926682959450432	0.7189206399484062	0.4383297350532308
0.5481603839312040	0.7154954252607210	0.3826411262847282
0.6054599426353775	0.6611074026386398	0.3806392255022881
0.6031592737714965	0.7132324469257422	0.4380941068948295
0.6589926827804176	0.6589663257974655	0.4380463668508909
0.3265865269041002	0.1591772405202512	0.4938710374976841
0.3817742487609559	0.1591271780358202	0.5490416515774242
0.4372362262921112	0.1554768884476344	0.4937925411409912
0.4928474956023841	0.1589279581921265	0.5491499631183107
0.5485080063601208	0.1590935116302716	0.4939964883885277
0.2147027741586872	0.2703594547608312	0.4951461914377362
0.2702984102616692	0.2146883820655222	0.4951444408251944
0.2707507485128522	0.2706885705471548	0.5494040057767440
0.3261398339880208	0.2152952663580120	0.5492671138178142
0.5492206190189112	0.2142267952709426	0.5500544098276944
0.5486472547974856	0.2701361867190339	0.4942912930909680
0.6059956059770676	0.2133322489512216	0.4957088548204128
0.6049760386271833	0.2699938287469503	0.5499240726901879
0.6616445878461571	0.2690121225900076	0.4957309810313628
0.1593237887510040	0.3268618268416033	0.4936753070600026
0.1592969324542064	0.3819946655691675	0.5487269103244046
0.2152466101223305	0.3262858711391649	0.5490654651872280
0.6048519537223452	0.3263979692352117	0.4942984496719111

0.6607639418442885	0.3257678581686971	0.5500702473832705
0.7158447062128915	0.3265596169672402	0.4939876103663696
0.7160513133247295	0.3821592281281881	0.5491274285173118
0.1559408635032898	0.4375186613828475	0.4935947080835728
0.1593672109650477	0.4930224180899707	0.5487020142014506
0.7195545469336273	0.4378041781239521	0.4937638446740744
0.7159652005247388	0.4932005593660633	0.5490054292622052
0.1592503807662277	0.5482628772990988	0.4936556162260996
0.2152155942156510	0.5488707491110166	0.5491061304695838
0.2147329979653618	0.6046513087960897	0.4950020313403141
0.2707473098213858	0.6043329825355358	0.5493697480868224
0.6043276657060764	0.6043162776062773	0.5494349142698648
0.6597809577546920	0.5489023829042532	0.5492992486139886
0.6602988800857855	0.6046953984778547	0.4950789718902158
0.7158734951088581	0.5484967000096265	0.4938734565116461
0.2704037234301797	0.6603228369513078	0.4950295354837251
0.3262198640159330	0.6598238091838330	0.5491236458927693
0.3268653618538978	0.7157545231640383	0.4936528037761732
0.3819822643965030	0.7157067862457395	0.5487715173475594
0.4375147291968763	0.7190164413995120	0.4935306909743666
0.4930153258630672	0.7157854820570422	0.5487652331839713
0.5487248415723908	0.6598398045418827	0.5491154862444733
0.5481693425434732	0.7157105317144149	0.4936500700392353
0.6046395784563425	0.6603275673342476	0.4950876485643576
0.4373042510161789	0.1616122447308554	0.6037824928431176
0.3258495771018790	0.2708794902526890	0.6045174052434893
0.3806769486781497	0.2152386666652330	0.6046962825376436
0.3802285274716729	0.2706407544656100	0.6600262948632430
0.4373454709198428	0.2174817395751109	0.6579900514737882
0.4939632733773185	0.2150881204576295	0.6046499940920901
0.4945078789532800	0.2706846336554944	0.6598226004919613
0.5489099969429914	0.2709079298670753	0.6040284674963005
0.2151460717063720	0.3808934944171560	0.6045082724306755
0.2709131717347604	0.3260564530276413	0.6043438951346087
0.2704351135660905	0.3803977143132422	0.6599277950119716
0.3260232922582442	0.3262214434924323	0.6597638507691280
0.5488057195963699	0.3261519664732500	0.6594578192989453
0.6040770988496239	0.3260676881967343	0.6040274482302355
0.6043400434026841	0.3804352125649731	0.6598568096127339
0.6599477454078277	0.3809877227796961	0.6046811873962751
0.1617933552529016	0.4375053513838247	0.6033960623738258
0.2171971897659940	0.4375538037887034	0.6579429628476947
0.2151233085493192	0.4942032185478788	0.6045029495709665
0.2704129373608682	0.4948033845901914	0.6599807934703456
0.6044400503236796	0.4947400188198820	0.6600251608896658
0.6576157128270794	0.4375926152242703	0.6580438400706886
0.6598535091601638	0.4942748675882718	0.6047010157797064
0.7134400746829824	0.4376439190441794	0.6038206493132855
0.2708688566658374	0.5491245454751715	0.6044346757433862
0.3259990212525867	0.5490233890986335	0.6598155389444328
0.3259699127892288	0.6041723581497962	0.6044267111216058
0.3801971720395980	0.6046428271010829	0.6600308960284945
0.4945670156170089	0.6046096481777514	0.6599479510245599
0.5488774008918629	0.5490417004560282	0.6597919593294574
0.5489403336551377	0.6041788656680005	0.6044020453658118
0.6042196481868634	0.5492311810325071	0.6045795174899783
0.3807641017142058	0.6600214572380639	0.6045943354795401
0.4374277056778321	0.6578566010710598	0.6580326585632025
0.4374750164176024	0.7133545435976303	0.6035177227542290
0.4940948538662887	0.6600107453685635	0.6045989716853662
0.4374422887062887	0.2734266508179209	0.7122144757860265
0.3277197475687670	0.3829152265351483	0.7138848454010063
0.3827110726393513	0.3280745649632864	0.7139915444865496
0.4374765123136795	0.3829440596785973	0.7152410318781670
0.4921625340985427	0.3279515902791819	0.7137550370175941
0.5470566274552889	0.3827785308347939	0.7137358912026013
0.2731607842377392	0.4376049447611534	0.7121389081262582
0.3278122304513696	0.4922572219413159	0.7138950699034722
0.3827275211814289	0.4375889188139765	0.7152851297024921
0.4374557929720026	0.4922981567252391	0.7152558923518505

0.4921631073877065	0.4375648429180520	0.7152595456423003
0.5470197532626239	0.4923152189756871	0.7139165251805379
0.6015705137906709	0.4374922367857326	0.7122044002322553
0.3827230073455434	0.5471733457053064	0.7139184074616477
0.4373648954932694	0.6018281045364157	0.7121979073390658
0.4921136895782405	0.5472861182541223	0.7138559429423331
0.4925917668212548	0.3262928820678717	0.3825950347171965
0.5485493295941580	0.3265224819445511	0.4385880296680517
0.5486207606492904	0.3824520735384107	0.3826394141573949
0.6051128503905386	0.3823686735301531	0.4384896338652966
0.6053306090628429	0.4380750905373887	0.3824830960645519
0.4372739870156598	0.4391526213459174	0.3293237731473368
0.4373398964033179	0.5477925133688261	0.3295517222644216
0.3833383127933361	0.4933826430861481	0.3294957549722837
0.4913957095781321	0.4933470437014582	0.3293761134383455
0.2720893377980846	0.4379829804168485	0.3846706461668272
0.3269907594450796	0.3828889964806697	0.3853827156436956
0.4365365805531132	0.3275519558839882	0.4395552767152269
0.3276411698438342	0.4921886855336089	0.3837664755565511
0.3821652126994122	0.4379986108829056	0.3839796484168716
0.4373138403387986	0.4376700501304704	0.4379328394525953
0.4918705401356716	0.4390004160921703	0.3839936280916592
0.5475516734340790	0.4386097709754370	0.4392946869955287
0.4373487702469505	0.5490902955809450	0.4380144607208203
0.4918693833150957	0.5474916264769919	0.3843448843771920
0.5468948098289039	0.5474460685375523	0.4393058718510102
0.3275707017189997	0.2734180901281786	0.4933645478786287
0.3281396549355760	0.3283026075538586	0.5476343710134185
0.3267101277081466	0.3824026134367127	0.4936573975242499
0.4373583902119971	0.3270488569290756	0.5489287116933856
0.4917380219561120	0.3278913492750771	0.4945641495150490
0.5464932894032239	0.3285578924651140	0.5482207259756232
0.3268093184184431	0.4930138492196829	0.4935085871014275
0.3823633898513203	0.4377715667489423	0.4933692115949215
0.4371915574601086	0.4377759091545622	0.5484360358905312
0.4927282139904463	0.4374486151790756	0.4930911796013734
0.5480379640421446	0.4376430256905744	0.5489721608737010
0.3284213989834547	0.5467762354922200	0.5474825991907354
0.3274940346961506	0.6015740615931109	0.4935671856778784
0.4373066541170025	0.5486236831007126	0.5490691823235018
0.4928461993198704	0.5486197897033014	0.4937579247939599
0.5466516885300851	0.5469215520489371	0.5476942863291731
0.4374140441226023	0.2739059208654378	0.6034764455469781
0.3283931824744232	0.3837145003562840	0.6025470426459639
0.3823313364241576	0.3826598086983791	0.6578343671507708
0.4372445492771130	0.3274837509374874	0.6578148609267580
0.4923966171065009	0.3826449965073644	0.6576775460160652
0.5463526300928704	0.3837904238963828	0.6025876129522123
0.2736296265116238	0.4376555690559039	0.6037315245815845
0.3284569176591072	0.4917055586088386	0.6027333037257435
0.3825452438832825	0.4924830041165509	0.6578607175209560
0.4375196063376143	0.4375461357554141	0.6571662232174359
0.4924192101252993	0.4927042213678920	0.6577924673132007
0.5475475236623598	0.4377165546223801	0.6577611857691354
0.5464870555378458	0.4917486813042802	0.6026948520292449
0.4373339522236619	0.5479417193038737	0.6577483554529616
0.4373686396219181	0.6014960293267017	0.6036845498591381
0.3814609415283348	0.2729689222372831	0.4393694779122659
0.2728189670766444	0.3826090256833953	0.4386625044913659
0.3274072708777880	0.3281791326693379	0.4395058169955502
0.3822546363328865	0.3275359146482127	0.3853332383435604
0.3822828851139330	0.3824158423955401	0.4385631912169300
0.4363635268066604	0.3832434085418060	0.3838896945261617
0.4911739272833374	0.3835999176898903	0.4389352561584907
0.2727518450026126	0.4926039228164676	0.4388675445784740
0.3263979212386167	0.4379726972594456	0.4381386673971202
0.3820606434252478	0.4930016519547500	0.4380509294183411
0.4376948894844270	0.4937145762129629	0.3834162090605053
0.4926746012201313	0.4929661797244171	0.4383818415537526
0.5468612295485675	0.4927814285156605	0.3843353730642138

0.6020546969745175	0.4934499487302664	0.4395940461720261
0.3277940685667689	0.5468425796082607	0.4391824094289312
0.3829864588643768	0.5477203706253352	0.3841908726823812
0.3821020471776375	0.6021363605793372	0.4386350643704693
0.4374959376922379	0.6020857892149891	0.3832871220016099
0.4926843624568928	0.6023263024843027	0.4385776337181015
0.3825165386467146	0.2737487082422347	0.5486628641181653
0.4365549778084073	0.2732704374425384	0.4944871112061933
0.4913546104260801	0.2733948368137685	0.5490143045145439
0.2735024300900755	0.3275554867498836	0.4938013907936333
0.2735677099030554	0.3826120277832188	0.5486143940203063
0.3817940837879854	0.3269784901804363	0.4933383159370920
0.3820459325620174	0.3826644660894965	0.5487309027269029
0.4375533100823395	0.3823685583291199	0.4930124997517218
0.4925107964288000	0.3825725456643903	0.5484461392622854
0.5472446800722416	0.3834627409715716	0.4945062605105214
0.6016492561529868	0.3836550937549667	0.5490840614183977
0.2732773933424073	0.4375214382566970	0.4938833577656627
0.2735512447233061	0.4927665289794284	0.5486485057256550
0.3263708246260239	0.4377714357830801	0.5491223190856894
0.3823291071259731	0.4928634112292509	0.5485348716522597
0.4373024939705804	0.4927310563029508	0.4933309016669665
0.4924331128231275	0.4930691267141091	0.5486904074618977
0.5480555998271192	0.4933475584769699	0.4933462624936506
0.6017871151971015	0.4386280482777645	0.4945520498936109
0.6013367471552841	0.4925964379659360	0.5487195074084985
0.2735440284394024	0.5476513425451349	0.4936198947020906
0.3820065227168870	0.5483705060991370	0.4935248117060415
0.3824610147364198	0.6016132230602103	0.5486558514231240
0.4374842164146057	0.6018244994718190	0.4939979647224985
0.4923925189460372	0.6016075137065527	0.5487057317246585
0.5474660686394461	0.6016515315874523	0.4937323292438717
0.6014954305416441	0.5476166416968771	0.4934595675509230
0.3833289697447648	0.3286056401133925	0.6026971075880767
0.4371446758821649	0.3821611714828917	0.6043957093645957
0.4912337835764752	0.3286496373242926	0.6025749443183530
0.3271274528368255	0.4376487359994818	0.6577199744397587
0.3819957017572930	0.4377350858761692	0.6041104627817568
0.4373702139122824	0.4930771123228854	0.6041225672279721
0.4929060615262348	0.4378947631235404	0.6043502062613686
0.6012363542650032	0.4375580521218426	0.6035515613291534
0.3834508923933593	0.5466085016440907	0.6027863575816276
0.4913377783524464	0.5466817747031868	0.6026188814277207

$Cu_{303}Co_{102}$

Topology (346,24,60,24,94; 436), E^{PBE} = -1702.5273 eV, magnetic moment = 162.95 μ_B
$Cu_{303}Co_{102}$g300fENC400-1702.5273m162.95

```
Cu   Co   Cu303Co102
   1.00000000000000
    32.0000000000000000    0.0000000000000000    0.0000000000000000
     0.0000000000000000   32.0000000000000000    0.0000000000000000
     0.0000000000000000    0.0000000000000000   32.0000000000000000
   Cu   Co
   303   102
Direct
  0.3813434313623746  0.3815487269337555  0.2121685851077799
  0.4371757682219961  0.3253666586839806  0.2128789456559134
  0.4931648711696890  0.3814982680617298  0.2121436400187225
  0.3812795781886316  0.4933391697654890  0.2121194843646868
  0.4371494902905610  0.4373761828407219  0.2112885224503386
  0.4933361481980523  0.4933919638474827  0.2119169270391032
  0.5496560215976218  0.4374232274481224  0.2125310304423776
  0.4372814114195472  0.5497486196191790  0.2126011337966735
  0.4372054222681073  0.2129432531070498  0.3262118400654189
  0.4370767141300838  0.2695637747760620  0.2699199991684249
  0.3256939639474364  0.3260982059919053  0.3265331874890379
  0.3255292077481788  0.3817699754948603  0.2704262727513284
  0.4370451110832982  0.3261138532773136  0.3264251208754818
  0.5495682266387257  0.3254375223643119  0.3256406762368470
  0.2143402601216183  0.4379109415531249  0.3267594068402453
  0.2702988405134085  0.4372575115701195  0.2705046941523930
  0.3255391824793600  0.4931131675319081  0.2698390183422713
  0.4372749633511069  0.4376972770253088  0.3264606213446238
  0.5490292808148497  0.4376131442397503  0.3260270660827358
  0.6056642412973724  0.4375103589917386  0.2695354344871557
  0.6620856519958717  0.4376060076044281  0.3262551908939894
  0.3261168546332853  0.5492168730227045  0.3262743609996134
  0.4370841242942473  0.5490202200259779  0.3268530556378973
  0.4372869269438226  0.6054561779724401  0.2698585999776097
  0.5487508695900986  0.5493024761675651  0.3262746635146092
  0.4373476453879224  0.6617859056770447  0.3263782983404375
  0.2706969135269857  0.2706099836463079  0.4382230947466777
  0.3257639914466190  0.2703045724421325  0.3825897415376653
  0.3814981015692270  0.2123023322209248  0.3823980322511191
  0.4371539038889365  0.2115216882164686  0.4382443188232826
  0.4930332109097729  0.2123726225870300  0.3823704561250906
  0.5491213948074192  0.2130204694279390  0.4382699997301018
  0.6051356180762461  0.2697439279847155  0.4383434005197416
  0.6616864655807089  0.3259763912189435  0.4384788716001453
  0.6624894337965643  0.3820182371120048  0.3825826951837605
  0.2141914850345575  0.4930455493578068  0.3827390138293058
  0.6625176760284838  0.4381061065747116  0.4389853738605229
  0.6618927060344300  0.4932367727865904  0.3831158902889270
  0.2146763582322543  0.5485319059457892  0.4382230900971849
  0.2706175863227139  0.6045703970027547  0.4381787379630004
  0.3260091862508157  0.6050991180759311  0.3825081783263790
  0.6043984247290486  0.6044057924901861  0.4380960777825329
  0.6610398933241128  0.5485104210144911  0.4388384228680186
  0.3819430116323695  0.6618056318194421  0.3831393446244799
  0.4372687830360001  0.6617844503705068  0.4388058099660316
  0.4927216883930328  0.6617320780129907  0.3831846950670872
  0.5481298456767706  0.6608788803969656  0.4387256076644445
  0.3815620539542314  0.2123760357625229  0.4937649472830363
  0.4372260700228606  0.2129782059314596  0.5494549914161481
  0.4930242042320878  0.2123916646579257  0.4938590675079875
  0.6611525701305419  0.3826701906355501  0.4945605898626141
  0.6602977039051082  0.4378704193041048  0.5493956232071209
  0.6615715601380771  0.4932129843750447  0.4942313604388353
  0.3821258743279133  0.6609522671585443  0.4937239181148043
  0.4372943975771414  0.6602599822841873  0.5489239666828133
  0.4924906362052694  0.6609398154908096  0.4937490573533423
```

0.3794802539311511	0.2686156708945787	0.2133563861083882
0.4371829261598748	0.2150168290436815	0.2155743174608147
0.4371542851876327	0.2705921314526321	0.1607025900220722
0.4949832043799222	0.2684356325958400	0.2131207322808149
0.2686478421366581	0.3797372609426418	0.2133594663784892
0.3242286270859486	0.3244669325019046	0.2133691610167302
0.3254776559015813	0.3812917135483991	0.1586341970896253
0.3811206246405880	0.3255831961390065	0.1585853854186018
0.4372125403654302	0.3813846343554980	0.1552904322208024
0.4933794285970966	0.3256220226252317	0.1585440273285033
0.5506795712356120	0.3240974193781562	0.2127983901082720
0.5492198923307882	0.3812035437707922	0.1585637173252179
0.6066755009084964	0.3795438278632394	0.2128145633262488
0.2152765895341394	0.4373618646503156	0.2160536125854531
0.2704602354797669	0.4374548438904483	0.1607657177209983
0.2685195883491711	0.4952140097812641	0.2132864121007184
0.3253865009810675	0.4374798464575249	0.2127387560347003
0.3254466450227978	0.4936149032113784	0.1585087327279907
0.3813017300586246	0.4374585601653783	0.1552317761587398
0.4372125678732216	0.4934875823619295	0.1553526074613573
0.4932086974597206	0.4373439846265630	0.1553066717033106
0.5492875274822723	0.4935684801294948	0.1584746565184093
0.6044101733883218	0.4373259884839111	0.1603279145998653
0.6066622619800495	0.4952842447392249	0.2128085891462664
0.6601062472782427	0.4374842269042911	0.2152564942471966
0.3240555099426694	0.5506785379310557	0.2130007760824588
0.3810627758412079	0.5493617664015557	0.1585959159989000
0.3793991653750078	0.6067121844735226	0.2129615351383112
0.4372297932512589	0.6045279352001153	0.1605143523992017
0.4934565762626910	0.5494181427918241	0.1585073775226512
0.4950321644616383	0.6066278524483442	0.2130363649635584
0.5507246197662145	0.5507861554300276	0.2127106702617657
0.4372660468626061	0.6600373502672188	0.2155715801413958
0.3812878858277814	0.1585819045909724	0.3265651186720332
0.4372177476076327	0.1605701483960564	0.2715321204013569
0.4932859193224475	0.1586069610359563	0.3265998093117204
0.2698934039635649	0.2700703909786270	0.3262080147133862
0.3244630223027276	0.2131063010497916	0.3252136471911551
0.3245840911149491	0.2692132811120984	0.2696741660712471
0.3794338015561152	0.2129843191288007	0.2691850466436136
0.3810581920149797	0.2697796369341143	0.3265636398443127
0.4950046542999625	0.2128544624965931	0.2690051590146046
0.4933589590053259	0.2692051080623424	0.3258880035992006
0.5504934523043999	0.2127123604604791	0.3248071127217592
0.5503394821956059	0.2687033276237754	0.2691042147912870
0.6061101220995374	0.2688628036271457	0.3250942613274782
0.1605307447792753	0.3825080197766709	0.3283903170796664
0.2147489206770544	0.3259764663221104	0.3263773755543761
0.2143139128144499	0.3804193170759929	0.2704645044201623
0.2696026376587997	0.3256713265238164	0.2703968607642075
0.3809722202750936	0.3260053702407428	0.2699472285512873
0.4366966510271417	0.3815862263914053	0.2694151055404207
0.4934744122750185	0.3253807529369325	0.2692367603657106
0.4930223614938760	0.3820266162856059	0.3262734570085906
0.5496910282900379	0.3812560542858686	0.2690217740264176
0.6063233902799094	0.3244750792073764	0.2689895391608264
0.6059000558173455	0.3814751452118371	0.3258330963026456
0.6622212478588186	0.3245243682856747	0.3249435661928957
0.6622459155549141	0.3796835812234988	0.2688657967077056
0.7162902628823370	0.3816609581252556	0.3266968905435454
0.1611471250825086	0.4372847208205099	0.2722602831540039
0.1593119773858289	0.4928307686386103	0.3274604711168652
0.2134814204220190	0.4950021526956330	0.2695041144143556
0.2704029708611644	0.4933430252891474	0.3264815733612008
0.3809363529489163	0.4371316628542429	0.2697875145559200
0.3816139915774646	0.4932683149896318	0.3266284258251849
0.4371852938896331	0.4934847800473973	0.2687948936643642
0.4933565470937750	0.4375213860433481	0.2687062311217395
0.4928666699445734	0.4933187313231904	0.3266814722350926
0.5495773402930536	0.4938086040244802	0.2690654069448205

0.6058059271664031	0.4937128002275987	0.3256744383555721
0.6621818304307541	0.4953429829763051	0.2688099253816573
0.7144799287904436	0.4375295882105253	0.2715873763378299
0.7160440460280618	0.4934508390917700	0.3269353830947929
0.2136421005322962	0.5498911390283240	0.3255054850253379
0.2691142198490454	0.5501833579140158	0.2694538954777899
0.2695277377195097	0.6055600777008161	0.3254512768898363
0.3246128021937011	0.6060971400681361	0.2693049143566549
0.3811740040982189	0.5496359722132496	0.2691220932919788
0.3813040224955290	0.6052182348823235	0.3264610041149928
0.4933940242875703	0.5495497439268529	0.2691765380486624
0.4932038231383291	0.6049935622202384	0.3265646513266586
0.5500296068598134	0.6059585603138643	0.2693701773127101
0.6059930673609757	0.5504102004792879	0.2691040112667080
0.6054121328716499	0.6055086602905861	0.3254632306101483
0.6618326796903393	0.5503351143334434	0.3250783570190723
0.3248302377730048	0.6618621024228341	0.3252917940717036
0.3797066260295123	0.6620141485535870	0.2692340787281703
0.3817064839002432	0.7160882480274344	0.3270689277880189
0.4373698166523543	0.7144690673519748	0.2716085575313666
0.4948372165351685	0.6618556293960736	0.2693538946944620
0.4930057420130857	0.7160734402680509	0.3271600869377521
0.5497557867205746	0.6615737306654588	0.3254984479262742
0.2708216827303765	0.1604775558110823	0.4379868825013788
0.3258322854769943	0.1583211860659071	0.3821935022036552
0.3815766010828216	0.1551218088004113	0.4380399865831696
0.4373583615794909	0.1553760203344866	0.3822615658618875
0.4930767388532786	0.1553251040051139	0.4380751965164555
0.5488721665303632	0.1586535250976601	0.3821884921030602
0.6040487711193726	0.1607635026871220	0.4381802460701915
0.1630291945716525	0.2732808919795490	0.4382075123241819
0.2159083058021619	0.2157881073361940	0.4379883227358162
0.2151626914657503	0.2703764425828900	0.3813899516229895
0.2690584387417417	0.2130955777273185	0.3805159055884666
0.3256830210317470	0.2127987934700707	0.4381821747863989
0.4368044629564702	0.2696605293603814	0.3825246098625362
0.4927514322919982	0.2697921904159749	0.4383843660950000
0.5493440568739886	0.2692260763462657	0.3819598428473798
0.6062911177645718	0.2130093955728533	0.3804192392344207
0.6596638169786487	0.2154564670940836	0.4383155772744675
0.6620189012943738	0.2687792385744970	0.3805671133933760
0.7141856528705476	0.2713159114008775	0.4384294581621742
0.1608680374981859	0.3280315559400282	0.3834705977387559
0.1591686426705421	0.3829107215545754	0.4386029609005121
0.6057419069964094	0.3255680916100724	0.3819397909492068
0.7162930267110088	0.3262618724245305	0.3824797779248981
0.7188146601442590	0.3820409377875210	0.4387535652098649
0.1582801796842619	0.4374014885952897	0.3836702371644229
0.1582915372366544	0.4922614831248834	0.4388421051006421
0.7191633328541764	0.4376621671066497	0.3828539595377115
0.7194508169743606	0.4933474420113020	0.4385379533516067
0.1594249301886872	0.5479510780753403	0.3829851664108191
0.1615449837959505	0.6032100579855388	0.4381665343796207
0.2139383985277241	0.6055169221989625	0.3807368453171324
0.2706414156942754	0.5486972027572293	0.3825412289357795
0.5486956594681899	0.6048177759566419	0.3826061722970007
0.6050768091011303	0.5486568053119759	0.3826705490411346
0.6612613908343152	0.6057551300325196	0.3806684847567361
0.7158629556911309	0.5487723486791691	0.3825501963319248
0.7134808520190276	0.6034600266276491	0.4383059505614949
0.2157744937488426	0.6592156561157293	0.4381437652737316
0.2692698207413616	0.6615425189036642	0.3806563189985171
0.2715868785953944	0.7137266381994914	0.4382691694627211
0.3266011958516286	0.6610849054356032	0.4386920008274472
0.3265525031929403	0.7159809381084651	0.3827500705804147
0.3821930035941830	0.7192961997946118	0.4384911115774839
0.4373442813277488	0.7189651616270261	0.3828656679430224
0.4925758323109132	0.7191412321918480	0.4385659971782088
0.5481768547816470	0.7157959072156325	0.3827664749536419
0.6054916525663038	0.6613549565505534	0.3806912835163005

0.6031577251063494	0.7135289963935294	0.4382742610676344
0.6589507049593202	0.6591536192833882	0.4382479381426893
0.3260090168764458	0.1581749476943664	0.4938613021607013
0.3815618187580949	0.1582228129662277	0.5494677638232479
0.4373508002286801	0.1550609875314931	0.4938164145927672
0.4932239764490123	0.1584500474478792	0.5495853644090395
0.5489152640203623	0.1587127466364663	0.4940933238574483
0.2153086627895574	0.2700673482558091	0.4949942731153586
0.2693075065688180	0.2130487508751350	0.4956392512530275
0.2709059875012712	0.2704313393984787	0.5494138492351580
0.3248575325426165	0.2131186509814323	0.5505207392145203
0.5502504682680917	0.2131580380603953	0.5509599180564811
0.5486384766121974	0.2701296023428785	0.4945559951260528
0.6060163915563191	0.2133390006045022	0.4959762248099886
0.6047511801329705	0.2702651074204991	0.5507415392996420
0.6614476247946847	0.2690247423959399	0.4961504726255680
0.1617232490031178	0.3278304951786202	0.4931605239028919
0.1614537892090678	0.3826325255483690	0.5480236586812157
0.2154346907519800	0.3257330847048071	0.5497496566897276
0.6049161339665426	0.3262573897522159	0.4944078422163692
0.6605031936025623	0.3255763870012318	0.5510035383507255
0.7153615086885368	0.3263486344457342	0.4945335000843566
0.7158212483046579	0.3819258062440288	0.5495005833058134
0.1589966374313939	0.4376052500236580	0.4936008228422485
0.1606736065681640	0.4927453550995912	0.5481795485404974
0.7193068023981946	0.4378787513364673	0.4941873271357148
0.7158257018431584	0.4935756797424858	0.5496222388093506
0.1606366286560554	0.5475423499226366	0.4934753300732237
0.2151737445152775	0.5493041134460791	0.5495499103393150
0.2145782493698929	0.6050347996053050	0.4951185780940807
0.2705833794861113	0.6044886833879177	0.5492978048635473
0.6043317946963598	0.6046014037979242	0.5497241858893580
0.6599711155455030	0.5493198603902346	0.5497997371799904
0.6603456149025027	0.6049399053056808	0.4953547189013481
0.7160337496355744	0.5487702908410051	0.4942393266884816
0.2700775138979272	0.6606480990226001	0.4951715566863943
0.3258949048784248	0.6600102972253831	0.5491567711197182
0.3266452024850884	0.7160237750588246	0.4938088582639910
0.3817384145558767	0.7158635525568319	0.5489931441596296
0.4373278304582319	0.7192289358754773	0.4938398105283725
0.4928773088317260	0.7158310249047304	0.5489733562023992
0.5486908626737976	0.6598846063365252	0.5491817753165035
0.5480644822294820	0.7158269758259120	0.4938494704697137
0.6046201445980730	0.6604523684429771	0.4952323905249810
0.4374312177113783	0.1607471197334920	0.6045002729174943
0.3260802532615369	0.2706098373561295	0.6043534449006761
0.3798679663655621	0.2133002913583581	0.6059705282832790
0.3805162398095950	0.2703205379125375	0.6599663102813476
0.4373573456358442	0.2160828533045573	0.6593069906674860
0.4948410917372806	0.2135798972097086	0.6059782599963917
0.4940083612087873	0.2706241477205579	0.6597879109301681
0.5489890334363520	0.2711297518313237	0.6044499737660953
0.2154914706930150	0.3807073480039854	0.6048046861481881
0.2711964561911586	0.3261923133961501	0.6042090665347684
0.2707356658053650	0.3807504133354389	0.6599418326736993
0.3259149317434487	0.3260847227040767	0.6597678293040040
0.5487571467278380	0.3256795476115652	0.6601155243893359
0.6604041642767351	0.3806808669482564	0.6053036916133352
0.1627112074673258	0.4375367433102304	0.6023514051203778
0.2174157052172598	0.4375869368206170	0.6579345418044069
0.2150933104967761	0.4948195270411171	0.6050274551094733
0.2703469694382754	0.4948318792331565	0.6601145879766699
0.6044669178380938	0.4944732759902505	0.6600189167514504
0.6575862075638707	0.4370855708591823	0.6581525144595525
0.6596220619855562	0.4941165077794741	0.6047871587931897
0.7136099352821960	0.4377417714590616	0.6040627216458100
0.2711237493405935	0.5487807693778017	0.6039998097308042
0.3256710223373467	0.5491656789930041	0.6597162837113215
0.3256454664690846	0.6043410195067265	0.6042997704345506
0.3800305041350238	0.6046570028616898	0.6599594289070273

0.4944215366644798	0.6045563269964768	0.6599242023472783
0.5488074539612252	0.5488974116027643	0.6596141177094639
0.5488636610444206	0.6043025332077477	0.6044590133235334
0.6041477596778826	0.5491524581929434	0.6045888500950596
0.3805384134230519	0.6600621607807186	0.6045718681542467
0.4372132334626311	0.6577796069988758	0.6580017148500829
0.4372648081346634	0.7134223430827191	0.6036430618739181
0.4938820137619521	0.6599378170318442	0.6046111247085685
0.4373465502841131	0.2736103867479026	0.7121411596988408
0.3277540646520605	0.3830320736815910	0.7138607490823629
0.3825899328338355	0.3281563425088062	0.7136504019380467
0.4370813945501802	0.3833521377358206	0.7153934444865788
0.4918848566297200	0.3283332556547701	0.7138763866938634
0.5474224888115322	0.3825246846092119	0.7132789708204518
0.2730981497528028	0.4376704388303322	0.7125177276842586
0.3275183167552184	0.4926100421911655	0.7140665457167722
0.3823186212086305	0.4379618714987447	0.7154658904762412
0.4372674950050319	0.4924934780014198	0.7153622360222730
0.4921594364417802	0.4376946410647700	0.7151985815516337
0.5469833761965087	0.4922661049193308	0.7134555585365645
0.6019221936469715	0.4368695299277011	0.7121960801834782
0.3824948287228518	0.5472994973290830	0.7137807270464231
0.4372716813078270	0.6017924375784477	0.7122082209162359
0.4921293083832745	0.5473218623500086	0.7138418191392029
0.4930136992729000	0.3262576234496466	0.3824525039256973
0.5489381074864563	0.3262680880651620	0.4384090316500833
0.3260618099494547	0.2705854947387155	0.4937559876551352
0.4372296393444383	0.2708713473080327	0.6043717279110262
0.3813168816098630	0.2703072647897334	0.4381962977830295
0.5490271223140013	0.3822494856362631	0.3826658496565628
0.6052939533575331	0.3823592500461392	0.4385635319848920
0.5490185845116299	0.4930102230394269	0.3827082161643143
0.6056282508437065	0.4380754869407690	0.3826833490627764
0.4371449844253302	0.6053048888413051	0.3828148415096357
0.3816924128994862	0.2707423057423782	0.5490927879542726
0.4370947932802374	0.2704906621045898	0.4936691472982620
0.4928103552568258	0.2707072947979173	0.5491872322302336
0.2168935542332669	0.3283895119503776	0.4381710579511242
0.2168337365319419	0.3827690671461555	0.3841283584604278
0.2167916926873791	0.4374812581179049	0.4391820984234464
0.2175736548254101	0.3832935779713644	0.4933360397901090
0.2172118377275827	0.4381445927462795	0.5485279559822553
0.2167844058439972	0.4919084740996882	0.4939451840577203
0.2705958917237226	0.3827736198913101	0.3299752902049458
0.3810258190744387	0.3824320172963567	0.3298709141683855
0.3256451024919520	0.4361071113454176	0.3292771528529309
0.2706574460626830	0.3291605016893576	0.3839020787998681
0.2710448854357504	0.4368411535031218	0.3838797348843412
0.6012412331323665	0.3302935212190069	0.6025944114007602
0.6013480796941311	0.3826021185353181	0.6558326106323374
0.3258112452651621	0.3824785114697957	0.3834768818797783
0.4363771297168103	0.3285907143262617	0.4381440790487622
0.3271645215848066	0.4918519818732393	0.3838203492644114
0.3807658726683675	0.4371161590535825	0.3834562506561235
0.4370774292701527	0.4376636987620108	0.4380633355920068
0.4916111856725727	0.4372311237999754	0.3851258219075351
0.5470892832417623	0.4382081466254850	0.4395628255080435
0.4370866297805500	0.5486812290175601	0.4381867602735318
0.4922230678682945	0.5483475251471284	0.3856709440456833
0.5471604486268942	0.5476212343689845	0.4398537744836732
0.3271466917224506	0.3287654515884885	0.5481428911068450
0.3263569599365209	0.3821312273396080	0.4935837260962166
0.4374412867615036	0.3292742999420105	0.5493717792617433
0.4920751504922067	0.3286694538335034	0.4937627024505358
0.5472148692788827	0.3292120384647486	0.5489413258309154
0.3264798279390124	0.4931187009773312	0.4933401269712561
0.3820734425630342	0.4377285604291709	0.4933658845438434
0.4368842482240785	0.4379191330187330	0.5483895867364138
0.4923225692346439	0.4374784964007108	0.4931755942384631
0.5478631669117394	0.4377000797311604	0.5488442657375474

0.3279319850193921	0.5468992648790066	0.5472657470362328
0.3273520658511996	0.6017512383511457	0.4935450570216086
0.4371389531893496	0.5488224511594376	0.5489444199088974
0.4926219035240447	0.5485699603013033	0.4938717133107401
0.5467869036595024	0.5471912108336033	0.5477827825821709
0.3280700508811020	0.3841012593493503	0.6027110071431616
0.3821619193008538	0.3832271909190921	0.6574276658244831
0.4371657698496645	0.3286102296121263	0.6581225585770794
0.4920002856990396	0.3833000176203581	0.6578526872701553
0.5462535366414693	0.3831200092549349	0.6032897446972638
0.2727845451512939	0.4380860575612574	0.6026723506685122
0.3281205158993636	0.4914542643465442	0.6027262975587373
0.3821549574273190	0.4926391055307208	0.6577454873265369
0.4370451978225935	0.4378561726647524	0.6575106138985038
0.4919553446144330	0.4927846292422977	0.6578623403885596
0.5474556585524422	0.4374237779576222	0.6572503088096422
0.5466277563260494	0.4916962033256753	0.6027765434865170
0.4371866215196057	0.5480485942184287	0.6577134388423466
0.4370814990436833	0.6014667543845019	0.6035108955676113
0.2710209297374366	0.3828171176720950	0.4382813738053908
0.3255281493425402	0.3288786031273594	0.4380814583945029
0.3815304876565817	0.3287831260683528	0.3833156599475444
0.3815671197877871	0.3821931439051555	0.4382012008851556
0.4361549017710222	0.3831006961818179	0.3842476768658731
0.4913638273176334	0.3832204572433298	0.4396486505791461
0.2717068037152464	0.4919319693038475	0.4393321103435143
0.3262299165792464	0.4377470348366915	0.4375097890314439
0.3819354985015171	0.4928181883483906	0.4379929129562281
0.4375337557733748	0.4935142628538524	0.3850978254833333
0.4922699571003140	0.4927733671853609	0.4385685930258960
0.6030627858472650	0.4933080533414416	0.4410894173012229
0.3272993111433722	0.5472559856376682	0.4399776707004524
0.3813082101131143	0.5477471926199577	0.3852143134948033
0.3810450949489139	0.6019692562526284	0.4394448316888313
0.4931325959345940	0.6022831557525848	0.4396449101589113
0.2712660559323678	0.3290406450784856	0.4931129750786367
0.2721877049580655	0.3839135552909222	0.5473734867961172
0.3814174104040542	0.3287552446055123	0.4936153095589169
0.3819346215414558	0.3822457508378352	0.5486936912564382
0.4369455919672240	0.3819594316845470	0.4932299965820729
0.4920304117359078	0.3823297496129001	0.5480426685260325
0.5473220482823710	0.3835267401470757	0.4942667052192098
0.6014961238436889	0.3829980216630102	0.5497824111199021
0.2711634639093334	0.4380480831052735	0.4935578428607749
0.2724348393108268	0.4914758461602985	0.5472812621196542
0.3264499884868863	0.4378464399767313	0.5488478190203820
0.3821435597499589	0.4931026962159260	0.5488195338379602
0.4372293502166236	0.4929373998821323	0.4928735909390721
0.4925427656746036	0.4932506931024717	0.5487709647843881
0.5477548297464975	0.4931208655734682	0.4937407638186168
0.6022376943490977	0.4392588147406069	0.4944927214781804
0.6014396047147388	0.4927289224408389	0.5483448719641311
0.2722863238680139	0.5467642215448743	0.4935775307652688
0.3818562133839185	0.5482500933197381	0.4935860489888184
0.3822292317936426	0.6017199805048362	0.5485379492692208
0.4372167761958842	0.6020415978468995	0.4937347572393824
0.4922601883136496	0.6015864131353932	0.5487027310363336
0.5474338539807995	0.6015317704965683	0.4934525711560589
0.6018178216978587	0.5477397861318302	0.4940356286888097
0.3824812958102115	0.3292220411665228	0.6038487158453228
0.4372806074217505	0.3828615274263288	0.6040346437531491
0.4917742121220238	0.3293879123573705	0.6035586234813668
0.3270274303061752	0.4379512642126028	0.6579618170556891
0.3817674856743054	0.4379963517362081	0.6040419045372990
0.4371031517598744	0.4931364190413894	0.6040481096436549
0.4922680355547733	0.4384178343858905	0.6041944934044653
0.6008574933084464	0.4370366675934262	0.6033453317782891
0.3828645483759863	0.5468886976940909	0.6028320465921421
0.4912456575135036	0.5468799441512670	0.6025844192529389

$2CO(Cu)/Cu_{303}Co_{102}$

CO2Cu-Cu303Co102g2.18fin-1735.4637m163.76
E^{PBE} = -1735.4637 eV, magnetic moment = 163.76 μ_B

```
Cu Co C O  CO2Cu-Cu303Co102g2.18
   1.00000000000000
    32.0000000000000000    0.0000000000000000    0.0000000000000000
     0.0000000000000000   32.0000000000000000    0.0000000000000000
     0.0000000000000000    0.0000000000000000   32.0000000000000000
   Cu   Co   C    O
   303   102     2     2
Direct
  0.3817754560684502  0.3818725807525216  0.2122401362672040
  0.4375196287471217  0.3254840635257387  0.2129377923742371
  0.4933529446602255  0.3817974869890459  0.2123497761492113
  0.3818568847643803  0.4933264444192886  0.2120231538389662
  0.4375795389567985  0.4375614208291621  0.2112838827582233
  0.4932611643568425  0.4932891886471268  0.2121223843448416
  0.5494393386015208  0.4376174432939000  0.2129452289936477
  0.4374968316452049  0.5493474359354585  0.2127975038067348
  0.4373665936909269  0.2128093624923618  0.3259303525154721
  0.4373502544487977  0.2692509118805570  0.2695192776841929
  0.3261464735774491  0.3262586989608223  0.3265197991942571
  0.3256491409374710  0.3816273085691545  0.2691314740888481
  0.4369433224637578  0.3257834311610966  0.3261269453048160
  0.5496492409158769  0.3253847825917902  0.3255524139877462
  0.2138791309726195  0.4375748504692102  0.3267937236777508
  0.2697437928011018  0.4375926530087528  0.2696292393888269
  0.3262729734690407  0.4934926699886988  0.2699567673470988
  0.5489916314038219  0.4380458282931716  0.3262503425958986
  0.6055671421599785  0.4376998537583352  0.2695751170573483
  0.6621212218573025  0.4376247000591980  0.3258844660539236
  0.3260720772965289  0.5486280536136885  0.3264322061187787
  0.4374797324828654  0.6045419533079716  0.2705014369389513
  0.5488810022187606  0.5486183397555475  0.3264186646672650
  0.4374229607059069  0.6610057134004387  0.3268374927958051
  0.2704148670515786  0.2708470935651879  0.4379990287189279
  0.3260042315940394  0.2702497041808865  0.3823068521828358
  0.3816704501918083  0.2132351757744364  0.3827866985301536
  0.4368906865539414  0.2129178999809583  0.4385132750707296
  0.4931179508616934  0.2125317548805358  0.3822786207494372
  0.5492579408396732  0.2132392742640307  0.4381445865805307
  0.6054264041689202  0.2698423587249568  0.4380798346893904
  0.2142602518900555  0.3270990053521708  0.4385755532535264
  0.2136609923782209  0.3826943855982190  0.3829618554325875
  0.6618967335334811  0.3261275707577140  0.4382761935498519
  0.6625376534241492  0.3820993136912674  0.3823099384299758
  0.2132845594542917  0.4376401070602480  0.4384583403960594
  0.2134788187033546  0.4925507159948737  0.3829621077515221
  0.6621673320146242  0.4381786187481692  0.4386194186607467
  0.6619142114694055  0.4931869848978180  0.3827806106033290
  0.2141117720650745  0.5481210310755380  0.4385296678806451
  0.2704114326225550  0.6041950063178183  0.4378611282145931
  0.3260173193677053  0.6045890745281077  0.3822876842784337
  0.6043862342262503  0.6043180428548434  0.4378672631895749
  0.6609520434821583  0.5483913175580046  0.4385508139257125
  0.3822553352456993  0.6610412062762062  0.3828746232622427
  0.4373462316005509  0.6612054828986218  0.4380504996723792
  0.4925628868192480  0.6611544904105119  0.3828406062881709
  0.5482968887341648  0.6605985255928147  0.4383476106563293
  0.3819572508603992  0.2141460606485048  0.4935385931177335
  0.4370438992694635  0.2145299971909552  0.5486915773150923
  0.4924908149485824  0.2136566186627352  0.4938895203683726
  0.2142213607064004  0.3825839768305805  0.4936446427924021
  0.6613814033912252  0.3827863700460558  0.4941397148739041
  0.2149004832642162  0.4376529346961981  0.5488887734450049
  0.2142196237255740  0.4927275757885280  0.4936263985129961
  0.6603660654456326  0.4380596504628591  0.5490432849065456
```

0.6609828708497963	0.4930066052791025	0.4937525436386310
0.3821637810273108	0.6606263520066478	0.4931796714591932
0.4373965785714567	0.6603821720990992	0.5483315613575613
0.4925602707682603	0.6606826655160395	0.4931545221368960
0.3795210307313520	0.2681972883474492	0.2129670102774067
0.4374044397893999	0.2145593609121793	0.2155006324432976
0.4375512063894191	0.2706864804322388	0.1609117981961855
0.4953691071231843	0.2681990816602794	0.2131405177733341
0.2685305336593651	0.3797870556252245	0.2129564984761498
0.3243519249330194	0.3243402425546048	0.2128630882148121
0.3258086681219647	0.3814504284753183	0.1586534890182028
0.3814147882938095	0.3257278620780458	0.1587304242032289
0.4376085857830485	0.3814701201070121	0.1553515739584217
0.4937088506374747	0.3256687488872636	0.1587851978926319
0.5507294588121958	0.3242836094633080	0.2130371731793488
0.5492793270507617	0.3813746782965186	0.1586790225465986
0.6066147245033772	0.3797441322158132	0.2131258493392766
0.2151957868222484	0.4375124149008517	0.2153416601627409
0.2708309178210699	0.4375797029704549	0.1605187320987019
0.2688192288665037	0.4951880769069364	0.2130885420005016
0.3256526363930066	0.4377048098175466	0.2128158084651074
0.3259332639609291	0.4935754019748301	0.1582894628035805
0.3816216941442627	0.4374929705589728	0.1551443694415571
0.4375860912840583	0.4933928712004798	0.1550551548049536
0.4935838087269235	0.4373913383195862	0.1552383085335393
0.5491812854980137	0.4935904032618697	0.1584874043753688
0.6042102536257857	0.4374997296991943	0.1607577561394520
0.6063850823749719	0.4953007064623377	0.2131322908028482
0.6601060667546385	0.4376499690565235	0.2153115182092186
0.3248607976934476	0.5501788191665451	0.2132229657534369
0.3815623592030445	0.5490921340320831	0.1582971978673882
0.3799539996421339	0.6059458273395225	0.2134448247900142
0.4375134395238298	0.6042240296310771	0.1607085667818261
0.4935924201472104	0.5492235562159543	0.1585753272780766
0.4950298742145552	0.6060202262198454	0.2135533072786175
0.5503266912886945	0.5503226558706180	0.2132338760407681
0.4375047434889647	0.6594288633667197	0.2159750930948726
0.3815712544962248	0.1588147606887995	0.3270111265509043
0.4373775788645252	0.1600178312286582	0.2715022235610637
0.4932633959501348	0.1584552087550797	0.3267613829177612
0.2694291250089657	0.2695986166210760	0.3253483461799741
0.3247464761806333	0.2133047848052238	0.3251770498662870
0.3247457611175606	0.2691041597134645	0.2693583337873487
0.3797007314973832	0.2126971970096786	0.2690407097613792
0.3812937395064516	0.2697841071037536	0.3262362357638247
0.4951801995287751	0.2124696277181786	0.2689577611927970
0.4935151956584552	0.2690825809939030	0.3256291032306100
0.5504481151419993	0.2127745455788285	0.3249805413636082
0.5504998590381665	0.2686268243788404	0.2690822444219714
0.6063306338409393	0.2688391347788773	0.3249763286905235
0.1592005315693213	0.3820052470075695	0.3270454156525939
0.2136308027809776	0.3253735576812982	0.3253583242845314
0.2134312213177628	0.3800706385174605	0.2693096881400767
0.2693589911823933	0.3250701164695504	0.2695394341316367
0.2702098815384069	0.3821960133650980	0.3266929762718644
0.3812813761677495	0.3255168898873421	0.2690895228616440
0.3816739832637834	0.3819493417225039	0.3264832567321196
0.4374420204162618	0.3821473969638567	0.2695172066595197
0.4935811656523265	0.3254102431105627	0.2692830497175815
0.4928554634483512	0.3820431370771685	0.3264512090727668
0.5494828823198944	0.3815188877332321	0.2693100118640916
0.6063315665435881	0.3245642628425499	0.2690514208955019
0.6058511989764674	0.3815506210775734	0.3256455291827789
0.6623402111698456	0.3246892425544879	0.3249131396785838
0.6623776532750211	0.3797873087445145	0.2688100964437330
0.7165565817138408	0.3818724964907144	0.3266004847107145
0.1610687509670964	0.4374458928951349	0.2718157634935141
0.1591250795784378	0.4930000450520646	0.3269537471790989
0.2132267409779346	0.4949999514923621	0.2690928616494245
0.2698613794332757	0.4930573105254833	0.3263917588479028

0.3260563257938120	0.4376874986634239	0.3265565580458806
0.3820537171179935	0.4381318971086856	0.2698157467885156
0.4375454865796863	0.4933863136525009	0.2699580013936494
0.4930179191831083	0.4380558608679078	0.2698112973313588
0.5488201373590484	0.4936563326879778	0.2698671897552541
0.6053694785603466	0.4935707345098076	0.3261264460507220
0.6622179715968308	0.4952974264501535	0.2688477960695349
0.7146496179125930	0.4376374464601296	0.2713072254229201
0.7162471900215518	0.4933306137499586	0.3267583874464240
0.2134271560146208	0.5497104835826864	0.3252529148753774
0.2693279981083604	0.5498780037379498	0.2694172353190872
0.2694794718714092	0.6051259736489492	0.3254226144906833
0.3250380294388917	0.6053652968196690	0.2695300506422778
0.3820273227321255	0.5486969230051847	0.2702850701907405
0.3816461170028191	0.6048098296251142	0.3264315314277853
0.4929685137158594	0.5487908555394487	0.2702719613303306
0.4931975012111687	0.6047810227207044	0.3264950808466143
0.5499380909647616	0.6053928593359498	0.2696350935181139
0.6058112561875539	0.5500738724448163	0.2693594375793205
0.6055696433744514	0.6052506374928593	0.3253913239562257
0.6618445291660884	0.5500848196746979	0.3250771006765013
0.3249541140066989	0.6611935109994410	0.3253166907585177
0.3799031299618110	0.6614897033336262	0.2694370987774937
0.3817725754562757	0.7155803521412506	0.3272271109175733
0.4375055037868008	0.7137839432285715	0.2720673133506636
0.4950596518407943	0.6615096767051152	0.2694931998599566
0.4931688191396968	0.7156413664539478	0.3272046567142928
0.5499634193041513	0.6612221026463100	0.3253197994700846
0.2713267712057834	0.1616662478316146	0.4382028475820406
0.3260647588666169	0.1592843035225497	0.3824798684394982
0.3816814692457457	0.1558363599864532	0.4381508228617641
0.4373375982785313	0.1560626431610748	0.3827629536098621
0.4930116584688322	0.1561807988953092	0.4381495318672981
0.5490214992798270	0.1587401625535408	0.3822899825322580
0.6041512082315794	0.1608439110486406	0.4381402306282021
0.1614553944270354	0.2721358567534566	0.4381990312174380
0.2157124369138154	0.2162021089963020	0.4382717829533333
0.2136744647367850	0.2696624630512136	0.3807298280790616
0.2691005378940310	0.2138858747216651	0.3806674134286140
0.3263931924920229	0.2142116121931576	0.4384631737505569
0.4371572607030282	0.2694718426201322	0.3823014083174763
0.4929364339685041	0.2698640459168679	0.4381995792950944
0.5495146435574573	0.2693344402161025	0.3818237802753746
0.6065154472010941	0.2130378368794033	0.3804492773648078
0.6599421152948599	0.2154315201136081	0.4381697222098624
0.6621817502404617	0.2688826794244980	0.3805300526483361
0.7144195793684625	0.2714064623436975	0.4382054539534901
0.1595132319279056	0.3270208888424870	0.3825674029989404
0.1559692767232769	0.3824845397813752	0.4382355797763597
0.2700422807935394	0.3263918084377635	0.3822612592245606
0.6058323489606592	0.3256874840226486	0.3817923025122772
0.7163915531730426	0.3264326934151475	0.3823235481754387
0.7189205243006228	0.3823538922054457	0.4382788907078089
0.1559159268972730	0.4375594384419990	0.3826987866484834
0.1558253510156174	0.4927026592487563	0.4382492039132506
0.7190908187062336	0.4377120772383027	0.3826008754142888
0.7193334387217221	0.4931542999886208	0.4382243312545532
0.1593075258208653	0.5480619883785973	0.3825198393228880
0.1611791706518828	0.6030326220742600	0.4381168192758647
0.2135718755892796	0.6053229724365387	0.3806647942087710
0.2700188529749006	0.5484455331791767	0.3822792761830653
0.5488043805318094	0.6046521338635673	0.3822015415081714
0.6048684972330375	0.5485620651389932	0.3822962082742998
0.6614241601626089	0.6054863823935179	0.3806360815316879
0.7159472926054512	0.5485611660481376	0.3823915684118702
0.7137336599202376	0.6031987905876613	0.4381938736477063
0.2155737903286188	0.6589311866426575	0.4379901807261339
0.2691663334965224	0.6609792662695607	0.3806314145329208
0.2713995818819303	0.7133198136475768	0.4379499175271960
0.3265105356273523	0.6605776762604997	0.4383158619912564

0.3264066784334331	0.7152779982107087	0.3824453391620355
0.3820064408900285	0.7187645495543358	0.4378758611884415
0.4374148602295009	0.7187485574250494	0.3827567820067704
0.4927769252879896	0.7187433226371462	0.4378807975600247
0.5484589180627498	0.7153158105008124	0.3824347446672794
0.6057272538494214	0.6610321223262881	0.3806045138100824
0.6034149767841780	0.7132667743850785	0.4379519895326792
0.6591936547781003	0.6589107448301553	0.4380861337537515
0.3263290450830362	0.1592857984272787	0.4936255795006843
0.3816919305461968	0.1591025044986155	0.5485314722217974
0.4372532260971662	0.1557140680757553	0.4933541225642926
0.4929557423404060	0.1589189849071957	0.5486206187910865
0.5487432212503237	0.1592115613269803	0.4937563370941862
0.2144079559038814	0.2705642072268156	0.4951556622436636
0.2699966694891350	0.2147864541507264	0.4951103511260517
0.2705307477853615	0.2707825925841324	0.5492985267098414
0.3259499817297322	0.2152814393815377	0.5490860472837332
0.5493367523153267	0.2143216584759749	0.5497705755770708
0.5487788205952339	0.2702169793430508	0.4940839890708024
0.6062685381619820	0.2133673837208577	0.4957196031374258
0.6050564126169504	0.2701259519948364	0.5497960382994910
0.6618962119633155	0.2690724047656944	0.4958083577095341
0.1591526126450175	0.3272221702198954	0.4937182483928856
0.1592139257964017	0.3822341627980322	0.5489115893931075
0.2150392576505931	0.3264407977808350	0.5491795530951978
0.6049198182445064	0.3264361886781615	0.4942177946873899
0.6607561296718859	0.3259422719328567	0.5500701844922385
0.7158651714449610	0.3267946491512943	0.4940064434716558
0.7159851464152889	0.3823669350577059	0.5491480233887105
0.1559792239241165	0.4375709742468786	0.4935641210599568
0.1592592240552113	0.4930099833617643	0.5488288465681493
0.7193641369093289	0.4378699389403915	0.4936997704449176
0.7158625294841857	0.4932345776587652	0.5490539398431014
0.1590346236053742	0.5480738878973824	0.4936596205972166
0.2149980632287186	0.5489147725504580	0.5491531418407453
0.2143843538252928	0.6045667765688821	0.4949130907880521
0.2705236594292684	0.6043854219256203	0.5491391157150068
0.6043176567263957	0.6043958111406067	0.5491923308908840
0.6597751005023292	0.5489783988105806	0.5493030772568589
0.6604557580931806	0.6047070593264366	0.4950510562576129
0.7158993010165744	0.5483629472745644	0.4938657406535810
0.2700507083857331	0.6603182464889898	0.4948455729778941
0.3259912055261698	0.6599713705176444	0.5487777808497614
0.3265153505504513	0.7157443795822153	0.4932182334026153
0.3818416295501365	0.7158606788536194	0.5480569036867110
0.4373861354303260	0.7188681331524472	0.4928288388357538
0.4929307125004350	0.7159399073048658	0.5480678890146683
0.5487230460835918	0.6599985399249397	0.5487550860214193
0.5482440976504069	0.7157634930122800	0.4932548869020713
0.6047816294238062	0.6604059549320757	0.4949642197482727
0.4373448109789875	0.1612595345950704	0.6031596443874964
0.3258320534931233	0.2708809370482209	0.6042448904001226
0.3805797330527195	0.2150840262532601	0.6042596563491033
0.3804758402736703	0.2707601359342099	0.6595032609307190
0.4373829345803902	0.2174040338784989	0.6569013574676145
0.4940534882284967	0.2149921351412889	0.6041363569379362
0.4942293870193510	0.2708794667271086	0.6592850369388279
0.5488333770021926	0.2709710920936784	0.6036968493822190
0.2151600797391002	0.3810793645022839	0.6046102936353368
0.2708899762129411	0.3261962128524096	0.6042790963247374
0.2706234525160431	0.3805234886283824	0.6599003461082885
0.3258988156387603	0.3260642963059019	0.6597449965379424
0.5487792663651557	0.3261054605228432	0.6593698560762922
0.6039283360587214	0.3262462207978015	0.6038937851417507
0.6039936921851061	0.3806054447652740	0.6597262651396278
0.6597708930537580	0.3811730264250870	0.6046420211253282
0.1616670524396709	0.4376578749370501	0.6036570180849797
0.2173347364006812	0.4377177511113478	0.6579633408783492
0.2151391778668862	0.4943033961743059	0.6045585571304498
0.2705861586502346	0.4949471788821181	0.6599302939943702

0.6041014022119657	0.4948712549666638	0.6598924620191180
0.6573372630866312	0.4377547000410628	0.6579533092401526
0.6596383532791974	0.4943777389418244	0.6046608528999319
0.7133749665427112	0.4377894009327234	0.6039055374347946
0.2708778716156160	0.5491916773033757	0.6042772007843202
0.3258515171627580	0.5494010150057681	0.6597449037621755
0.3259053057285833	0.6043692081758305	0.6040621029266561
0.3804014968559802	0.6047190716883545	0.6594300569386942
0.4942354300751661	0.6046735333684964	0.6593597937807233
0.5488441166962088	0.5493684687139828	0.6596742840823179
0.5488067499308581	0.6043713330323517	0.6040197346614685
0.6039776906699158	0.5492618188231054	0.6043413712285219
0.3806290116579373	0.6603111273382885	0.6039980791441519
0.4373691304182311	0.6581133416420597	0.6568608927809158
0.4373889626097789	0.7138520417813899	0.6027931583962504
0.4940729749904157	0.6603059751473497	0.6040085987312231
0.4374017140334454	0.2721800743126490	0.7142654935294688
0.3279519987200979	0.3831396872804294	0.7138161192796451
0.3825756331107027	0.3281067098607992	0.7136839495917352
0.4374001004917646	0.3825545649715006	0.7147522757982064
0.4921931711783439	0.3280331236796284	0.7134488730575786
0.5466850292491470	0.3830484060961157	0.7136306424270206
0.2733189685976006	0.4377442330386545	0.7120995883108074
0.3280356764090392	0.4922718682466216	0.7137779707047905
0.3831269288141932	0.4376921438018065	0.7148391262411165
0.4373626239182245	0.4929067460299866	0.7147485930058143
0.4916000350745533	0.4376900542869268	0.7148201096258003
0.5466556099805859	0.4923160702329205	0.7137586236157428
0.6012640000372936	0.4376309674512927	0.7120898826002383
0.3825469783821023	0.5473640103925189	0.7135928158272254
0.4373265480522069	0.6031982284146005	0.7142155153311439
0.4921453970496095	0.5474130509212259	0.7135056459498743
0.4927840341797739	0.3260543769025840	0.3823136010806397
0.5487458251167369	0.3264235844750624	0.4383277851617260
0.5488634304948865	0.3822216214699193	0.3823839769226829
0.6052021807813677	0.3822745394475139	0.4382522606101796
0.6054693356761319	0.4380076068459955	0.3822707526237996
0.4373648861006570	0.4390449497033986	0.3292923867603472
0.4373795089774234	0.5477982732484961	0.3295528871583183
0.3833472500791811	0.4933408434008051	0.3294874702968847
0.4915328301203212	0.4933179594449687	0.3293750517277968
0.2720328165539087	0.4379517136913920	0.3845421874415608
0.3268749265872691	0.3828102605691318	0.3852348415710397
0.4364699758445956	0.3274579804392093	0.4393117840538548
0.3275520923453740	0.4921308820358920	0.3836878140639619
0.3821469882700225	0.4379063877192495	0.3838603351390923
0.4372905224604022	0.4375845283028986	0.4377333430386336
0.4920013404589892	0.4388755902285899	0.3838276289907431
0.5476091934584977	0.4385341386657555	0.4390931146175967
0.4372970392804197	0.5490740072492847	0.4378390234180015
0.4919635779461368	0.5474855031682154	0.3842238232149016
0.5469868500838433	0.5474409603065848	0.4391526790975306
0.3273721292177612	0.2735120065826310	0.4932941756157904
0.3281283708851058	0.3283783499335375	0.5475708866595880
0.3265978939543305	0.3823874527118514	0.4935669395497047
0.4373259512351160	0.3270593077026565	0.5486845902323328
0.4917734208126019	0.3279282107806593	0.4942632009732403
0.5463800323208552	0.3286598887260276	0.5480476243177304
0.3267125148720884	0.4930297783058165	0.4933740429411596
0.3823229854444128	0.4377667943724982	0.4932550936935415
0.4371211802864127	0.4377851000796332	0.5483994331893607
0.4926566451613697	0.4374185994443168	0.4929095368773106
0.5479714936168411	0.4376841867206613	0.5487633216078522
0.3283977349319761	0.5467864126937422	0.5473534684747072
0.3272801956879254	0.6015303288119471	0.4933907060864625
0.4372181243482179	0.5487192992979714	0.5488117879712765
0.4928396539160882	0.5486701651307332	0.4934799224323766
0.5464785498681665	0.5469364036370535	0.5475098210721361
0.4374161708135859	0.2738806154529679	0.6032177244768483
0.3284844640003295	0.3837551839073646	0.6025011495323120

0.3823983511794020	0.3823549942407513	0.6575760532591913
0.4372291281385707	0.3262324809136206	0.6582380069713161
0.4921346231368745	0.3824004457053484	0.6573931922511335
0.5461052007298063	0.3838751407492840	0.6024492659792099
0.2737298419961227	0.4377551840842258	0.6037523850205743
0.3285440623529499	0.4918223901992789	0.6026526403719452
0.3826127437735526	0.4929924514319826	0.6575857787617597
0.4374331709684297	0.4376370535628011	0.6568407819603501
0.4921449136301015	0.4931353954565829	0.6574861380916853
0.5471981494045809	0.4378289607444248	0.6575659456332735
0.5462149536421007	0.4918448614686835	0.6025527468927218
0.4372575901544851	0.5493816340946920	0.6581543468490100
0.4373036760909826	0.6016829555602020	0.6033434760259847
0.3812709731114121	0.2730113301340934	0.4391154833137604
0.2727261380200110	0.3825634235487044	0.4385643838752088
0.3272105405033714	0.3282016519805951	0.4393993819292423
0.3821444433261516	0.3274317017451212	0.3851464909968904
0.3821957928613401	0.3823630276504898	0.4383991119497146
0.4364143314050538	0.3830788420216314	0.3836741657982554
0.4912483474287372	0.3835190515247069	0.4387014119019151
0.2726490770918166	0.4926196120006368	0.4387712551384698
0.3262805963712093	0.4379024784348706	0.4380568239606781
0.3819745862148099	0.4929426492157253	0.4379168123110368
0.4377250783408854	0.4936217417023580	0.3832645264051287
0.4927152219979141	0.4929063681200658	0.4382083814411641
0.5469820106399654	0.4927606813618020	0.3842089551758837
0.6020998583034293	0.4934813486182972	0.4394792360286688
0.3276048188501391	0.5468098768058135	0.4390897467518779
0.3829046239952707	0.5476731388114144	0.3840801614801161
0.3819353162244240	0.6020650109178660	0.4383881439187529
0.4374701787830229	0.6020292071033112	0.3831123367197949
0.4927036212896492	0.6022953872222609	0.4383109693681493
0.3825006173525602	0.2737435589993313	0.5484992847677433
0.4365060104863572	0.2733153523557847	0.4942069706912719
0.4912894925447221	0.2734308373542275	0.5486970178843391
0.2733198904352507	0.3276534284079440	0.4938597191053863
0.2734852543407799	0.3827435332611520	0.5485729582171519
0.3816521852470787	0.3269414321249011	0.4931358571880580
0.3820359629496413	0.3827025303403730	0.5486289335623882
0.4374865064304614	0.3822639056988788	0.4927291946020824
0.4924066051327868	0.3826032349173021	0.5482388200867077
0.5472421135322413	0.3834337241584301	0.4943106547962470
0.6015353241253413	0.3837547852492589	0.5489814972908542
0.2731930430078928	0.4375433170224924	0.4938090018491302
0.2734868811102120	0.4927861180064743	0.5485465967317256
0.3262669514771191	0.4378307422077386	0.5490200993215614
0.3823053817549356	0.4929244545948278	0.5483969943712452
0.4372343271251274	0.4927715400378194	0.4930454723842343
0.4922732389653231	0.4931249384117830	0.5484958928955485
0.5480316951254574	0.4933656626269663	0.4931326290752113
0.6017253803444379	0.4386157537524186	0.4943807590079840
0.6011927532273379	0.4926153870765272	0.5485445114532160
0.2733629538970750	0.5476069201686381	0.4936179841192701
0.3818732780838875	0.5484157910899629	0.4932737359505069
0.3823630861024960	0.6017350999537316	0.5483689662345390
0.4373917286917587	0.6018180756557184	0.4937097924240175
0.4923030595907246	0.6017051706294351	0.5483909283683284
0.5474826568306391	0.6016382400439386	0.4935271536694967
0.6015094900329196	0.5476247190094116	0.4934235096293304
0.3833282257916689	0.3285709769250448	0.6025618490395146
0.4370766799249353	0.3821470926069439	0.6041158889903749
0.4911805590078638	0.3286499000821322	0.6023579654295529
0.3273146447929945	0.4377648158367904	0.6575839746124320
0.3820032835398958	0.4378135097785814	0.6039995615756952
0.4372662789693260	0.4932744685088202	0.6038268745711323
0.4927236293335059	0.4379863164054252	0.6041646451297044
0.6009730086883577	0.4376691319708393	0.6034973779535722
0.3833819888791383	0.5468153701582676	0.6025966724165067
0.4912382824459463	0.5468648184283138	0.6024058614126999
0.4374531952003753	0.2366482602572073	0.7593264593052694

0.4374254569000152 0.6385380265743104 0.7594149225936477
0.4375562834114882 0.2133104211281144 0.7868882159631477
0.4375059230763131 0.6617832065391941 0.7870576334913681

2CO(Co)/$Cu_{303}Co_{102}$

E^{PBE} = -1735.5154 eV, magnetic moment = 159.96 μ_B
CO2Co-Cu303Co102g300fin-1735.5154m159.96

```
Cu Co  C  O  Cu303Co102CO2-Co
   1.00000000000000
    32.0000000000000000    0.0000000000000000    0.0000000000000000
     0.0000000000000000   32.0000000000000000    0.0000000000000000
     0.0000000000000000    0.0000000000000000   32.0000000000000000
   Cu   Co   C    O
   303   102     2     2
Direct
  0.3814154331224089  0.3815483206423533  0.2123481553798367
  0.4372772534307446  0.3253275214672939  0.2129829307068729
  0.4932724601534239  0.3814883023237695  0.2122529918984412
  0.3814011907090862  0.4932910522498168  0.2123038018184642
  0.4372680296987416  0.4373600153891727  0.2115100233588896
  0.4934319508797759  0.4934081524820394  0.2120439129173193
  0.5498117327012200  0.4374521667019689  0.2126258919174532
  0.4374100081262593  0.5498031796070522  0.2127086915032480
  0.4372446719520381  0.2129379265340193  0.3260205461622290
  0.4371042507479583  0.2695238271488219  0.2698427308944317
  0.3257414621137311  0.3260811058409218  0.3266277607485077
  0.3255248097565500  0.3817591593350829  0.2706023860406824
  0.4370870087725978  0.3260909476367136  0.3264168504858410
  0.5496596814052311  0.3253352722477891  0.3255367968098726
  0.2141822894269043  0.4379203665043700  0.3268420940157089
  0.2702540480992039  0.4372698454104194  0.2706568281920717
  0.3255951557093145  0.4930848728600130  0.2699981476939698
  0.4373127753137764  0.4376957529244329  0.3264995161812626
  0.5490826725486575  0.4376364579057643  0.3259494315646783
  0.6058156462574859  0.4375439214043314  0.2693976127789564
  0.6619952030397647  0.4376589913905093  0.3260526391157504
  0.3260605980643791  0.5492810328300818  0.3262225617770301
  0.4370846685712091  0.5490413612834888  0.3268351220541060
  0.4373545114154592  0.6055601820658491  0.2698378439571834
  0.5488136774221745  0.5494104831494698  0.3261205493230339
  0.4373159560850450  0.6618359677232715  0.3262044300535325
  0.2706996338412916  0.2706316442310971  0.4382443543076481
  0.3258041250024368  0.2703192344814847  0.3826110543436212
  0.3814931306902660  0.2123158387916466  0.3822685782602559
  0.4371631908010763  0.2116072623931353  0.4380690849569512
  0.4930385799038104  0.2124280426377888  0.3822205055427753
  0.5490551366042805  0.2129730379341946  0.4381291666729867
  0.6049913041397877  0.2696744038248520  0.4382797999883576
  0.6614502081118483  0.3260221744292477  0.4384014330872032
  0.6622204456871436  0.3820441056885466  0.3824429528183375
  0.2141628834076555  0.4930623223741757  0.3827838014422100
  0.6621609448322857  0.4380531405051964  0.4390146613743849
  0.6616084197964720  0.4932682229766382  0.3830827202011922
  0.2146137338058363  0.5486128714636561  0.4382696009267030
  0.2705642714308172  0.6045919985773275  0.4382307212791023
  0.3258919551875211  0.6051414165710092  0.3823924931997195
  0.6042766968312963  0.6044345013195314  0.4381767385480807
  0.6608301038065626  0.5484203617594032  0.4389833235599856
  0.3817779223055033  0.6617981349935633  0.3829595837065050
  0.4371627363249423  0.6617684730723848  0.4386093893026176
  0.4927314557603600  0.6616671751567242  0.3830383398925609
  0.5481174147574362  0.6609177295765877  0.4386529697939361
  0.3816261219731101  0.2123739297333866  0.4936106209291741
  0.4372310403381241  0.2129830266258271  0.5492274768559983
  0.4929265657193875  0.2123637720151973  0.4936531160341318
```

0.6608211641583842	0.3826166038356846	0.4946296724444237
0.6599973782052749	0.4377985256387513	0.5496131060239564
0.6611398007902495	0.4930202656789530	0.4944213580139936
0.3819700013376389	0.6611159464852416	0.4935755369734350
0.4371500301412973	0.6605994326310139	0.5486843237337178
0.4923918588492877	0.6610425249967603	0.4935898538603142
0.3794946906613718	0.2686056622615467	0.2133304806167603
0.4372010361238091	0.2150098416271533	0.2154869777536757
0.4372494509555115	0.2706287876492542	0.1607255022275556
0.4950994925950413	0.2683853247979587	0.2130790417368645
0.2686999413495071	0.3797892538509883	0.2135720668741029
0.3243379014117414	0.3245417455982013	0.2135377074504365
0.3255712853201857	0.3813461201133407	0.1588503636077532
0.3812058358726622	0.3256691562723285	0.1587014946991162
0.4373325563993432	0.3813708681579888	0.1555211416106709
0.4934988452131237	0.3256471221275540	0.1586484790989749
0.5507662082407572	0.3240953473193001	0.2128465947648384
0.5493579220959468	0.3811943408633388	0.1586815957402918
0.6068571199315549	0.3794906548893600	0.2127259499333356
0.2152633510106423	0.4374041920071907	0.2161802663083706
0.2705602995769933	0.4374818651841769	0.1609680608627104
0.2685713508454685	0.4952099258181893	0.2134434456070437
0.3254629054070091	0.4374992186442667	0.2129487696177974
0.3255964399598587	0.4936163287894616	0.1587179596474979
0.3814215875037376	0.4374488125569599	0.1554429840214194
0.4373317430299818	0.4934840922746078	0.1555891475283691
0.4933532579999835	0.4373390645385139	0.1555618215664958
0.5493706374174112	0.4936654990344778	0.1586013581826211
0.6045111950569902	0.4373824612527047	0.1603670527147450
0.6068055506530070	0.4954363148595645	0.2127264231712943
0.6602662503213588	0.4375601925052340	0.2151329336629261
0.3241896197552703	0.5506007589560807	0.2131257577154875
0.3812081049592865	0.5493208516248628	0.1587770035967282
0.3794955975388412	0.6067577010078478	0.2129997815750116
0.4373605960063455	0.6045074680559335	0.1605401945324238
0.4935748663187161	0.5494431695189207	0.1586015298056568
0.4951387627777855	0.6067193112522111	0.2130187610595572
0.5507372274214121	0.5508029482979172	0.2127721408548337
0.4373091125105132	0.6601435670620313	0.2155237976062780
0.3813712848081410	0.1585007300294521	0.3264613631200563
0.4372850013555416	0.1605324314093778	0.2713926195938151
0.4933068850877766	0.1585900851218551	0.3264874065549219
0.2698974947909522	0.2700419680765757	0.3262572732339964
0.3245250563375035	0.2130977607353747	0.3252211602453788
0.3246148410140580	0.2692391687713426	0.2697175175312470
0.3794472931545154	0.2129099411815881	0.2690957220282946
0.3811213581219829	0.2697498739731980	0.3265628417742184
0.4951053757195557	0.2127761407648309	0.2688673809857101
0.4934187444702181	0.2691601199544050	0.3257646144933991
0.5505461375396096	0.2126855964672523	0.3247416680177125
0.5504510080587856	0.2686292100257806	0.2690277721015638
0.6062213363202378	0.2687563352302012	0.3250004380291654
0.1603633771545197	0.3824651721614897	0.3284534410194576
0.2145619894580208	0.3258276764866685	0.3263800991449589
0.2142870813808729	0.3805118723296257	0.2706715783314628
0.2695659232505980	0.3256780079749659	0.2705489794635146
0.3810136905337934	0.3260169477177781	0.2700373741319998
0.4367742307809219	0.3815554532417844	0.2695315621341216
0.4935412829106887	0.3253625368075000	0.2692574530981633
0.4931053829423745	0.3819891754208587	0.3262233074253986
0.5497892756641336	0.3812323540352929	0.2690231896468493
0.6064266205976043	0.3243984121692809	0.2689140191158991
0.6058970546453327	0.3814420248905654	0.3256895770890347
0.6621856205260974	0.3245021939715553	0.3248878982198744
0.6623772445644427	0.3796519688459464	0.2687012651446448
0.7161830261022580	0.3817239588118461	0.3266557691818839
0.1610230705694552	0.4373655789194836	0.2723552744304267
0.1592825787945405	0.4929301091791668	0.3275800164416290
0.2134473593408883	0.4950285945042270	0.2696202873456404
0.2703683716107160	0.4933515743787736	0.3265349168726558

0.3809664055164179	0.4371050113767025	0.2699295938935491
0.3816149821911737	0.4932606530119293	0.3266583757863157
0.4372781211414698	0.4935108405692900	0.2688903827120050
0.4934410154600195	0.4375355298934003	0.2687886392095525
0.4929091058084522	0.4933859871241315	0.3266606628236441
0.5496709478461175	0.4938519880985088	0.2690919865868329
0.6058077101557395	0.4937844501889151	0.3255452418708383
0.6623175051777459	0.4955545267726901	0.2686186285916754
0.7144967273350923	0.4376119488011446	0.2714925082334782
0.7158748970576785	0.4935369793359937	0.3269056570443948
0.2136536891671392	0.5499867038206734	0.3255033829531989
0.2691330569175401	0.5502327745367477	0.2694885528730834
0.2694408093416266	0.6057009513421073	0.3253748092388777
0.3246219255663195	0.6061385719976992	0.2692503611938117
0.3812588876818638	0.5496319907174591	0.2691734654899450
0.3812342506279801	0.6052831188783846	0.3263108474259551
0.4934458796104463	0.5496156428088008	0.2692108036483394
0.4932576894286329	0.6050448169683964	0.3265103454006341
0.5501229964298291	0.6060759185113993	0.2692631646520761
0.6060686630190540	0.5505607893412900	0.2690261501070104
0.6054897889458903	0.6056750271686755	0.3253472389697971
0.6617570104167642	0.5504387708849580	0.3250533774040980
0.3247457606061804	0.6619168275306824	0.3251572231336060
0.3796636497628992	0.6621773543698054	0.2691049400858945
0.3816680412315281	0.7161414973258271	0.3269631166601024
0.4373973985896401	0.7145791118199261	0.2715151280322949
0.4949359550181910	0.6619864607614409	0.2692748617406653
0.4930239974107438	0.7161007176537761	0.3270837436063735
0.5498710805659264	0.6616221487167288	0.3253660899087521
0.2709542169962930	0.1603675200557932	0.4379822200869899
0.3258717560368056	0.1582941361806638	0.3820882524604715
0.3815812144765076	0.1551187006348864	0.4378239792961138
0.4374029671001277	0.1554514043275742	0.3820994146613363
0.4930769220480368	0.1553496977076642	0.4378056507793796
0.5489330931319929	0.1586520548170350	0.3820823108691335
0.6040500422497795	0.1606895157827256	0.4381064704163092
0.1631083105328497	0.2732108110430981	0.4383589343738601
0.2160861590188653	0.2157498911008846	0.4381117956407680
0.2151732243476023	0.2703459442667928	0.3814642596731427
0.2691241560272788	0.2130878740705353	0.3805499654612364
0.3257276020460942	0.2127928248916461	0.4381181750632417
0.4368930158427559	0.2696683992335006	0.3824860788315348
0.4928686995593412	0.2699091148805400	0.4384199361903480
0.5493598734277576	0.2691816559933400	0.3818583353306109
0.6063004855169180	0.2129405188407741	0.3804079147858878
0.6597050113897012	0.2153562078963358	0.4383018141938517
0.6619563657505777	0.2687530781830421	0.3806185816588378
0.7141373142664554	0.2714616463626665	0.4384851715231537
0.1607889204947812	0.3279394384340518	0.3835311612066450
0.1590469671134251	0.3827871876302444	0.4386664576846571
0.6056682556311659	0.3255085083731251	0.3818715070205275
0.7160307549775465	0.3262917432227918	0.3824275280281618
0.7184342640241068	0.3820590730470250	0.4385638644676649
0.1581158821478991	0.4373756366559383	0.3837655679058785
0.1582449002137161	0.4923558657667064	0.4387835519221962
0.7187642475804076	0.4377162597654076	0.3827469534773476
0.7190432318689265	0.4933479396772217	0.4384509183465754
0.1594937315671933	0.5481236258766052	0.3829765948288313
0.1614220110610702	0.6032492846499284	0.4382686168260574
0.2139321003803969	0.6055565693394552	0.3808529428714733
0.2705768903459257	0.5487316143045143	0.3825391696873278
0.5487260136060134	0.6048474314555463	0.3825307399040439
0.6049955940918690	0.5487805636050449	0.3825952864153659
0.6610771882559225	0.6056976549229848	0.3808444419894190
0.7155656360577533	0.5488098889578925	0.3825577726887919
0.7133664460389187	0.6033160017373318	0.4384311306752984
0.2157181753418889	0.6592413914133192	0.4382613838872362
0.2691900260343709	0.6615798275120025	0.3806718803427013
0.2714693622498497	0.7138561891598147	0.4382057924400322
0.3264438735892059	0.6611746117000752	0.4385780642306522

0.3263719592052449	0.7159790198363430	0.3825549162362422
0.3820342184279490	0.7192811473088077	0.4381886120662062
0.4373037787489340	0.7188477726452907	0.3826707185216958
0.4925238900054489	0.7190914374545280	0.4382598061889177
0.5482394879567634	0.7156929693932497	0.3826783543166068
0.6054821321264260	0.6613295822181294	0.3807767554806560
0.6031560268410100	0.7136046401908640	0.4383001554054777
0.6588721429250177	0.6591106989645293	0.4384394720184306
0.3261667823242956	0.1580555726763039	0.4937283951354571
0.3816854366530439	0.1580946401616809	0.5492382255703172
0.4373296829580033	0.1550298771966170	0.4935257573733054
0.4930708914586220	0.1581873565521451	0.5492323613303750
0.5487499357387052	0.1584430941614775	0.4937748868014504
0.2153801362741923	0.2700271425381618	0.4951003982763296
0.2694267100438766	0.2129865093321519	0.4956718288726277
0.2710437215662221	0.2704045213305467	0.5493518540366250
0.3250397373991658	0.2130759613756334	0.5503617851822937
0.5498528534262350	0.2128506469429834	0.5504686099326995
0.5486960725009048	0.2701704180645211	0.4946918953147939
0.6058633726964060	0.2132483779704367	0.4958081206889429
0.6045562531851096	0.2699927527515613	0.5503403856262959
0.6611780289137871	0.2691092531797256	0.4957569157752439
0.1616945536130840	0.3277534754960393	0.4933011020324505
0.1614354300096766	0.3826334557296849	0.5481241426211813
0.2154309436990474	0.3256762547858574	0.5498602649716336
0.6046791780275165	0.3264032661967522	0.4943822315116324
0.6600388237682356	0.3259428237415687	0.5507353514739901
0.7149655636924750	0.3264868983991457	0.4945720085472228
0.7153470605238612	0.3820860763049738	0.5496220923101133
0.1590235121364054	0.4375914690293071	0.4935591103715044
0.1606961365664863	0.4927315424653044	0.5482682113473609
0.7189371200600094	0.4377791373148137	0.4941000636248655
0.7155282548708122	0.4933915126716804	0.5496021502325215
0.1605492216365577	0.5475369990202948	0.4935314583665542
0.2150627121074210	0.5494057023757497	0.5495909355731777
0.2144699958891201	0.6051080312307067	0.4951795046863664
0.2704947333466897	0.6046552710802845	0.5492762056965014
0.6041545296303820	0.6046730285105545	0.5496590176496100
0.6597799374634316	0.5491878291846318	0.5498597075471281
0.6601966418430850	0.6048921675401012	0.4954587631501128
0.7157475696984104	0.5486223368788355	0.4943085632707455
0.2700126139448872	0.6607593798176989	0.4951387275714417
0.3257962293436059	0.6602958105173338	0.5490930739344911
0.3265190909553270	0.7161846698082741	0.4936913893952963
0.3816596949889649	0.7161710718680960	0.5487938442231380
0.4372630264288520	0.7192956816441862	0.4935083541781502
0.4927876436191730	0.7160595978016929	0.5487049651970550
0.5485706398443980	0.6601514133780376	0.5490539925315835
0.5479519529545924	0.7159831309338183	0.4936548827155965
0.6045268711903630	0.6605163688669586	0.4952338535605427
0.4374525668736660	0.1606408119936862	0.6042053127279571
0.3261796326753013	0.2705643752007680	0.6042892931366751
0.3800249370869209	0.2133220612762195	0.6057228379894392
0.3806102504267507	0.2703265006385650	0.6598393185200524
0.4373984386176926	0.2159608093268604	0.6590829701851618
0.4947576350591386	0.2134794300833444	0.6056977745836735
0.4937958626844648	0.2706958103866132	0.6595286032030256
0.5487651793300665	0.2708071425862417	0.6044093652463018
0.2155383563555871	0.3806698368819068	0.6048726580523391
0.2713244217639910	0.3261863472586651	0.6042543357723180
0.2709295954518310	0.3807301497671516	0.6599532656055960
0.3260079714639286	0.3259841588810449	0.6598928684140668
0.5490711940877938	0.3256319060806636	0.6601347678741146
0.6591489504447213	0.3808580549015670	0.6053255310168556
0.1627968656265342	0.4375422483775218	0.6024678407999021
0.2176566715582499	0.4376127825040271	0.6579695241488461
0.2151593717924522	0.4948475580059873	0.6050190131238606
0.2704550532070636	0.4949664204161250	0.6600780294436667
0.6041532258726013	0.4949352022253528	0.6599961039000906
0.6568639771957893	0.4373565246270533	0.6579810439396615

0.6593926749390447	0.4941968315210451	0.6050948641718641
0.7131237950558396	0.4376685801057099	0.6042154552860870
0.2711373830465569	0.5488882742187553	0.6039006595765736
0.3256165142703670	0.5494015177688005	0.6597126078245957
0.3256580210921444	0.6045066542934435	0.6041472305442235
0.3799007934905518	0.6049179345766782	0.6598623965258535
0.4942643342784003	0.6047389293066465	0.6596639334232718
0.5484924769838114	0.5491678457196444	0.6594995654533390
0.5486040693133069	0.6045049140712714	0.6042316853324218
0.6037803534874877	0.5492228458400100	0.6044224627169803
0.3805397241543881	0.6602466906191026	0.6043460966059090
0.4371330767308944	0.6580169009358445	0.6577586305974784
0.4372422629082250	0.7138046711698453	0.6034256964398347
0.4937172907544448	0.6601686113299722	0.6043847674188558
0.4373631859429749	0.2733827148833577	0.7120023793474765
0.3279330413132399	0.3830164941530260	0.7139238164141997
0.3828788282580411	0.3282075176993225	0.7136962922842961
0.4373731299104484	0.3835472402300821	0.7150204523944255
0.4916272714972084	0.3282699227103927	0.7136258943777756
0.5467660288044762	0.3827216781737725	0.7129894917665385
0.2733599733026701	0.4377341621805461	0.7125187203297023
0.3276639763165454	0.4927765504288287	0.7139990254146843
0.3824156321966287	0.4380138703563835	0.7151897514429092
0.4372606991518093	0.4924504237757222	0.7149198683055726
0.4923023986936393	0.4378957270612494	0.7149295797411613
0.5468742769303067	0.4926162091804762	0.7134915083616508
0.6009707823690129	0.4375712503397217	0.7118921638281989
0.3825896457948900	0.5474673384607087	0.7136444066693700
0.4371732085121118	0.6020771127344262	0.7119915019797295
0.4918399713994073	0.5474302486378092	0.7137275651555218
0.4930429323307827	0.3262172535059083	0.3823674786538446
0.5488395252109508	0.3262883023782275	0.4383570146977854
0.3261443937103876	0.2706335584467067	0.4936560042529139
0.4372966086516251	0.2709720950774445	0.6041818143123261
0.3814026168204650	0.2703302155309812	0.4381659002764994
0.5490033414938186	0.3821985164220990	0.3825372153433115
0.6050487049094059	0.3823811390623064	0.4385136054140515
0.5489733695644874	0.4931498958227510	0.3825988319750702
0.6054708885456521	0.4380944533159054	0.3826093816061344
0.4370453614742140	0.6053675450425015	0.3826460949113171
0.3817536889254813	0.2708124615103017	0.5489207558201268
0.4372898033982935	0.2705836391314259	0.4937406539244851
0.4930671455549461	0.2707751731767984	0.5493272520747535
0.2168499036041442	0.3283468532410447	0.4382479773155949
0.2166631476839010	0.3827619239677756	0.3841470884191683
0.2166996330067897	0.4374671729272136	0.4391449791793189
0.2175232484896326	0.3832073262127504	0.4933788395685701
0.2171925915927923	0.4381072235320295	0.5485381337321864
0.2167472090586666	0.4919400946789210	0.4939554881056061
0.2704582834846649	0.3826991526722071	0.3300685270954065
0.3810570925940601	0.3824360769374216	0.3299392924352395
0.3256141261410427	0.4360622852021449	0.3294199734273113
0.2705716892305413	0.3291965040386780	0.3838844340710677
0.2710234530883896	0.4368138216766485	0.3839079174328323
0.6033161083117560	0.3266976511252111	0.6047665650181439
0.6038512001150913	0.3800748808620590	0.6601559419815407
0.3258096393379500	0.3825194851300737	0.3835688200333722
0.4363868067408084	0.3286995290956283	0.4381711932056301
0.3271230103259195	0.4919224676553712	0.3837845708564483
0.3807567514721742	0.4371195392003784	0.3834602903499626
0.4370290192655278	0.4376466939092040	0.4380526429139666
0.4916234892952107	0.4372598512020066	0.3850895845875252
0.5469172978572111	0.4382727684063429	0.4393554219008061
0.4370054773834834	0.5488125588150117	0.4380477922832366
0.4921660429447922	0.5484464279686956	0.3855807655980370
0.5469987275401307	0.5476686251178157	0.4398698393759187
0.3271303451716537	0.3287665756817709	0.5480664479987001
0.3262580596639336	0.3821021076023456	0.4934937243997366
0.4375065327495247	0.3293952546747580	0.5494160478377673
0.4923164788115565	0.3287708840710060	0.4939880117999295

0.5472221612148702	0.3297397679476509	0.5489198110433268
0.3263566336501036	0.4931226196929177	0.4933303371746240
0.3820104264014647	0.4377861703769936	0.4932991058653495
0.4366475125808881	0.4379657496702539	0.5483363041597287
0.4921892756668310	0.4375750426239863	0.4931381909175762
0.5479185127479630	0.4377466248279756	0.5488941795934602
0.3278540998522852	0.5470401692920882	0.5471586706932932
0.3272853445122664	0.6018986043841010	0.4934906298824681
0.4369777813893850	0.5489014544907881	0.5488208110647054
0.4924115559307810	0.5487225253215470	0.4937849958167127
0.5465638180207654	0.5473932629317247	0.5476302964021258
0.3281771581053516	0.3840467982452605	0.6027017159689551
0.3822302858774873	0.3832039113475407	0.6573968974439522
0.4370750086549250	0.3286014152866201	0.6579649618367764
0.4925472612926351	0.3831259365525090	0.6572706994384013
0.5476822569870838	0.3833268956429968	0.6037186982959724
0.2728517355231692	0.4380661575944353	0.6027187472164094
0.3280240507914624	0.4915459729950824	0.6026303573032320
0.3820653085897841	0.4928050550268480	0.6575364878587859
0.4371303597741977	0.4378869842606142	0.6571386188650209
0.4916751849137176	0.4931242381459607	0.6573173989857489
0.5471299240095986	0.4381765612766735	0.6570347217573064
0.5462211012562804	0.4918603053090266	0.6025950311333648
0.4370992900535155	0.5482256670676920	0.6574722697401036
0.4369354203137408	0.6017236911635937	0.6032848089865603
0.2708757543071723	0.3827989012202636	0.4382238330965696
0.3254423722771522	0.3289103542559687	0.4380211332256294
0.3815663936890877	0.3287988051567628	0.3833342204527434
0.3815844026601272	0.3822523172505362	0.4382010668370662
0.4361514967729070	0.3831117687759320	0.3841917395850949
0.4913190031713849	0.3832006165370126	0.4395770657896844
0.2716218756631707	0.4919605840152960	0.4393588861533381
0.3261285610549865	0.4378178689202422	0.4374658921581617
0.3818800491654923	0.4929001938764713	0.4379394805015946
0.4374726524130700	0.4935723211074083	0.3850862491084707
0.4921177465455127	0.4927976445466445	0.4385365062393898
0.6028491728264075	0.4934388398661260	0.4410648794983519
0.3272114744091049	0.5472644372239250	0.4399614102270594
0.3811937401884352	0.5478065920088737	0.3851705415019684
0.3808896797363086	0.6020310343951030	0.4393480302659116
0.4930493453162436	0.6023267948598533	0.4394693669720666
0.2712626369456533	0.3290571796240361	0.4931383818621610
0.2721103972866534	0.3838948893868993	0.5473925412873450
0.3815362661398369	0.3288648240080398	0.4935184038378942
0.3818662040217154	0.3822718079225014	0.5485191216728388
0.4371229082274547	0.3820307907396044	0.4933587967392625
0.4924178547553912	0.3824238178266041	0.5484412458597907
0.5471147792607839	0.3836416373188601	0.4943374122461162
0.6011586824545639	0.3833541841123465	0.5496517345806721
0.2710392453042626	0.4380369891575939	0.4935247441560749
0.2723372465855444	0.4914373199506205	0.5472248273041176
0.3263842868716703	0.4378307885071884	0.5488100812106795
0.3820027090396041	0.4931370863345459	0.5487738700038355
0.4370371800947093	0.4930390233325151	0.4927796109664922
0.4923156258614392	0.4933403940013488	0.5485108057538569
0.5475920230830382	0.4931704043640462	0.4936849580163478
0.6018055265527047	0.4391344742422291	0.4945527507831648
0.6009715253124666	0.4927448827647074	0.5484500032321621
0.2721691791098739	0.5468128318331809	0.4936617925139640
0.3817274608265046	0.5483434365753642	0.4934720190260720
0.3821272941793358	0.6019165232050280	0.5483981555265630
0.4370287085882266	0.6021868007826965	0.4935755455886519
0.4919196154834691	0.6019031098898997	0.5484593635802536
0.5472753470519821	0.6016635049056673	0.4934312949200881
0.6015902820688027	0.5476834333726265	0.4940274922325831
0.3824548983570620	0.3291954596464923	0.6037544135144904
0.4372751154237154	0.3827175059836906	0.6039616900562564
0.4924256151885359	0.3296811470826084	0.6037184099397583
0.3270889628515832	0.4379864345284566	0.6578878784751356
0.3816881082302031	0.4381037493941066	0.6039299328986267

0.4370041539011479	0.4933127463446590	0.6038978983448854
0.4923704373505070	0.4381890887222317	0.6040197441588195
0.6006248661298993	0.4374937475247654	0.6034548723735756
0.3827520852704083	0.5470644121460652	0.6027020601990363
0.4910974556126881	0.5470339667516197	0.6022566483282653
0.6340396098870711	0.2873764428880428	0.6264341960128521
0.6358282739886377	0.3582556158116781	0.6982033241925716
0.6550630235182899	0.2625964008706155	0.6434143997523603
0.6571261841912308	0.3419209178875315	0.7231655834521742
